# Oxidative Fluorination of Selenium and Tellurium Compounds using a Thermally Stable Phosphonium SF_5_^−^ Salt Accessible from SF_6_

**DOI:** 10.1002/anie.202209067

**Published:** 2022-09-12

**Authors:** Tobias Eder, Florenz Buß, Lukas F. B. Wilm, Michael Seidl, Maren Podewitz, Fabian Dielmann

**Affiliations:** ^1^ Institute of General Inorganic and Theoretical Chemistry Leopold-Franzens-Universität Innsbruck Innrain 80–82 6020 Innsbruck Austria; ^2^ Institute of Inorganic and Analytical Chemistry Westfälische Wilhelms-Universität Münster Corrensstrasse 28–30 48149 Münster Germany; ^3^ Institute of Materials Chemistry TU Wien Getreidemarkt 9 1060 Vienna Austria

**Keywords:** Fluorination, Phosphines, Selenium, Sulfur Hexafluoride, Tellurium

## Abstract

Fluorinated group 16 moieties are attractive building blocks in synthetic chemistry but only few synthetic methods are available to prepare them. Herein, we report a new oxidative fluorination reagent capable of stabilizing reactive fluorinated anions. It consists of an SF_5_^−^ anion and a chemically inert phosphonium cation and is exceptionally thermally stable. Accordingly, it was used to generate the SeF_5_^−^ and TeF_5_^−^ anions from the elemental chalcogens and to prepare the unknown tetrafluoro(phenyl)‐λ^5^‐selenate PhSeF_4_^−^ and ‐tellurate PhTeF_4_^−^ from the corresponding diphenyl dichalcogenides. In addition, we show that further derivatization of [PhTeF_4_]^−^ by oxidation to *trans*‐PhTeF_4_O^−^ and subsequent alkylation gives access to a new class of *trans*‐(alkoxy)(phenyl)tetrafluoro‐λ^6^‐tellanes (*trans*‐PhTeF_4_OR), thus providing an approach to introduce the functional group into organic molecules.

## Graphical abstract

An SF_5_^−^ salt containing a chemically inert phosphonium ion proved to be a powerful oxidative fluorination reagent for the generation of fluorinated selenium and tellurium anions, derived from the elements or the diphenyl dichalcogenides. The new anions PhSeF_4_^−^, PhTeF_4_^−^ and PhTeF_4_O^−^ were synthesized and characterized. Alkylation of the latter provides access to *trans*‐PhTeF_4_OR, a new class of fluorinated building blocks.

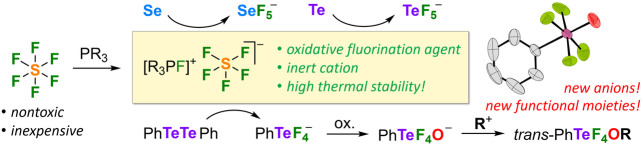

## Introduction

**Figure 1:**
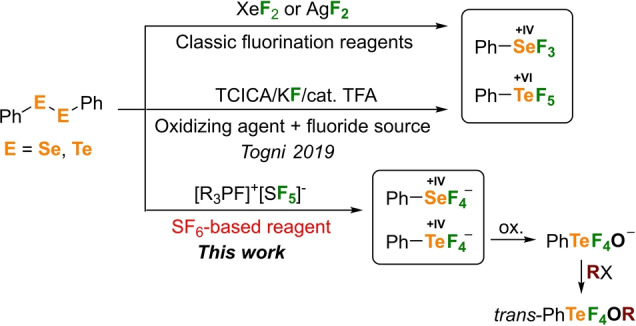
Methods for the oxidative fluorination of diphenyl dichalcogenides. Neutral trifluorophenyl‐λ^4^‐selane and pentafluorophenyl‐λ^6^‐tellane with classic reagents or Tognis method (TFA=trifluoroacetic acid). The salt [R_3_PF]^+^[SF_5_]^−^ (R=1,3‐di‐*tert*‐butylimidazolidine‐2‐ylidene) gives tetrafluorophenyl‐λ^5^‐chalcogenate(IV) anions, which can be further derivatized.

Fluorinated functional groups of the heavier group 16 elements are gaining increasing interest in medicinal chemistry, agrochemistry and material science due to their steric bulk and electron‐withdrawing properties.^[1]^ However, their preparation is often not straight forward and requires hazardous reagents, special apparatuses and safety precautions.^[2]^ Classical synthetic procedures to fluorinated heavier group 16 elements involve the use of powerful fluorination agents such as xenon difluoride,^[3]^ halogen fluorides,^[4]^ AgF_2_ ^[5]^ or, for deoxyfluorination reactions, SF_4_.^[6]^ The oxidation of group 16 elements with chlorine gas in the presence of fluoride salts is an economical alternative and thus particularly desirable for large‐scale industrial applications.^[7]^ Recently, Togni and co‐workers developed an elegant chlorine gas free approach by employing the solid oxidant trichloroisocyanuric acid (TCICA) and KF to fluorinate various iodine‐, sulfur‐ and selenium‐based functional groups of aromatic compounds.^[8]^ For example, aryl‐TeF_5_ and aryl‐SeF_3_ compounds were synthesized from the respective diaryl dichalcogenides (Figure 1).[^8b^, ^9^] Previous syntheses required the oxidative fluorination agents XeF_2_ or AgF_2_.[^3g^, ^5a^]

Sulfur hexafluoride is a chemically inert, nontoxic gas with large‐scale technical applications.[^10^, ^11^] Its utilization as cheap and safe fluorination reagent in chemical synthesis has therefore gathered considerable attention and becomes particularly desirable when it is combined with the disposal of the potent greenhouse gas after its technical use.^[12]^ Given the high oxidation state of sulfur, SF_6_ can act as strong oxidant and has the ability to transfer up to 6 fluorine atoms. However, the activation of SF_6_ under mild conditions requires a reduction step often leading to its complete decomposition, and thus the loss of its ability to act as an oxidant.^[13]^ The reductive activation of SF_6_ in the presence of suitable organic substrates is therefore an elegant way to perform fluorinations.^[14]^ Moreover, elaborated photocatalytic systems have even enabled introduction of entire SF_5_ groups into styrene derivatives.^[15]^ Despite these promising achievements, the in situ activation of SF_6_ by electron transfer has the drawback that the generated SF_6_⋅^−^ radical anion and its further fragmentation products are highly reactive, which can lead to unselective reactions. Moreover, considering the extreme global warming potential of SF_6_,[^12b^, ^12c^] its broad use as a reagent in excess should not disregard the need to avoid emissions. It is therefore desirable to obtain isolable well‐defined fluorination reagents from SF_6_. Successful approaches include the generation of rhodium SF_3_ complexes and of SF_5_^−^ salts using organic reductants, which have been employed for deoxyfluorinations.[^14d^, ^14e^, ^14f^, ^14h^, ^16^] Tlili and co‐workers also demonstrated that SF_5_^−^ salts can be reoxidized with TCICA to give SF_5_Cl, which was used for the chloro‐pentafluorosulfanylation of alkynes and alkenes.^[14i]^ We recently showed that the electron‐rich phosphine P(NI*i*Pr)_3_ (NI*i*Pr=1,3‐di‐*iso*‐propylimidazoline‐2‐ylidenamino) can monodefluorinate SF_6_ to afford the crystalline salt [(NI*i*Pr)_3_PF]^+^[SF_5_]^−^.^[17]^ Due to the inertness of the large phosphonium cation, the SF_5_^−^ salt is thermally stable up to a temperature of 140 °C. However, initial studies on the reaction with electrophiles showed that the phosphonium cation is fluorinated to the PF_6_^−^ anion by SF_4_, which is formed upon fluoride abstraction from the SF_5_^−^ anion. We herein show that by further increasing the chemical inertness of the phosphonium cation, the corresponding SF_5_^−^ salt can be used for the generation of reactive fluorinated selenium(IV) and tellurium(IV) anions and for the synthesis of novel fluorinated functional groups.

## Results and Discussion

**Figure 2:**
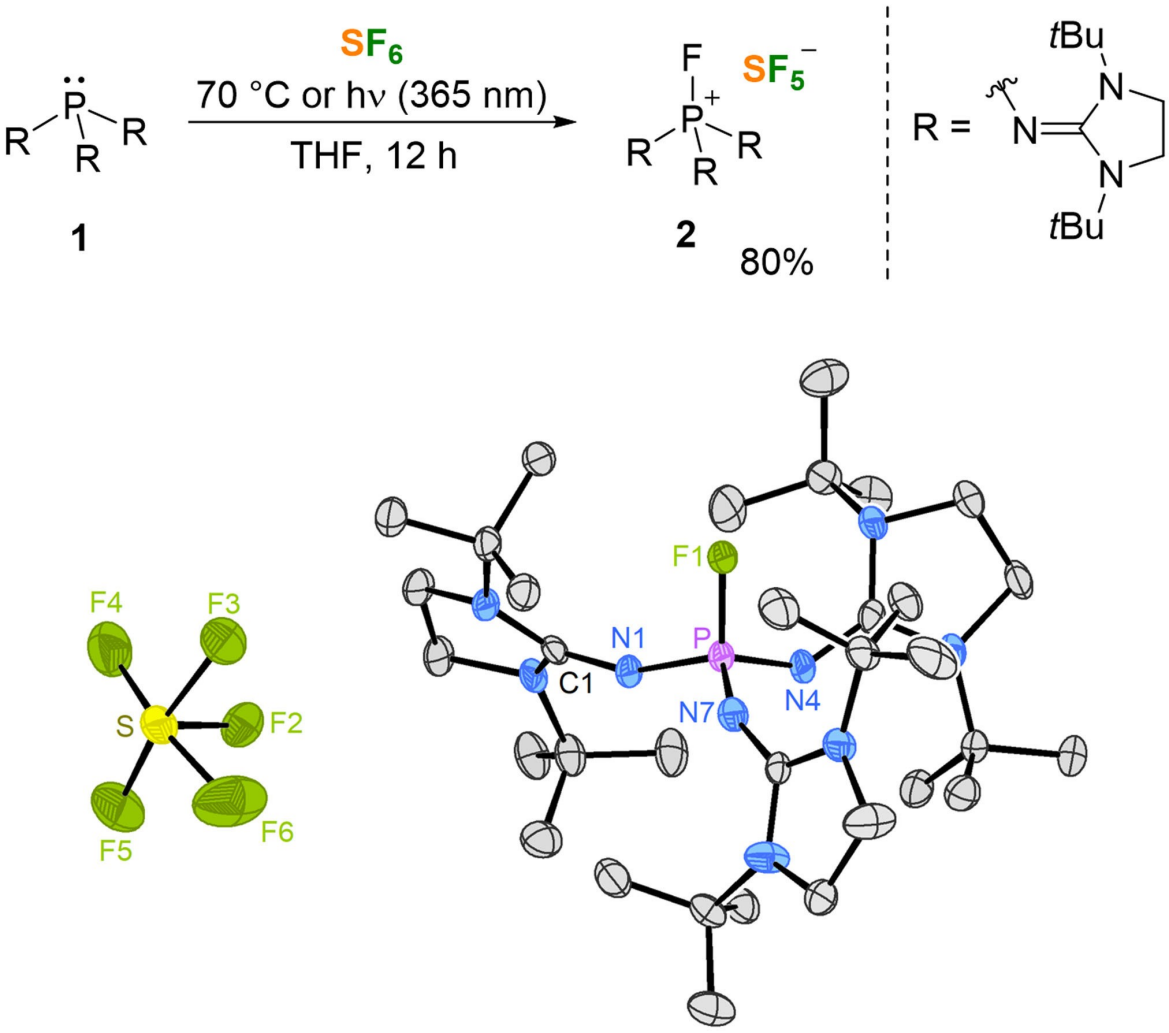
Top: Synthetic routes to the [SF_5_]^−^ salt **2** with isolated yield. Bottom: Molecular structure of **2**. Hydrogen atoms are omitted for clarity; thermal ellipsoids are set at 50 % probability. Selected bond lengths [Å] and angles [°]: P−N1 1.560(2), P−N4 1.592(2), P−N7 1.550(2), P−F1 1.577(2), S−F2 1.602(5), S−F3 1.774(5), S−F4 1.641(5), S−F5 1.662(5), S−F6 1.730(5); N1−P−F1 107.08(10), F1−P−N4 107.18(10), N7−P−F1 105.41(11), F2−S−F3 81.2(2), F2−S−F4 90.3(3), F2−S−F6 77.7(3), F2−S−F5 86.8(3), F4−S−F6 167.9(4), F5−S−F3 167.8(3).

Aiming at increasing the stability of the phosphonium cation towards SF_4_, we decided to use bulky *tert*‐butyl substituents at the imidazole nitrogen atoms, which should hamper coordination of the exocyclic nitrogen atoms to electrophiles. We recently showed that the sterically encumbered phosphine P(NsI*t*Bu)_3_ (**1**) (NsI*t*Bu=1,3‐di‐*tert*‐butylimidazolidine‐2‐ylidenamino) forms inert phosphonium cations after uptake of a chloronium ion and was therefore selected for the present study.^[18]^ Heating a THF solution of **1** under an atmosphere of SF_6_ to 70 °C for 12 hours led to the quantitative conversion to the salt [FP(NsI*t*Bu)_3_]^+^[SF_5_]^−^ (**2**) (Figure 2). Note that the reaction of the sterically encumbered phosphine **1** with SF_6_ is significantly slower than with P(NI*i*Pr)_3_, which immediately forms the corresponding SF_5_^−^ salt at −78 °C.^[17]^ The reaction between **1** and SF_6_ takes place at room temperature when the mixture is exposed to UV light (λ =365 nm). This accelerating effect has already been observed for the SF_6_ activation with other reducing agents.[^14b^, ^14d^, ^14e^, ^14i^] The SF_5_^−^ salt **2** is soluble in polar solvents such as CH_2_Cl_2_, MeCN and THF. A single‐crystal X‐ray diffraction (XRD) study^[19]^ reveals that the square pyramidal SF_5_^−^ anion forms no short contacts with the cation in the solid state. The pyramidalization of an imidazolidine N atom of the phosphonium ion reflects steric hindrance around the P atom and explains the harsher reaction conditions required for the synthesis of **2**. Solutions of **2** in dry THF can be heated to 160 °C for hours without appreciable decomposition, which is in contrast to the thermal sensitivity of other sulfur‐based fluorination reagents such as diethylaminosulfur trifluoride (DAST)^[20]^ or other SF_5_^−^ salts.[^14d^, ^16b^, ^21^]

**Scheme 1:**
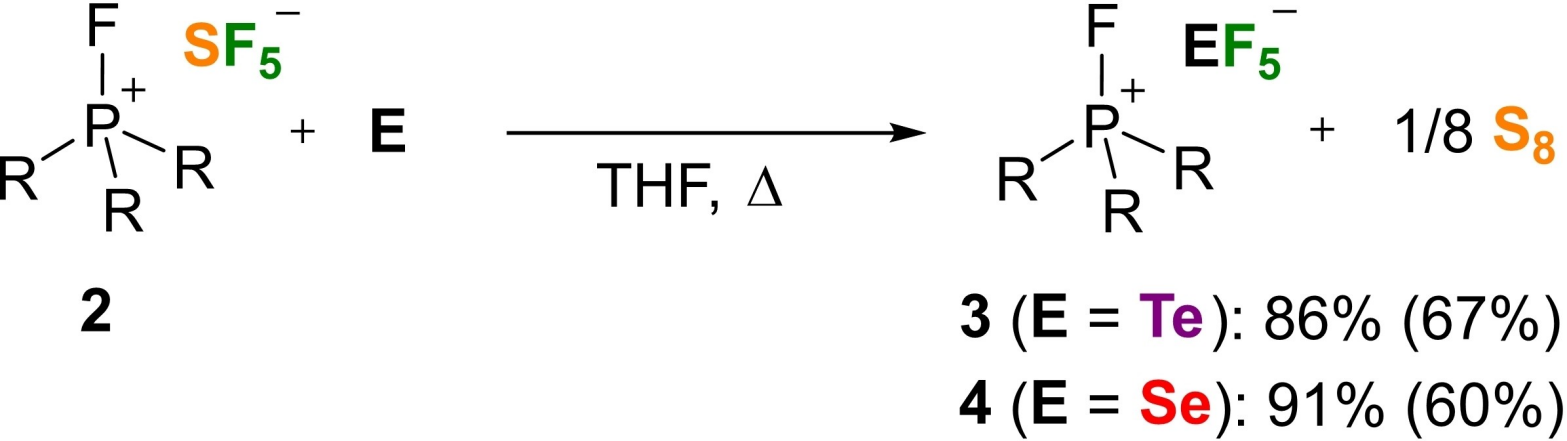
Fluorination of elemental tellurium and selenium using **2**. Conversion determined by quantitative ^19^F NMR spectroscopy; isolated yield in parenthesis. Reaction conditions for **3**: 80 °C, 72 h; **4**: 120 °C, 72 h. R=1,3‐di‐*tert*‐butylimidazolidin‐2‐ylidenamino.

Encouraged by the thermal stability of **2**, we studied the reaction of **2** with elemental selenium and tellurium. Heating a THF suspension of **2** and gray selenium or tellurium in a sealed glass vessel for three days gave the SeF_5_^−^ salt **4** and the TeF_5_^−^ salt **3** in 91 % and 86 % yield according to quantitative ^19^F NMR spectroscopy, respectively (Scheme 1). Note that SeF_5_^−^ and TeF_5_^−^ salts are usually prepared by fluorination of chalcogen(IV) oxides or tetrafluorides.^[22]^

The ^19^F NMR spectra show the characteristic resonances for an AX_4_ spin system of the TeF_5_^−^ and SeF_5_^−^ anions.[^4d^, ^22c^] A pentet and doublet are observed in the ^19^F NMR spectrum at −32.9 ppm and −38.0 ppm for TeF_5_^−^ and at 55.1 ppm and 17.4 ppm for SeF_5_^−^ with a coupling constant of ^2^ *J*_FF_=50.4 Hz (TeF_5_^−^) and ^2^ *J*_FF_=29.6 Hz (SeF_5_^−^), respectively. As a consequence of the complete fluorine transfer, elemental sulfur is formed as a side product. Indirect evidence for the stoichiometric formation of sulfur was provided by the addition of triphenyl phosphine to the product mixture affording SPPh_3_ and [FP(NsI*t*Bu)_3_]^+^ in a 1 : 1 ratio (see the Supporting Information, Chapter 1.6). After separation of sulfur, the salts **3** and **4** were isolated as white crystalline solids in 67 % and 60 % yield, respectively. The ^19^F NMR resonances of the isolated salt **4** are significantly broadened compared to those of the reaction mixture (see Figure S13 and S15). The same effect was observed for the SF_5_^−^ salt **2** (see Figure S3 and S5) as well as other SeF_5_^−^ containing salts.^[4d]^ To test the influence of the phosphonium cation, the oxidative fluorination of elemental selenium and tellurium was carried out under the same conditions using the less bulky salt [FP(NI*i*Pr)_3_]^+^[SF_5_]^−^,^[17]^ which gave the desired SeF_5_^−^ and TeF_5_^−^ anions in addition to significant amounts of PF_6_^−^ anions as a result of the partial degradation of the fluorophosphonium cation (see the Supporting Information, Chapter 1.12). As disclosed by these experiments, the SF_5_^−^ salt **2** combines two important properties: it can act as oxidative fluorinating reagent and can effectively stabilize fluorinated anions owing to the weakly coordinating nature of the chemically inert phosphonium ion.

**Figure 3:**
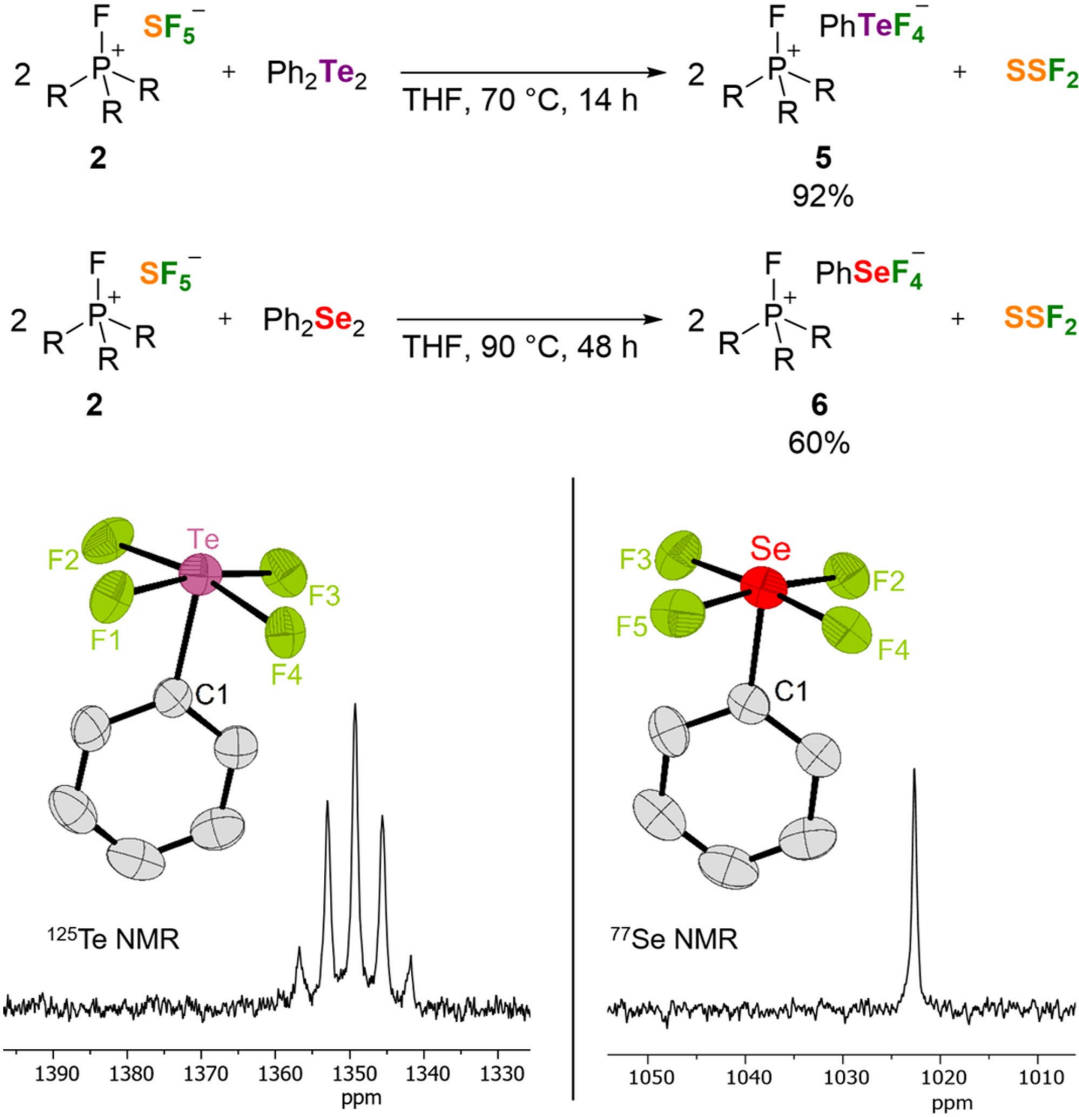
Top: Synthetic route to the [PhEF_4_]^−^ salts **5** and **6** (R=1,3‐di‐*tert*‐butylimidazolidin‐2‐ylidenamino). Bottom: Molecular structure of [PhTeF_4_]^−^ and [PhSeF_4_]^−^ together with the corresponding ^125^Te and ^77^Se NMR resonances. The fluorophosphonium cations and hydrogen atoms are omitted for clarity; thermal ellipsoids are set at 50 % probability. Selected bond lengths [Å] and angles [°]: **5**: Te−F1 1.997(3), Te−F2 1.994(3), Te−F3 1.993(3), Te−F4 1.997(3), Te−C1 2.109(5); F1−Te−F4 89.38(12), F1−Te−C1 84.9(2), F2−Te−F1 87.92(14), F2−Te−F4 168.07(13). **6**: Se−F2 1.877(3), Se−F3 1.897(4), Se−F4 1.872(4), Se−F5 1.885(4), Se−C1 1.956(6); F2−Se−F3 90.1(2), F2−Se−C1 86.7(2), F2−Se−F5 172.8(2).

To further explore this potential, we considered using **2** for the oxidative fluorination of organoselenium and organotellurium compounds. Common fluorination reagents such as XeF_2_,[^3a^, ^3g^] AgF_2_ ^[5a]^ or the TCICA/KF^[9]^ system convert diphenyl ditellurides and diphenyl diselenides into the neutral organofluorine species PhTe^VI^F_5_ and PhSe^IV^F_3_, respectively. By contrast, treatment of diphenyl ditelluride with **2** gave the salt [FP(NsI*t*Bu)_3_]^+^[PhTeF_4_]^−^ (**5**), which contains a novel tetrafluoro(phenyl)‐λ^5^‐tellurate anion, in addition to stochiometric amounts of thiothionyl fluoride (Figure 3). The reaction proceeds slowly at ambient temperature, but reaches full conversion within 14 hours at 70 °C. The formation of SSF_2_ was indicated by its characteristic resonance at 78.3 ppm in the ^19^F NMR spectrum of the reaction mixture (see the Supporting Information, Chapter 1.6). Further indication for the formation of SSF_2_ was obtained by the addition of Ph_3_P to the product mixture, which gave SPPh_3_ and Ph_3_PF_2_ next to [FP(NsI*t*Bu)_3_]^+^ in a 2 : 1 : 2 ratio as determined by ^31^P NMR spectroscopy. The [PhTeF_4_]^−^ salt **5** was isolated as a white crystalline solid in excellent yield. It shows good solubility in polar solvents such as MeCN and CH_2_Cl_2_ and moderate solubility in THF.

The [PhTeF_4_]^−^ anion completes the series of tetrahalo(phenyl)‐λ^5^‐tellurate anions [PhTeX_4_]^−^ (X=Cl, Br, I).^[23]^ It displays a singlet at −64.6 ppm in the ^19^F NMR spectrum and a pentet at 1349.3 ppm (^1^ *J*_TeF_=472 Hz) in the ^125^Te NMR spectrum (Figure 3). An XRD study reveals a square pyramidal coordination geometry of the tellurium atom with the phenyl group occupying the apical position. The equatorial Te−F bonds in **5** (1.993–1.997 Å) are slightly elongated compared to those of [TeF_5_]^−^ (1.923–1.973 Å).^[22c]^ While intermolecular tellurium‐halogen interactions have been detected in the solid‐state structures of [PhTeX_4_]^−^ (X=Cl, Br, I) salts, the structure of **5** is built up of isolated anions [PhTeF_4_]^−^ and cations [FP(NsI*t*Bu)_3_]^+^.

The reaction of **2** with diphenyl diselenide required more drastic conditions and could not be performed in standard borosilicate vessels. Heating a THF solution of **2** and diphenyl diselenide for 48 hours at 90 °C in a sealed PTFE vessel afforded [FP(NsI*t*Bu)_3_]^+^[PhSeF_4_]^−^ (**6**) after workup in 60 % yield. Crystals of **6**, suitable for an XRD analysis, were obtained by carefully layering a THF solution with diethyl ether. The selenium(IV) anion of **6** is isostructural to the tellurium congener in **5** (Figure 3). The Se−C1 bond (1.956 Å) and the Se−F bonds (1.872–1.885 Å) are in the same range as those in Mes_2_SeF_2_ (Se−C: 1.944 Å, Se−F: 1.876–1.887 Å).^[3f]^ Interestingly, to our knowledge **6** represents the first aryl tetrahalo‐λ^5^‐selenate anion of type [RSeX_4_]^−^. Similar square planar coordination of aryl selenium units can be found in the dimers of the neutral compounds RSeX_3_ (R=Aryl, X=F, Cl, Br), which exhibit intermolecular Se⋅⋅⋅X interactions in the solid state.[^5a^, ^24^] Compound **6** is a moisture sensitive, yellowish solid that is soluble in polar organic solvents. The [PhSeF_4_]^−^ salt shows a broad signal at −25.0 ppm in the ^19^F NMR spectrum and a singlet at 1022.7 ppm in the ^77^Se NMR spectrum suggesting a dynamic process. However, a variable temperature NMR experiment revealed only a broadening of the ^77^Se resonance at low temperature. To investigate the origin of this dynamic process, a second conformer with a fluoride at the apex of the square‐pyramidal structure was considered. However, its Gibbs free energy is 70 kJ mol^−1^ higher than that of the experimentally observed structure. The “concerted” isomerization has an energy barrier of Δ*G* ^≠^_298_=120 kJ mol^−1^ and proceeds via migration of a fluorine atom to the trans position of the phenyl group, leading to a significant elongation of the corresponding Se−F bond to 2.679 Å in the transition state structure (compare also Table S9). Fluoride dissociation, in the presence of an acceptor, and subsequent reassociation represent an alternative mechanism. Computational studies of the (hypothetical) dissociation of F^−^ from PhSeF_4_^−^ in the presence of HF to form PhSeF_3_ and FHF^−^ was found to be monotonously uphill in enthalpy. However, a low‐lying intermediate solely stabilized by entropic contributions was detected (Δ*G*_298_=−7 kJ mol^−1^). Calculations suggest that in this species F^−^ is dissociated from PhSeF_4_^−^ but due to the acceptor kept in close vicinity. Given the low energy differences, fast interconversion between the two complexes is possible (compare Table S9 Figure S75). Thus, fluoride dissociation/association mediated by an acceptor is the likely explanation for the observed dynamics in the NMR spectra of **6**.

To rank the stability of the obtained anions with respect to fluoride abstraction,^[26]^ we calculated the fluoride ion affinity (FIA) of the corresponding Lewis acids EF_4_ and PhEF_3_ using the method reported by Christe and co‐workers (Table 1).^[25]^ TeF_4_ has the highest FIA in this series,^[27]^ which is ranked between those of BF_3_ and PF_5_ and explains the low tendency of TeF_5_^−^ to react with water. Formal substitution of a fluorine atom by a phenyl group (PhTeF_3_) or of tellurium with selenium (SeF_4_) both decreases the FIA by about 50 kJ mol^−1^. In line with this trend, PhSeF_3_ displays the lowest electrophilicity in this series, which is consistent with the experimental observation that **6** reacts readily with the glass surface of the reaction vessel and decomposes quickly in the presence of moisture, whereas **5** does not react with the glass surface and decomposes slowly in the presence of water within several weeks (see Supporting Information, Chapter 1.11).

**Table 1:** Calculated fluoride ion affinities (FIA) of selected Lewis acids in the gas phase.

Lewis acid	FIA [kJ mol^−1^]^[a]^
PF_5_	373
TeF_4_	350
BF_3_	334
TeF_3_Ph	302
SeF_4_	298
SeF_3_Ph	256

[a] Determined by the method of Christe and co‐workers using COF_2_ as reference.^[25]^ Level of theory: wB97XD/aug‐cc‐pVTZ and aug‐cc‐pVTZ‐PP basis set for tellurium and selenium atoms.

**Figure 4:**
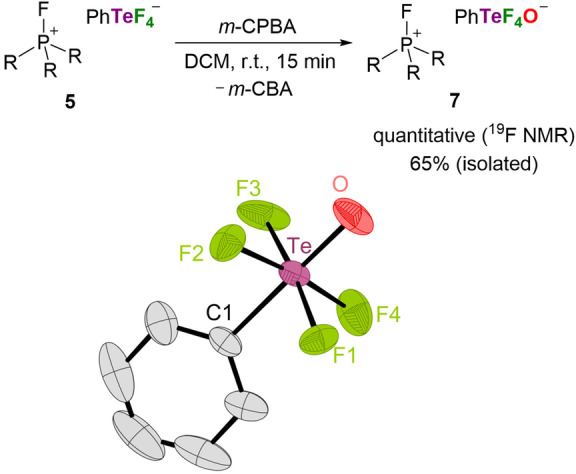
Top: Oxidation of **5** with *m*‐CPBA (R=1,3‐di‐*tert*‐butylimidazolidin‐2‐ylidenamino). Bottom: Molecular structure of [PhTeF_4_O]^−^ in **7**. Hydrogen atoms as well as the fluorophosphonium cation are omitted for clarity; thermal ellipsoids are set at 50 % probability. Selected bond lengths [Å] and angles [°]: Te−O 1.776(2), Te−F1 1.912(2), Te−F2 1.911(2), Te−F3 1.894(2), Te−F4 1.919(2), Te−C1 2.093(3); F1−Te−F4 88.89(10), F1−Te−C1 86.41(10), F2−Te−F1 89.71(9), F3−Te−F2 90.54(12), F3−Te−F4 90.09(12), O−Te−F1 92.75(11), O−Te−C1 179.15(12).

As the tellurium salt **5** is easy to isolate and rather stable in air, it appeared to be an interesting platform for further derivatizations of the new [PhTeF_4_]^−^ anion. We first attempted alkylation to access mixed aryl/alkyl tetrafluoro‐λ^6^‐organotellurium compounds. These species are poorly studied and have been prepared by fluorination of organotellanes R_2_Te.[^3a^, ^9^] However, the [PhTeF_4_]^−^ anion turned out to be a very sluggish nucleophile, which did not react with allyl iodide at temperatures up to 100 °C. Using methyl triflate or (Et_3_O)BF_4_, slow decomposition of **5** was observed at room temperature under formation of PF_6_^−^ and alkyl fluorides (see Supporting Information, Chapter 1.10). An XRD study of a crystal grown from the reaction mixture revealed a dicationic Te^IV^ compound **11** generated by partial degradation of the phosphonium ion (see Supporting Information, Chapter 1.10 and 2.5). We proceeded in our efforts in further derivatization by reducing the fluoride donor ability of the [PhTeF_4_]^−^ anion via oxidation with 3‐chloroperbenzoic acid (*m*‐CPBA). The [PhTeF_4_]^−^ salt **5** was cleanly oxidized to the [PhTeF_4_O]^−^ salt **7** using *m*‐CPBA (Figure 4). Compound **7** was isolated in 65 % yield due to the challenging separation of chlorobenzoic acid. The ^19^F resonance of [PhTeF_4_O]^−^ is detected as a singlet at −35.2 ppm. The ^125^Te NMR spectrum shows a pentet at 697.0 ppm with a coupling constant of ^1^ *J*_TeF_=3230.6 Hz.

The *trans* arrangement of the phenyl and oxido substituents was indicated by the NMR data and confirmed by an XRD study (Figure 4). The [PhTeF_4_O]^−^ anion adopts an octahedral geometry with a slightly shorter Te−O bond (1.776 Å) and longer Te−F bonds (1.894–1.919 Å) than those of the “teflate” anion [TeF_5_O]^−^ (Te−O: 1.786 Å, Te−F: 1.846–1.862 Å).^[28]^ Note that the attempted oxidation of the [PhSeF_4_]^−^ anion with *m*‐CPBA was unselective presumably due to the deoxyfluorination of 3‐chlorobenzoic acid by **6** (see Supporting Information, Chapter 1.8).

**Scheme 2:**
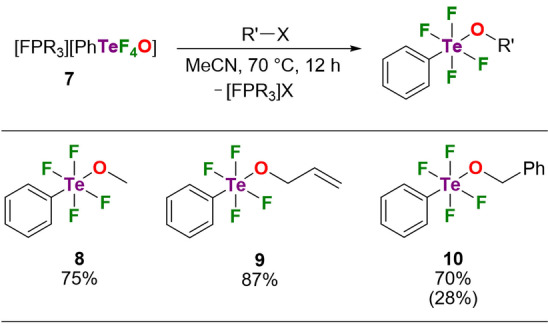
Alkylation of [PhTeF_4_O]^−^ (R=1,3‐di‐*tert*‐butylimidazolidin‐2‐ylidenamino). Yields were determined by ^19^F NMR spectroscopy. Isolated yield for **10** in parenthesis.

Gratifyingly, the new [PhTeF_4_O]^−^ anion can be alkylated by common reagents such as methyl iodide, allyl iodide and benzyl bromide to afford *trans*‐alkoxytetrafluoro(phenyl)‐λ^6^‐telluranes **8**–**10** in good yield (Scheme 2). The phenyl and alkoxy substituents adopt *trans* positions as disclosed by the singlet in the ^19^F NMR spectra and the pentet resonance in the ^125^Te NMR spectra. The axial ^1^ *J*_TeF_ coupling constants of tetrafluorotellurium(VI) species increase in the order [*trans*‐PhTeF_4_O]^−^ (**7**: 3231 Hz), *trans*‐PhTeF_4_OR (**8**–**10**: 3526–3548 Hz), [TeF_5_O]^−^ (3643 Hz),^[28]^ *trans*‐(MeO)_2_TeF_4_ (3672 Hz),^[29]^ indicating an increasing electrophilicity of the Te center. In fact, the latter can accommodate a fifth fluoride anion, forming the pentagonal bipyramidal anion [*trans*‐(MeO)_2_TeF_5_]^−^.^[29]^ It is noteworthy that very little is known about the TeF_4_(OR) group on arenes and the *trans* isomers are unknown. This is likely due to the fact that the reaction of PhTeF_5_ with an excess of methanol or water selectively produces *cis*‐PhTeF_4_(OR) (R=H, Me).[^9^, ^30^] According to our calculations the *cis* and the *trans* isomer of PhTeF_4_(OMe) are very similar in energy, with DLPNO‐CCSD(T)/def2‐TZVP calculations in THF slightly favoring the *cis* isomer by −0.8 kJ mol^−1^ in free energy. This difference might very well be within the error margin of the calculations, in particular since the relativistic effects of the tellurium atom are only considered via effective core potentials and not explicitly included in the calculations. Further exchange of fluorine atoms with OH groups in *cis*‐PhTeF_4_(OH) was shown to happen much slower.^[9]^ Consistent with this stability trend, compounds **8**–**10** can be handled in air with no observable degradation. A hydrolysis experiment showed that **10** undergoes very slow exchange of fluorine atoms for OH groups (less than 1 % after 3 hours) when dissolved in a 10 : 1 mixture of acetonitrile and water, forming the putative hydrolysis product PhTeF_3_(OH)(OCH_2_Ph).

## Conclusion

In conclusion, a new oxidative fluorination reagent (**2**) consisting of an SF_5_^−^ anion and an inert phosphonium cation is reported. Compared to other SF_5_^−^ salts, **2** exhibits exceptional thermal stability, making it a powerful reagent for the generation and stabilization of reactive fluorinated anions. For example, **2** was used for the fluorination of elemental selenium and tellurium to the corresponding SeF_5_^−^ and TeF_5_^−^ salts. We also synthesized and characterized the new anions PhSeF_4_^−^, PhTeF_4_^−^, and *trans*‐PhTeF_4_O^−^ from the corresponding diphenyl dichalcogenides. Alkylation of *trans*‐PhTeF_4_O^−^ leads to the first *trans*‐(alkoxy)(phenyl)tetrafluoro‐λ^6^‐tellanes (*trans*‐PhTeF_4_OR), demonstrating a way to introduce the functional group into organic molecules. Given the stability of the *trans*‐PhTeF_4_OR motif, it could become an alternative candidate for the arsenal of lipophilic electron‐withdrawing groups^[31]^ such as the SF_5_ group,[^1^, ^2^, ^32^] which is relevant for medicinal chemistry^[33]^ and materials applications.^[34]^

## Conflict of interest

The authors declare no conflict of interest.

1

## Supporting information

As a service to our authors and readers, this journal provides supporting information supplied by the authors. Such materials are peer reviewed and may be re‐organized for online delivery, but are not copy‐edited or typeset. Technical support issues arising from supporting information (other than missing files) should be addressed to the authors.

Supporting Information

Supporting Information

Supporting Information

Supporting Information

Supporting Information

Supporting Information

## Data Availability

The data that support the findings of this study are available in the supplementary material of this article.
