# Supplementary material for: Oxidative Fluorination of Selenium and Tellurium Compounds using a Thermally Stable Phosphonium SF5 − Salt Accessible from SF6
Source: Angew Chem Int Ed Engl. 2022 Sep 12;61(42):e202209067. doi: 10.1002/anie.202209067 (PMC9826459; doi:10.1002/anie.202209067)
Supplement: Supplementary file 6 — Supporting Information [file ANIE-61-0-s001.pdf]

Angewandte Chemie
Eine Zeitschrift der Gesellschaft Deutscher Chemiker

Supporting Information

Oxidative Fluorination of Selenium and Tellurium Compounds using a Thermally Stable Phosphonium SF_5^- Salt Accessible from SF_6

*T. Eder, F. Buß, L. F. B. Wilm, M. Seidl, M. Podewitz, F. Dielmann**

Supplementary Material for

Oxidative Fluorination of Selenium and Tellurium Compounds using a Thermally Stable Phosphonium SF_5^- Salt Accessible from SF_6

Tobias Eder,[†#] Florenz Buß,[#] Lukas F.B. Wilm,[#] Michael Seidl,[†] Maren Podewitz,[‡] Fabian Dielmann[†]*

† Institute of General and Theoretical Chemistry
Leopold-Franzens Universität Innsbruck
Innrain 80-82, 6020 Innsbruck (Austria)

Institute of Inorganic and Analytical Chemistry
Westfälische Wilhelms-Universität Münster
Corrensstrasse 28-30, 48149 Münster (Germany)

‡ Institute of Materials Chemistry
TU Wien
Getreidemarkt 9, 1060 Vienna (Austria)

E-mail: fabian.dielmann@uibk.ac.at

CONTENTS:

1 EXPERIMENTAL PROCEDURES **3**

1.1 Synthetic Details 3

1.2 Synthesis of the SF_5^- salt **2** 4

1.3 Synthesis of compounds **3** and **4** 7

1.4 Preparation of **5** 11

1.5 Preparation of **6** 13

1.6 Trapping experiments to identify the sulfur species formed in the synthesis of **3** and **5** using triphenylphosphine 16

1.7 Synthesis of **7** by oxidation of **5** ($PhTeF_4$) with *m*-CPBA 19

1.8 Attempts of oxidizing **6** with *m*-CPBA Synthesis 22

1.9 Synthesis of *trans*-alkoxytetrafluoro(phenyl)-λ^6-telluranes **8-10** 23

1.9.1. Preparation of **10** 24

1.9.2. Preparation of **8** 29

1.9.3. Preparation of **9** 30

1.10 Reaction of $[FP(NsItBu)_3][PhTeF_4]$ (**5**) with alkylation reagents 33

1.11 Hydrolysis experiments of $[FP(NsItBu)_3][PhTeF_4]$ (**5**) and *trans*-$PhTeF_4(OMe)$ (**10**) 35

1.12 Oxidative fluorination of elemental selenium and tellurium using $[FP(NI\textit{i}Pr)_3][SF_5]$ (NI*i*Pr = 1,3-diisopropyl-4,5-dimethylimidazolin-2-ylidenamino) 37

2 X-RAY DIFFRACTION STUDIES **39**

2.1 Crystal structure data of compound **2** 40

2.2 Crystal structure data of compound **5** 41

2.3 Crystal structure data of compound **6** 42

2.4 Crystal structure data of compound **7** 43

2.5 Crystal structure data of compound **11** 44

3 COMPUTATIONAL DATA **46**

REFERENCES **55**

1 Experimental procedures

1.1 Synthetic Details

General remarks: Unless noted otherwise, all manipulations were performed under an inert atmosphere of dry argon, using standard Schlenk and drybox techniques. Dry and oxygen-free solvents were employed. All glassware was oven-dried at 160 °C prior to use. Silica used for flash column chromatography was dried at 150 °C for 3 days. Chemical shifts are given in parts per million (ppm) relative to $SiMe_4$ (^1H, ^{13}C, ^{29}Si), 85% H_3PO_4 (^{31}P), CCl_3F (^{19}F), Me_2Se (^{77}Se), 90% Me_2Te in C_6D_6 (^{125}Te) and were referenced to the residual solvent signals (C_6D_6: ^1H $\delta_H = 7.16$, ^{13}C $\delta_C = 128.06$; CD_3CN: $\delta_H = 1.94$, ^{13}C $\delta_C = 118.26$; $CDCl_3$: $\delta_H = 7.26$, ^{13}C $\delta_C = 77.16$; CD_2Cl_2: $\delta_H = 5.32$, ^{13}C $\delta_C = 53.84$; THF-d_8: ^1H $\delta_H = 1.72$, ^{13}C $\delta_C = 67.21$) or internally by the instrument after locking and shimming to the deuterated solvent (^{19}F, ^{31}P). NMR multiplicities are abbreviated as follows: s = singlet, d = doublet, t = triplet, p = pentet, sept = septet, m = multiplet, br = broad signal. Mass spectra were recorded using an Orbitrap LTQ XL (Thermo Scientific) spectrometer and an Orbitrap QExactive (Thermo Scientific) spectrometer. Elemental analyses were determined by the microanalytic laboratory of the Westfälische Wilhelms-Universität Münster. IR spectra were recorded using a Bruker ALPHA II ATR-FTIR spectrometer. Irradiation experiments were performed with a EvoluChem™ LED Typ P205-18-1 (365 nm).
Safety remarks: *The use of a burst shield is strongly recommended for all reactions carried out in closed Schlenk flasks under pressurized gas or when heated above the boiling point of the respective solvent!* Caution should be exercised when drying 3-chloroperoxybenzoic acid (*m*-CPBA) as it is shock sensitive in dried form. Therefore, it is recommended to use only small amounts, not to heat the sample during the drying process and to dissolve the dried *m*-CPBA in a suitable solvent.
Reagents and Handling: Phosphine **1** and the fluorophosphonium salt $[FP(NIiPr)_3][SF_5]$ were synthesized according to literature procedures.[1,2] All other compounds were purchased from commercial sources (Sigma Aldrich, Alfa Aesar, abcr GmbH, Tokyo Chemical Industry). The amount of active 3-chloroperoxybenzoic acid (*m*-CPBA) in the purchased batch has been determined by iodometric titration[3] and the *m*-CPBA was then carefully dried *in vacuo*, dissolved in DCM and stored at 7 °C. Diphenyl diselenide was recrystallized from hexane and triphenyl phosphine was sublimated before use. All other chemicals were used as received.

1.2 Synthesis of the SF_5^- salt 2

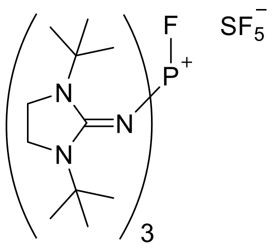

The SF_5^- salt **2** was prepared either by heating (70 °C) or by irradiation (with light at 365 nm) of a solution of phosphine **1** (600 mg, 0.968 mmol) in THF (3 mL) under 2 bar of SF_6 gas in a sealed Schlenk flask. After 12 hours, a crystalline material had been formed which was isolated by filtration and contained pure **2** as indicated by NMR analysis. The volume of the mother liquor was then reduced to 1 mL and diethyl ether (7 mL) was added. The formed precipitate was collected, washed with diethyl ether, until the supernatant solution turned colorless (5 × 5 mL), and dried *in vacuo* to afford **2** as a white crystalline solid.

Table S1: Reaction conditions and isolated yield of **2**.

	wave length	*temperature*	*reaction time*	*isolated yield of* ***2***
**1** + SF_6 (2 bar)	none	70 °C	12 hours	77%
	365 nm	ambient temperature	12 hours	80%

Note that small amounts of $[FHF]^-$ impurities that might be present in the product due to hydrolysis can be separated by recrystallization from hot THF. Storing the saturated solution at -40 °C affords the pure SF_5^- salt **2**. In fact, crystalline **2** is poorly soluble in THF, but impurities of $[FHF]^-$ anions increase the solubility significantly.

^{1}H NMR (500 MHz, 300 K, THF-d_8): δ = 3.44 (s, 12 H, CH_2), 1.41 (s, 54 H, CH_3).

^{13}C{^{1}H} NMR (126 MHz, 300 K, THF-d_8): δ = 152.3 (dd, $^2J_{CP}$ = 34.1 Hz, $^3J_{CF}$ = 2.3 Hz, *C*=N), 56.0 (*C*(CH_3)$_3$), 43.2 (*C*H_2), 28.8 (C(*C*H_3)$_3$).

^{19}F NMR (471 MHz, 300 K, THF-d_8): δ = 88.8 (p, $^2J_{FF}$ = 45.8 Hz), 59.1 (d, $^2J_{FF}$ = 45.8 Hz), –36.4 (d, $^1J_{PF}$ = 910.7 Hz).

Note: The ^{19}F NMR resonances of the pure $[SF_5]^-$ salt **2** in THF are broadened (Figure S3) while the SF_5^- anion shows sharp signals in the reaction mixture (Figure S5).

^{31}P NMR (202 MHz, 300 K, THF-d_8): δ = –60.0 ppm (d, $^1J_{PF}$ = 910.7 Hz).

HRMS (ESI, positive): m/z calc. for $[C_{33}H_{66}N_9FP]^+$ 638.5157, found 638.5140.

Note: The $[SF_5]^-$ anion was not detected in the negative HRMS-ESI Mode.

Elemental analysis: calculated (%) for $C_{33}H_{66}F_6N_9PS$: C 51.75, H 8.69, N 16.46, found C 52.33, H 8.94, N 16.62.

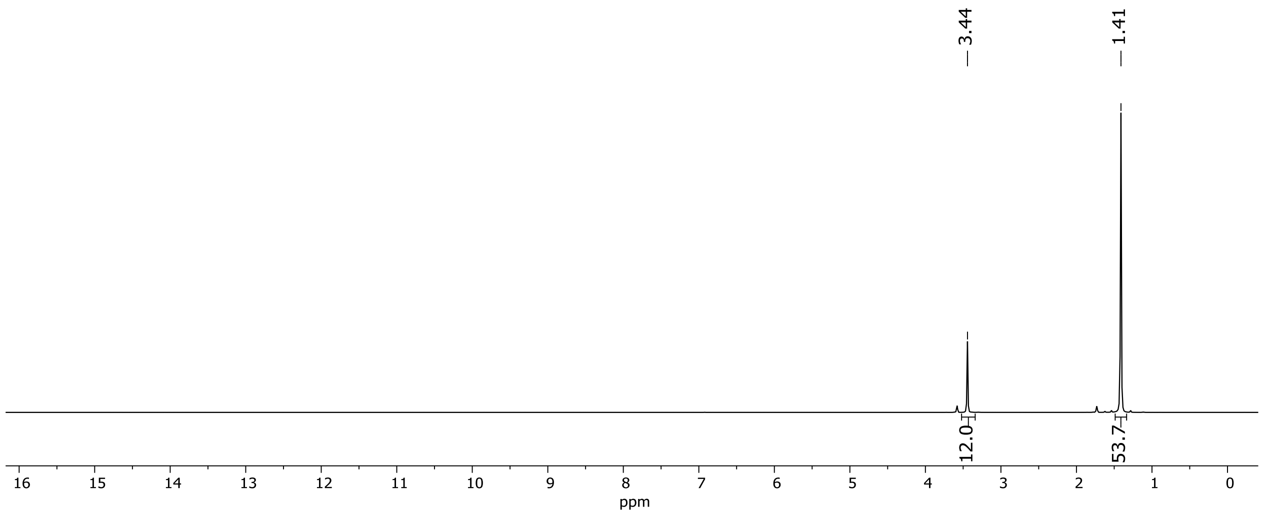

Figure S1: ^{1}H NMR spectrum (THF-d_8, 500 MHz) of **2**.

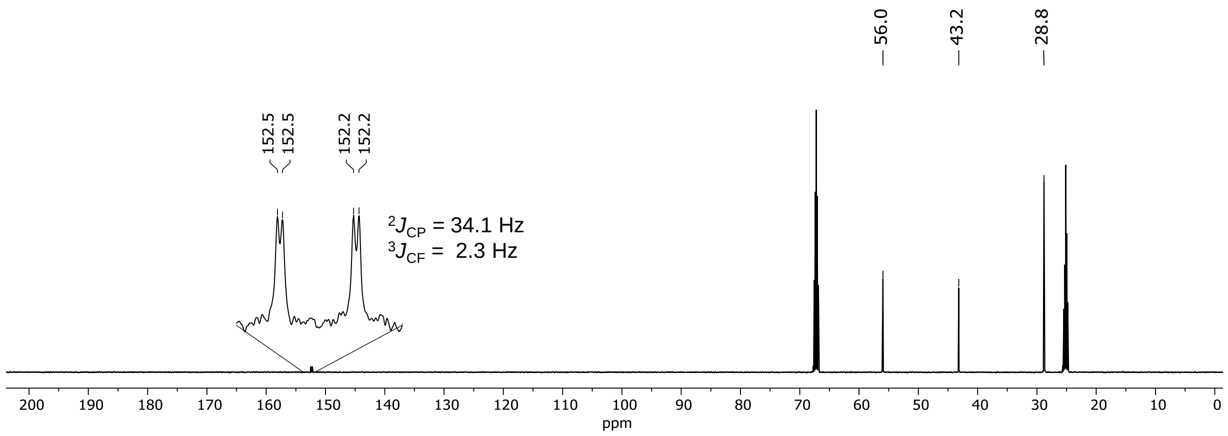

Figure S2: $^{13}C\{^{1}H\}$ NMR spectrum (THF-d_8, 126 MHz) of **2**.

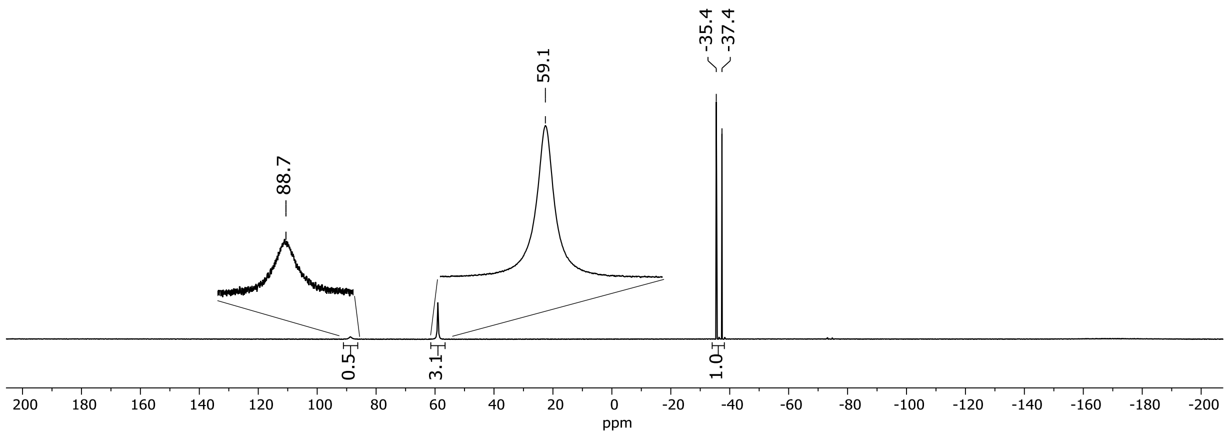

Figure S3: ^{19}F NMR spectrum (THF-d_8, 471 MHz, D_1 = 60 sec, aq. time = 0.44 sec., zg) of **2**.

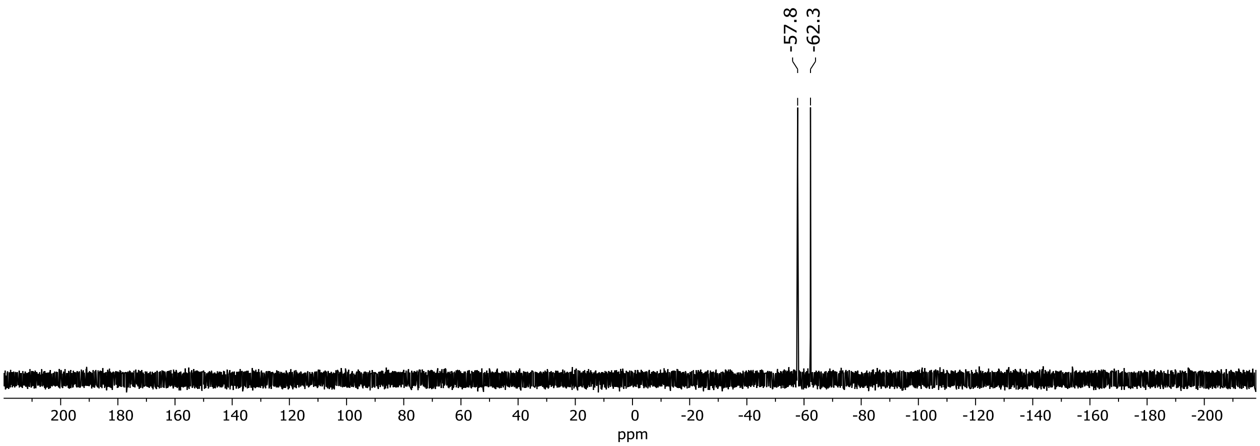

Figure S4: ^{31}P NMR spectrum (THF-d_8, 202 MHz) of **2**.

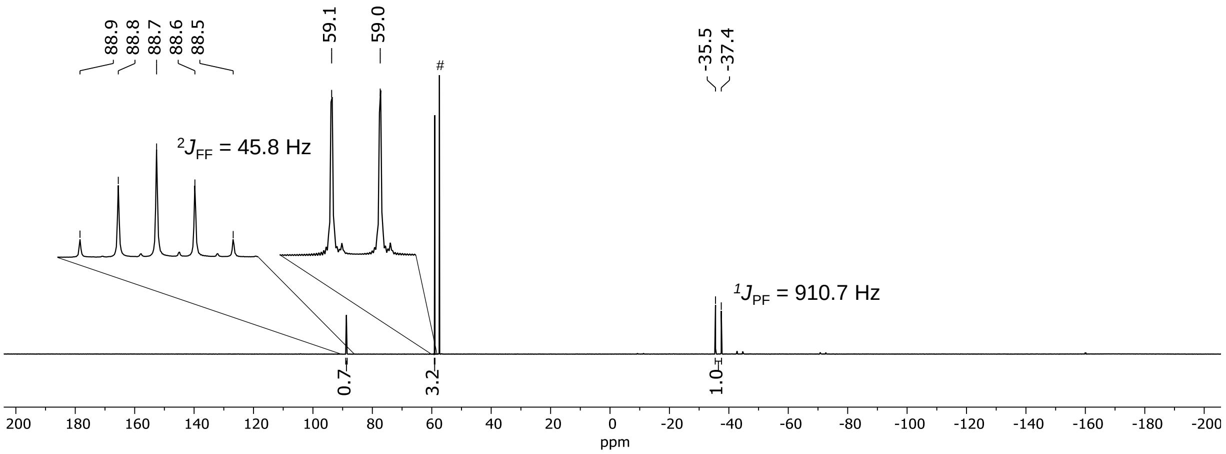

Figure S5: ^{19}F NMR spectrum (THF-d_8, 471 MHz, D_1 = 45 sec, aq. time = 0.44 sec., zg) of the reaction mixture of the reaction of phosphine **1** with SF_6 after 2 hours at 70 °C. **#** Signal of SF_6.

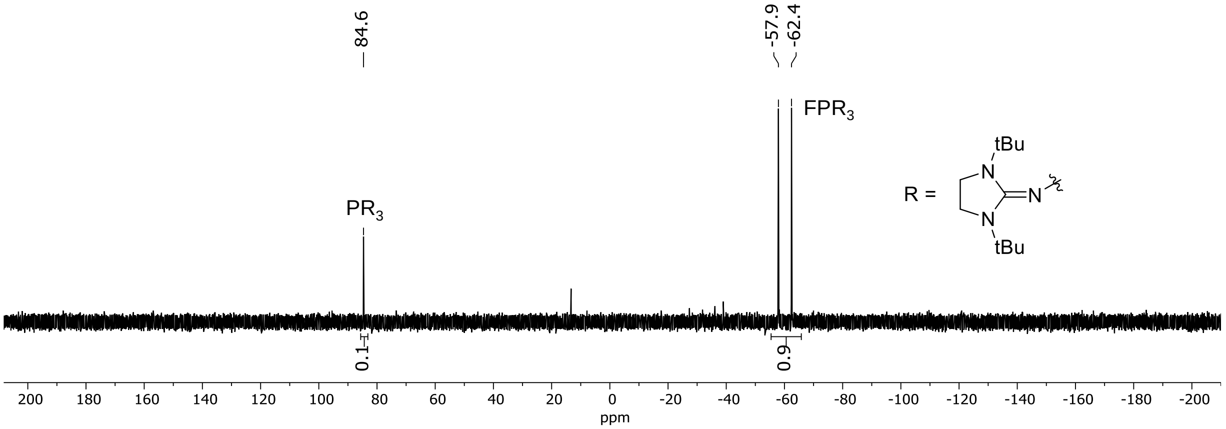

Figure S6: ^{31}P NMR spectrum (THF-d_8, 202 MHz, D_1 = 60 sec., aq. time = 0.81 sec., zg30) of the reaction mixture of the reaction of phosphine **1** with SF_6 after 2 hours at 70 °C.

1.3 Synthesis of compounds 3 and 4

General procedure: Phosphonium salt **2** and powdered gray selenium or tellurium were suspended in THF (5 mL). The mixture was stirred at the indicated temperature and for the indicated time in a sealed Schlenk flask. The conversion of **2** was monitored by quantitative ^{19}F NMR spectroscopy based on the fluorophosphonium cation $[FPR_3]^+$ as internal standard. After cooling the mixture to ambient temperature, the excess of tellurium or selenium was filtered off and the volume of the filtrate was reduced to 1 ml. The product was precipitated by slow diffusion of diethyl ether into the solution. The crystalline material was separated by filtration, washed with diethyl ether, and dried *in vacuo* at 60 °C for two hours.

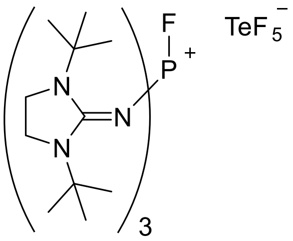

Compound 3 was prepared from **2** (50 mg, 0.065 mmol, 1.0 eq.) and tellurium (10 mg, 0.078 mmol, 1.2 eq.) following the general procedure by heating at 80 °C for 72 h. Compound **3** was isolated as a white, crystalline solid.

Yield according to quantitative ^{19}F NMR analysis (THF): 86%.

Isolated yield: 67% (37 mg, 0.043 mmol).

^{1}H NMR (500 MHz, 300 K, THF-d_8): δ = 3.43 (s, 12 H, CH_2), 1.40 (s, 54 H, CH_3).

^{13}C{^{1}H} NMR (126 MHz, 300 K, THF-d_8): δ = 152.3 (dd, $^2J_{CP}$ = 34.0 Hz, $^3J_{CF}$ = 2.4 Hz, *C*=N), 56.0 (*C*(CH_3)$_3$), 43.2 (*C*H_2), 28.8 (C(*C*H_3)$_3$).

^{19}F NMR (471 MHz, 300 K, THF-d_8): δ = –32.9 (p, $^2J_{FF}$ = 50.3 Hz, $^1J_{125TeF}$ = 2981.3 Hz, Te–F$_{ax}$), –36.4 (d, $^2J_{PF}$ = 910.0 Hz, P–F), –38.0 (d, $^2J_{FF}$ = 50.3 Hz, $^1J_{125TeF}$ = 1467.2 Hz Te–F$_{eq}$).

^{31}P NMR (202 MHz, 300 K, THF-d_8): δ = –60.4 (d, $^1J_{PF}$ = 910.2 Hz).

HRMS (ESI, positive): m/z calc. for $[C_{33}H_{66}N_9FP]^+$ 638.51573, found 638.52040.

HRMS (ESI, negative): m/z calc. for $[TeF_5]^-$ 224.89879, found 224.91667.

Elemental analysis: calculated (%) for $C_{33}H_{66}F_6N_9PTe$: C 46.01, H 7.72, N 14.63, found C 46.42, H 7.53, N 14.77.

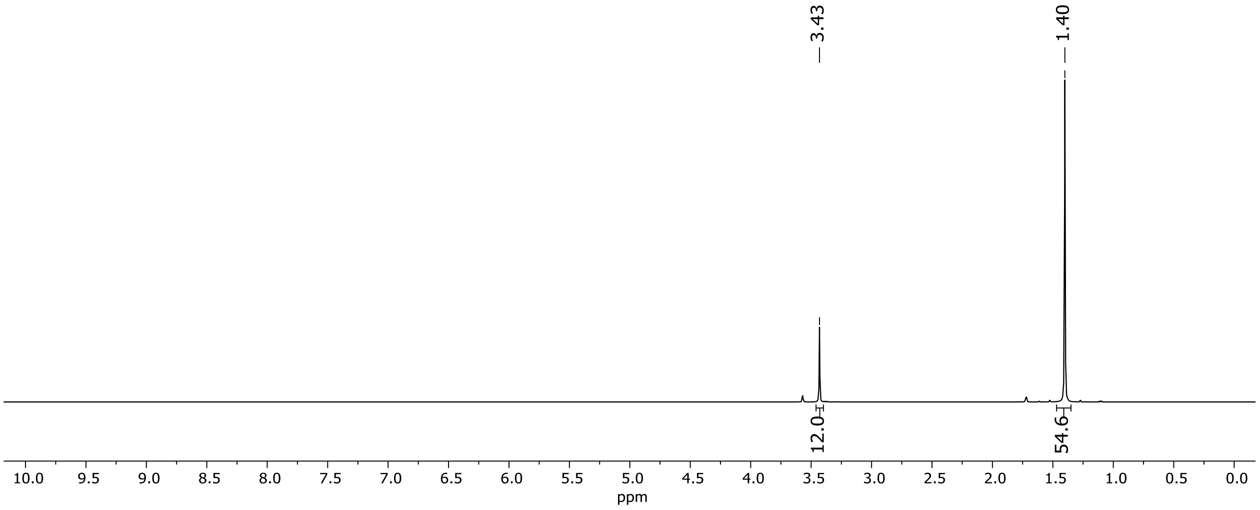

Figure S7: ^{1}H NMR spectrum (THF-d_8, 500 MHz) of **3**.

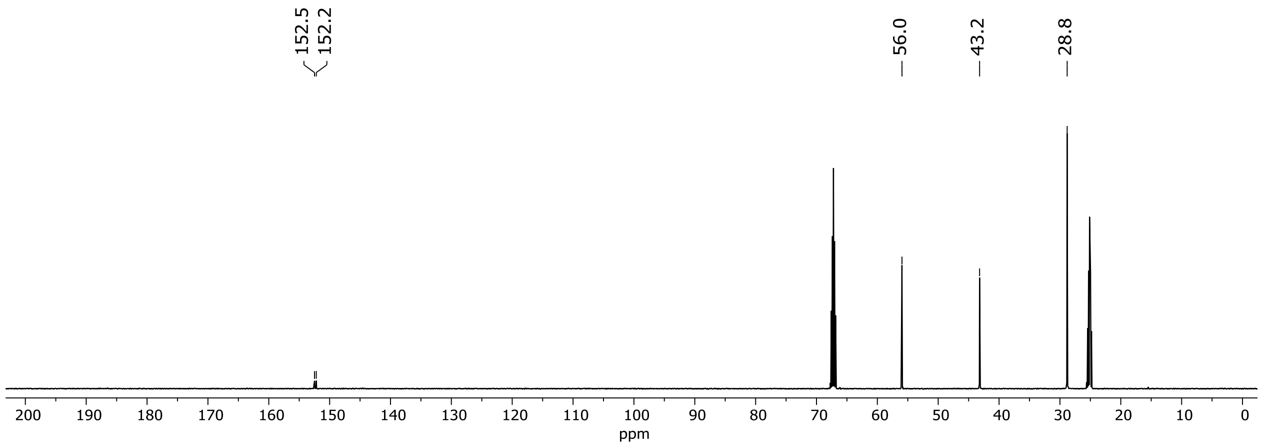

Figure S8: $^{13}C\{^{1}H\}$ NMR spectrum (THF-d_8, 126 MHz) of **3**.

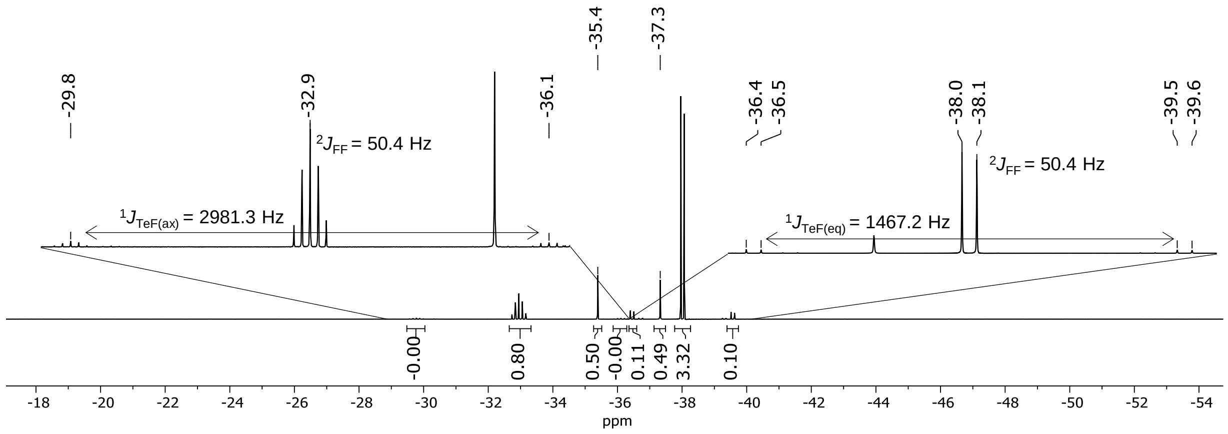

Figure S9: ^{19}F NMR spectrum (THF-d_8, 471 MHz, D_1 = 60 sec, aq. time = 0.44 sec., zg) of **3**.

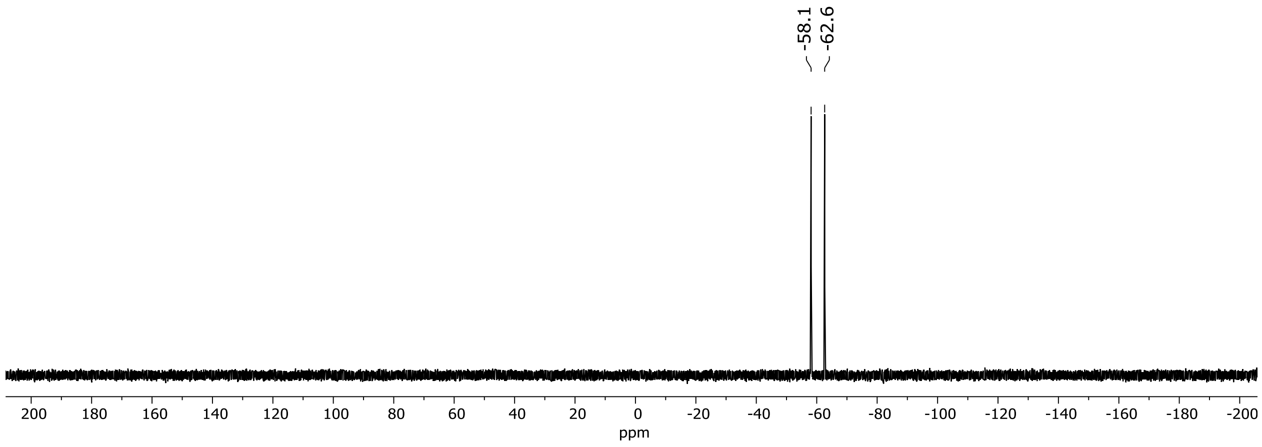

Figure S10: ^{31}P NMR spectrum (THF-d_8, 202 MHz) of **3**.

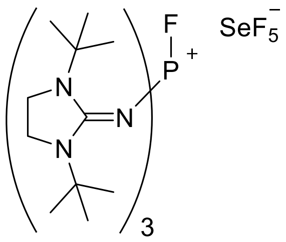

Compound 4 was prepared from **2** (50 mg, 0.065 mmol, 1.0 eq.) and selenium (8 mg, 0.101 mmol, 1.56 eq.) following the general procedure by heating at 120 °C for 72 h. Compound **4** was isolated as a white, crystalline solid.

Note: Burst shield is strongly recommended.

Yield according to quantitative ^{19}F NMR analysis (THF): 91%.

Isolated yield: 60% (32 mg, 0.043 mmol).

^{1}H NMR (400 MHz, 300 K, THF-d_8): δ = 3.43 (s, 12 H, CH_2), 1.40 (s, 54 H, CH_3).

^{13}C{^{1}H} NMR (101 MHz, 300 K, THF-d_8): δ = 152.3 (dd, $^{2}J_{CP}$ = 34.0 Hz, $^{3}J_{CF}$ = 2.4 Hz, *C*=N), 56.0 (*C*(CH_3)$_3$), 43.2 (*C*H_2), 28.8 (C(*C*H_3)$_3$).

^{19}F NMR (376 MHz, 300 K, THF-d_8): δ = 55.1 (p, $^{2}J_{FF}$ = 29.6 Hz, $^{1}J_{SeF}$ = 1159 Hz), 17.4 (d, $^{2}J_{FF}$ = 29.6 Hz, $^{1}J_{SeF}$ = 195 Hz), –36.8 (d, $^{1}J_{PF}$ = 911 Hz).

Note: The ^{19}F NMR resonances of the pure SeF_5^- salt **4** in THF are broadened (Figure S13) while the SeF_5^- anion shows sharp signals in the reaction mixture (Figure S15).

^{31}P NMR (162 MHz, 300 K, THF-d_8): δ = –60.4 (d, $^{1}J_{PF}$ = 910.2 Hz).

HRMS (ESI, positive): m/z calc. for $[C_{33}H_{66}N_9FP]^+$ 638.51573, found 638.51885.

Note: The SeF_5^- anion was not detected in the negative HRMS-ESI Mode.

Elemental analysis: calculated (%) for $C_{33}H_{66}F_6N_9PSe$: C 48.76, H 8.18, N 15.51, found C 49.47, H 8.21, N 15.61.

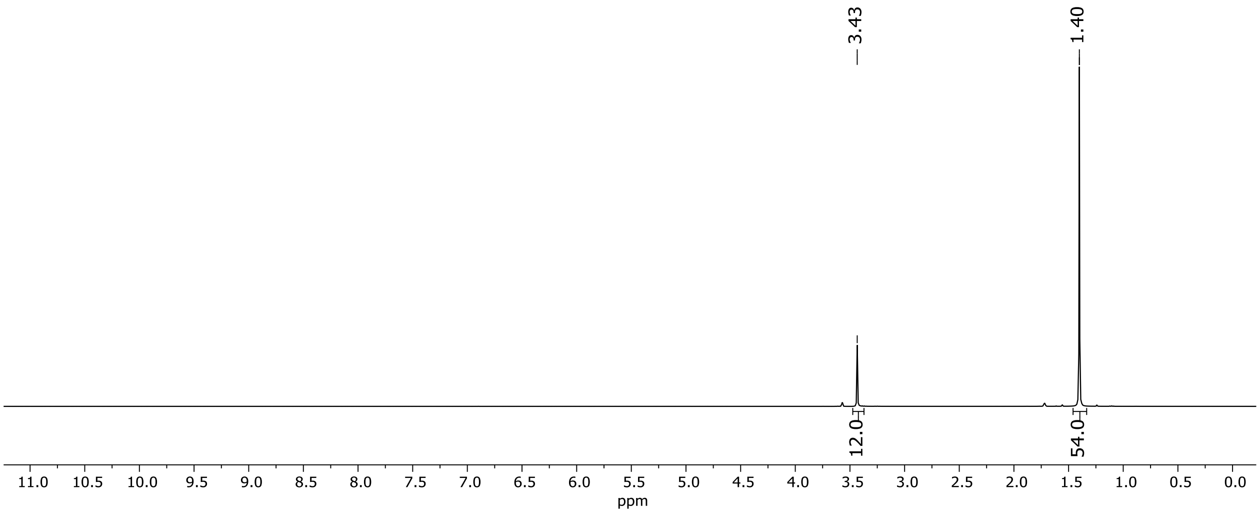

Figure S11: ^{1}H NMR spectrum (THF-d_8, 400 MHz) of **4**.

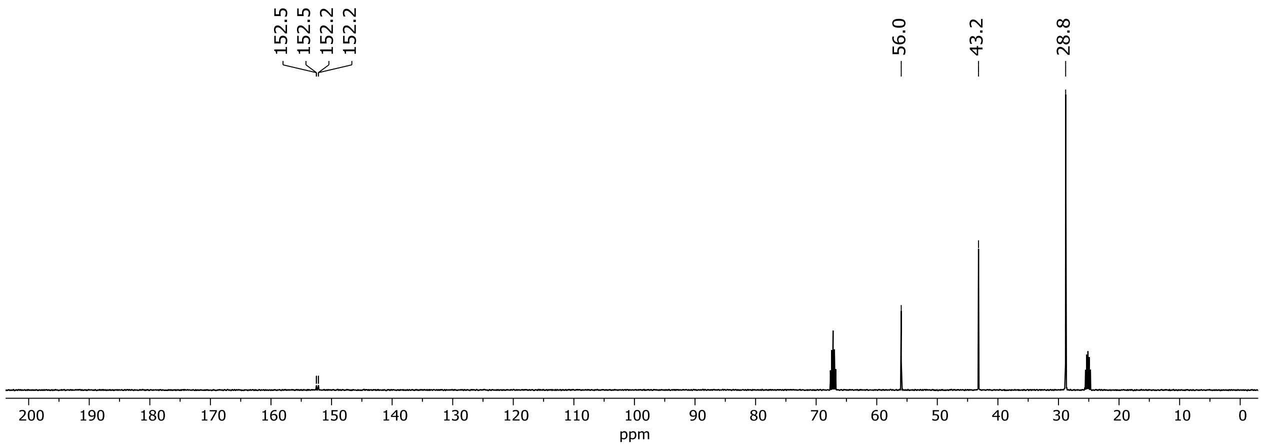

Figure S12: $^{13}C\{^{1}H\}$ NMR spectrum (THF-d_8, 101 MHz) of **4**.

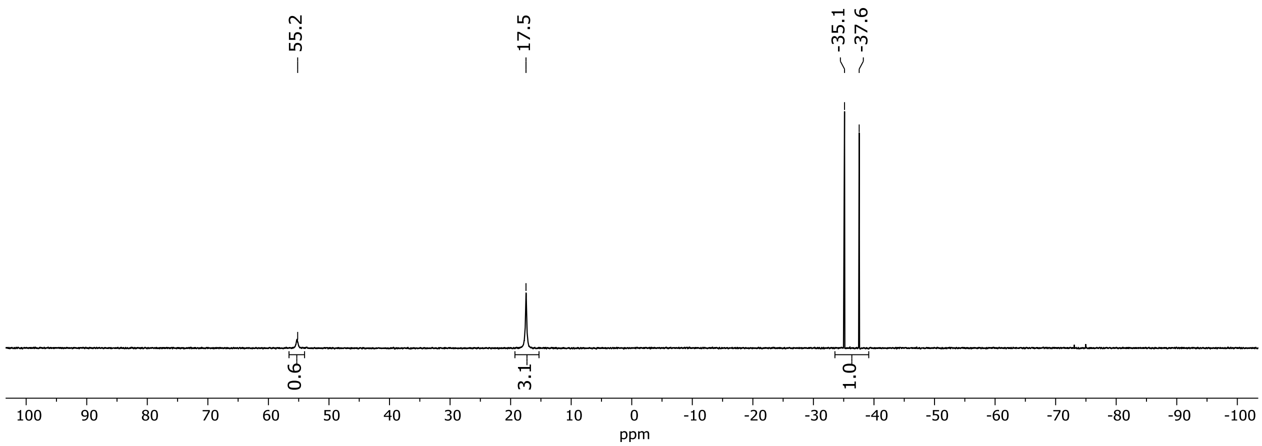

Figure S13: ^{19}F NMR spectrum (THF-d_8, 376 MHz, D_1 = 20 sec, aq. time = 0.55 sec., zg) of **4**.

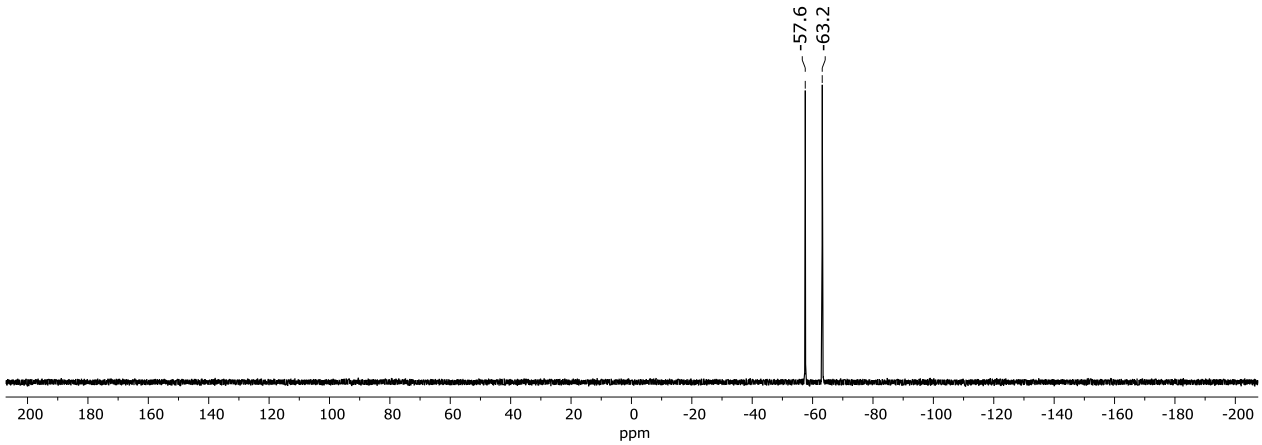

Figure S14: ^{31}P NMR spectrum (THF-d_8, 162 MHz) of **4**.

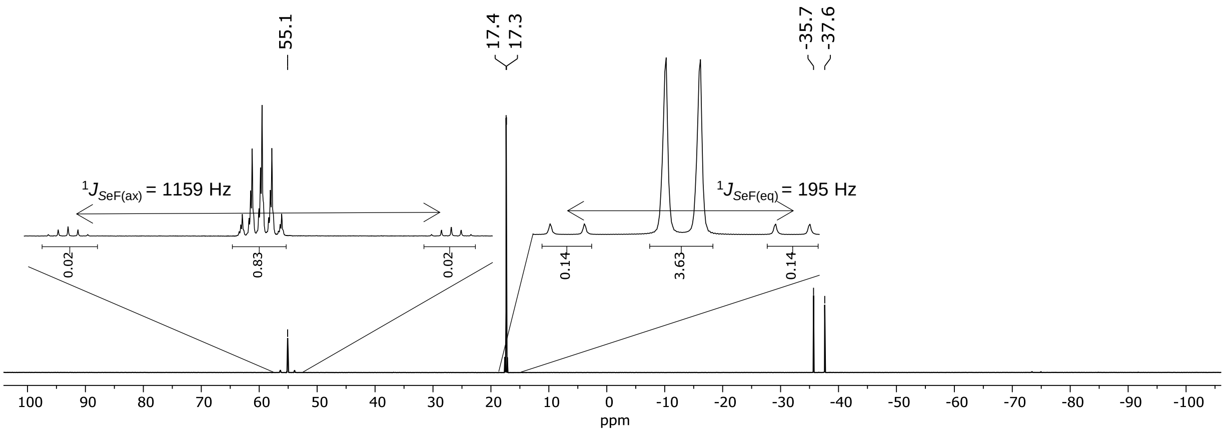

Figure S15: ^{19}F NMR spectrum (THF-d_8, 471 MHz, D_1 = 40 sec, aq. time = 0.44 sec., zg) of the reaction mixture of the reaction of **2** with elemental selenium after heating 72 hours at 120 °C.

1.4 Preparation of 5

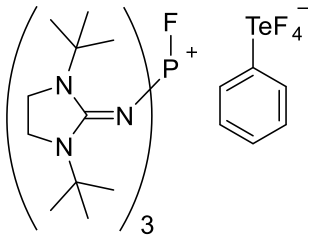

The SF_5^- salt **2** (100 mg, 0.131 mmol, 1.0 eq.) was added to a solution of Ph_2Te_2 (27 mg, 0.065 mmol, 0.5 eq.) in THF (5 mL). The mixture was stirred in a sealed glass vessel for 14 hours at 70 °C. The reaction mixture was then cooled to ambient temperature and the volume was reduced to 2 mL. The product was obtained as colorless crystals by slow diffusion of diethyl ether into the reaction mixture. The crystalline material was separated by filtration, washed with diethyl ether, and dried *in vacuo*.

Note: The reaction can also be performed at ambient temperature. Full conversion of **2** (0.065 mmol) was then observed after 3 days.

Yield: 92% (110 mg, 0.120 mmol).

^{1}H NMR (500 MHz, 300 K, MeCN-d_3): δ = 8.04–7.96 (m, 2 H, *o*-H_{TeF4Ph}), 7.44–7.38 (m, 2 H, *m*-H_{TeF4Ph}),
7.36–7.31 (m, 1 H, *p*-H_{TeF4Ph}), 3.35 (s, 12 H, CH_2), 1.37 (s, 54 H, CH_3).

^{13}C{^{1}H} NMR (126 MHz, 300 K, MeCN-d_3): δ = 163.2 (*i*-C_{TeF4Ph}), 152.68 (dd, $^2J_{CP}$ = 33.6, $^3J_{CF}$ = 2.5, *C*=N), 131.4 (*o*-C_{TeF4Ph}),129.8 (*p*-C_{TeF4Ph}), 128.4 (*m*-C_{TeF4Ph}), 56.2 (*C*($CH_3)_3$), 43.3 (*C*H_2), 28.9 (C(*C*$H_3)_3$).

^{19}F NMR (471 MHz, 300 K, MeCN-d_3): δ = –37.4 (d, $^1J_{PF}$ = 911 Hz, R_3P*F*), -64.6 (s, Te*F*$_4$Ph).

^{31}P NMR (162 MHz, 300 K, MeCN-d_3): δ = –59.7 (d, $^1J_{PF}$ = 911 Hz).

^{125}Te{^{1}H} NMR (126 MHz, 300 K, MeCN-d_3): δ = 1349.3 (p, $^1J_{TeF} \approx$ 472 Hz).

HRMS (ESI, positive): m/z calc. for $[C_{33}H_{67}N_9PF]^+$ 638.51683, found: 638.52040.

HRMS (ESI, negative): m/z calc. for $[C_6H_5F_4Te]^-$ 282.93952, found: 282.93908.

Elemental analysis: calculated (%) for $C_{39}H_{71}F_5N_9PTe$: C 50.94, H 7.78, N 13.71, found C 51.00, H 7.21, N 13.79.

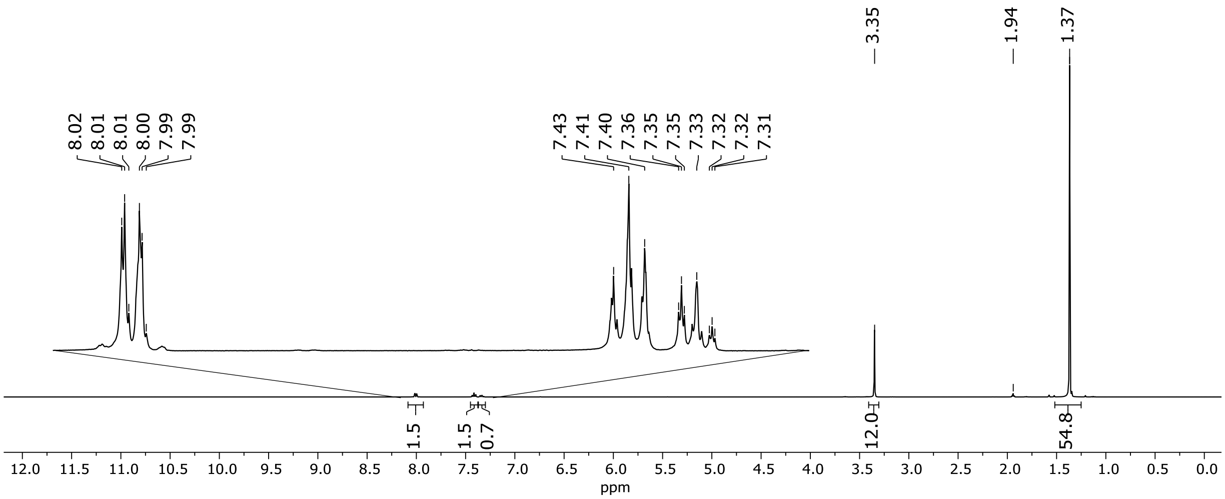

Figure S16: ^{1}H NMR spectrum (MeCN-d_3, 500 MHz) of **5**.

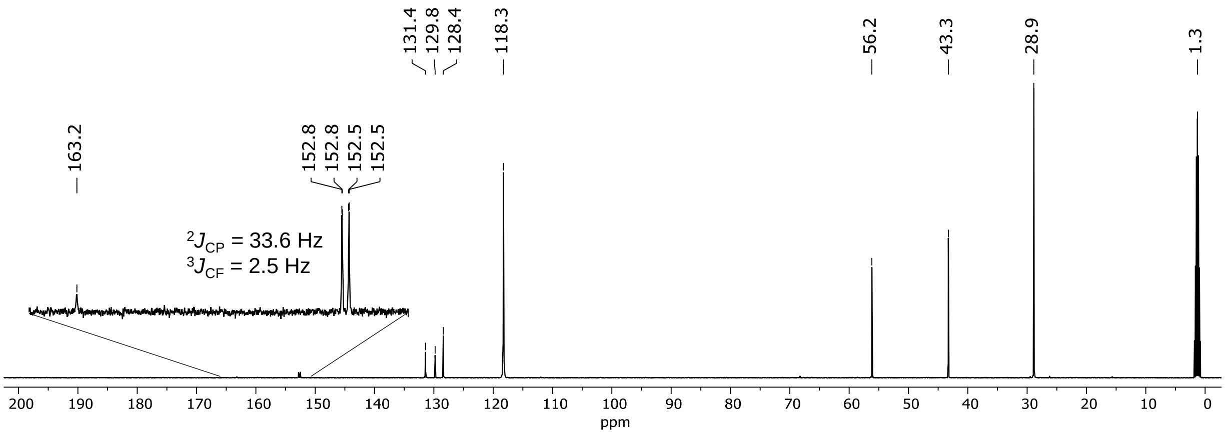

Figure S17: ^{13}C{^{1}H} NMR spectrum (MeCN-d_3, 126 MHz) of **5**.

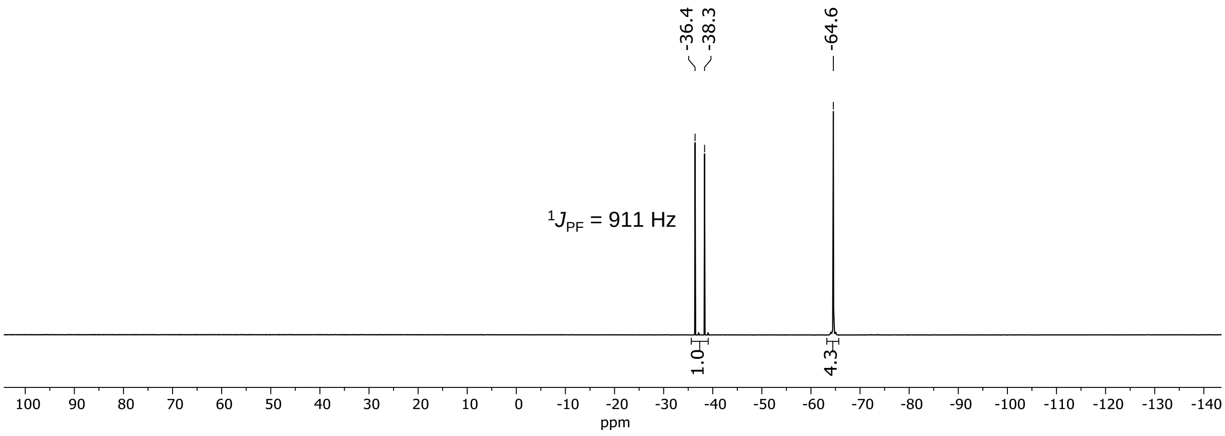

Figure S18: ^{19}F NMR spectrum (MeCN-d_3, 471 MHz, D_1 = 30 sec, aq. time = 1.10 sec., zg) of **5**.

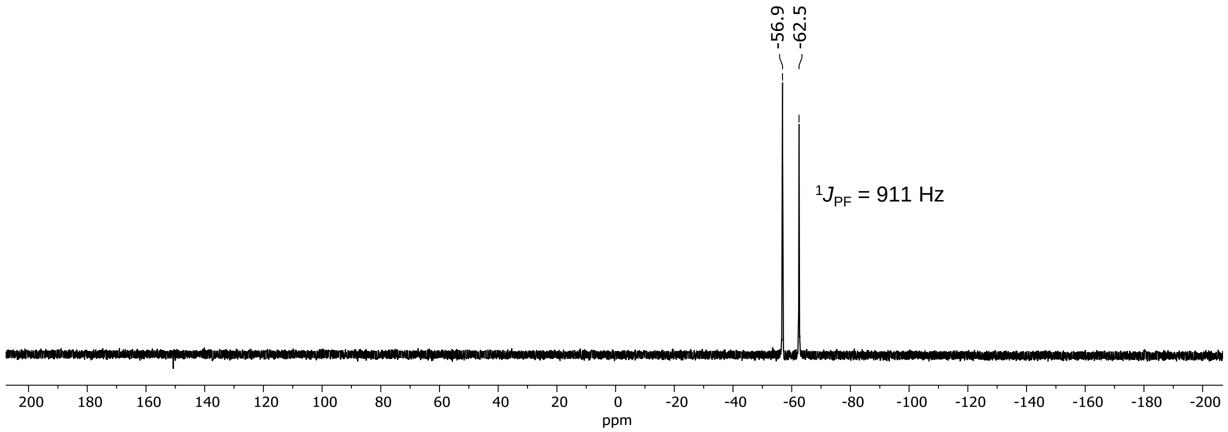

Figure S19: ^{31}P NMR spectrum (MeCN-d_3, 162 MHz) of **5**.

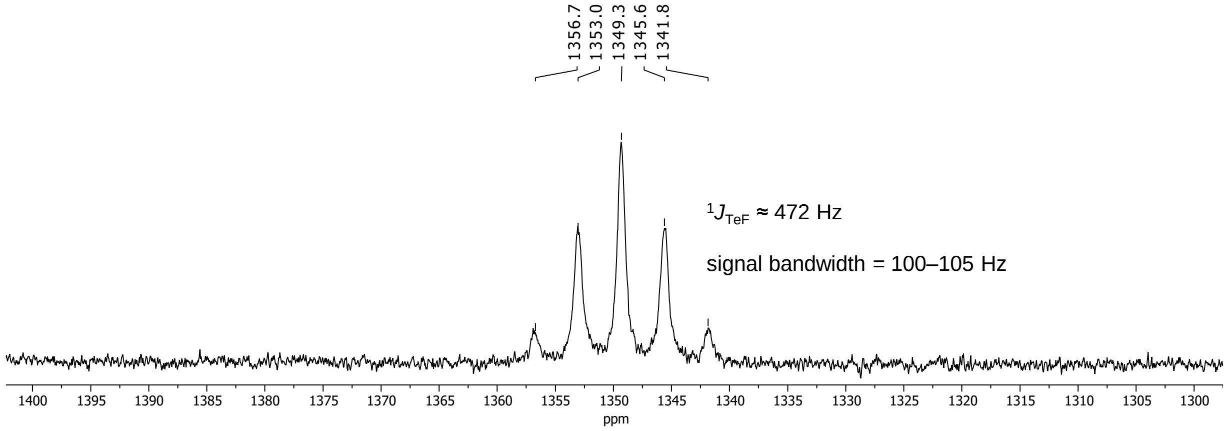

Figure S20: ^{125}Te{^{1}H} NMR spectrum (MeCN-d_3, 126 MHz) of **5**.

1.5 Preparation of 6

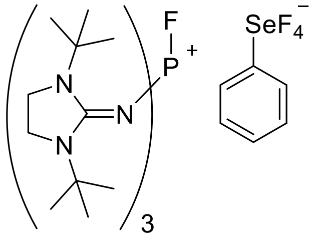

An NMR tube equipped with an PTFE inlay was charged with a suspension of Se_2Ph_2 (10 mg, 0.033 mmol, 0.5 eq.) and **2** (51 mg, 0.066 mmol, 1.0 eq) in THF (0.3 mL). The NMR tube was sealed with a PTFE valve and it was heated at 90 °C for 48 hours. After cooling the reaction mixture to room temperature, the THF solution was carefully layered with diethyl ether (1 mL). After two days the formed crystals were isolated and washed with diethyl ether (2 × 1 ml) using a syringe to remove the solvent. After drying *in vacuo*, compounds **6** was obtained as pale yellow, crystalline solid.

Note: The compound decomposes slowly in the presence of glass, as indicated by an increasing intensity of the $[FHF]^-$ resonance in the ^{1}H NMR spectrum. The NMR measurements were therefore carried out using NMR tubes with a PTFE inlay.

Yield: 60% (34 mg, 0.039 mmol).

^{1}H NMR (400 MHz, 300 K, DCM-d_2): δ = 8.14–8.06 (m, 2 H, o-H_{SeF4Ph}), 7.39–7.26 (m, 3 H, m-H_{SeF4Ph}, p-H_{SeF4Ph}), 3.34 (s, 12 H, CH_2), 1.36 (s, 54 H, CH_3).
^{13}C{^{1}H} NMR (101 MHz, 300 K, DCM-d_2): δ = 164.2 (i-C_{SeF4Ph}), 151.2 (d, $^{2}J_{CP}$ = 35.9 Hz, C=N), 129 (o-C_{SeF4Ph}), 127.9 (p-C_{SeF4Ph}), 127.4 (m-C_{SeF4Ph}), 55.6 ($C(CH_3)_3$), 42.7 (CH_2), 28.7 ($C(CH_3)_3$).
^{19}F NMR (376 MHz, 300 K, DCM-d_2): δ = –25.0 (br, SeF_4Ph), –35.3 (d, $^{1}J_{PF}$ = 907.9 Hz, R_3PF).
^{31}P NMR (162 MHz, 300 K, DCM-d_2): δ = –62.2 (d, $^{1}J_{PF}$ = 907.9 Hz).
^{77}Se NMR (76 MHz, 300 K, DCM-d_2): δ = 1022.7.
HRMS (ESI, positive): m/z calc. for $[C_{33}H_{67}N_9PF]^+$ 638.51683, found: 638.51918.
Note: The $[PhSeF_4]^-$ anion was not detected in the negative HRMS-ESI Mode.

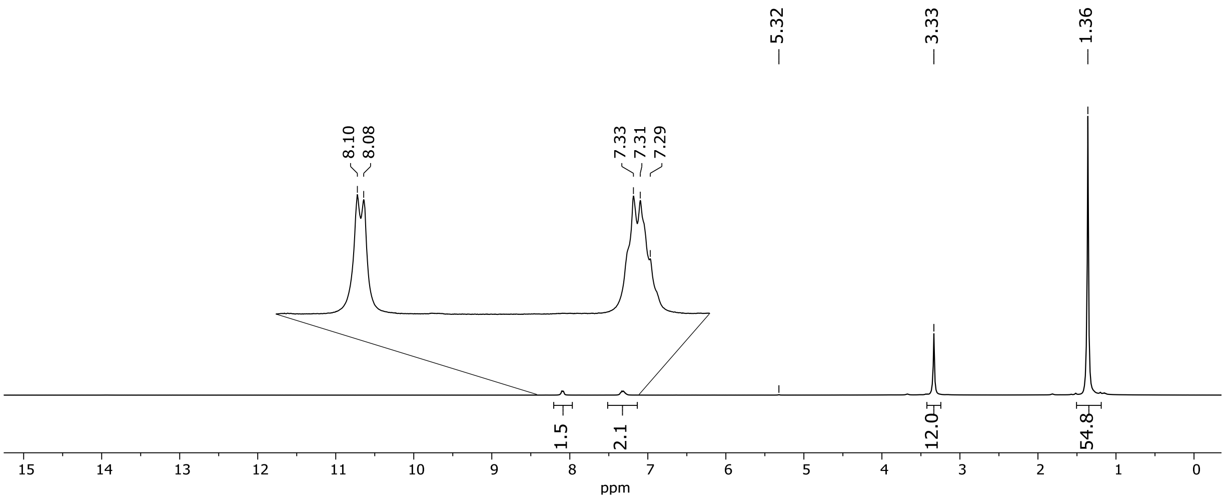

Figure S21: ^{1}H NMR spectrum (DCM-d_2, 400 MHz) of **6**.

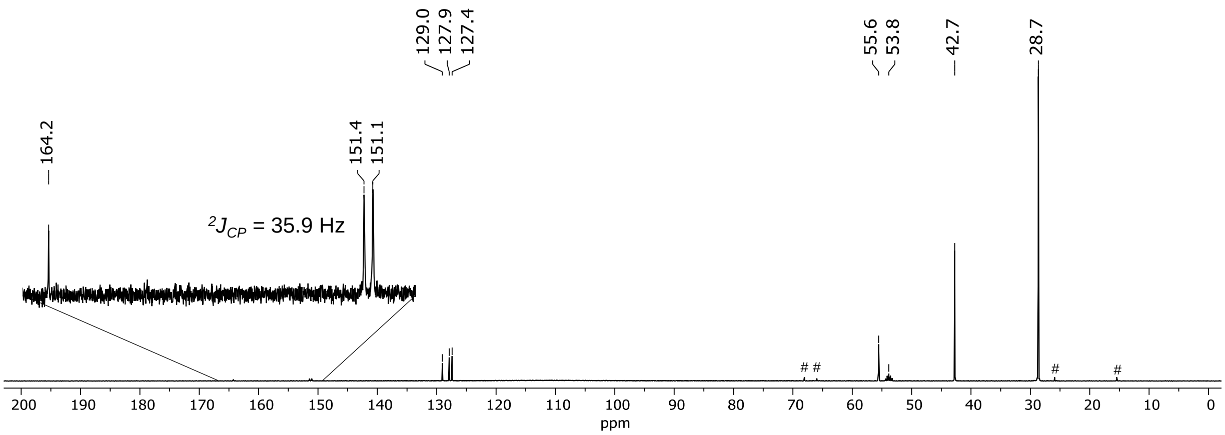

Figure S22: ^{13}C{^{1}H} NMR spectrum (DCM-d_2, 101 MHz) of **6**. # Signals of the solvent Et_2O and THF.

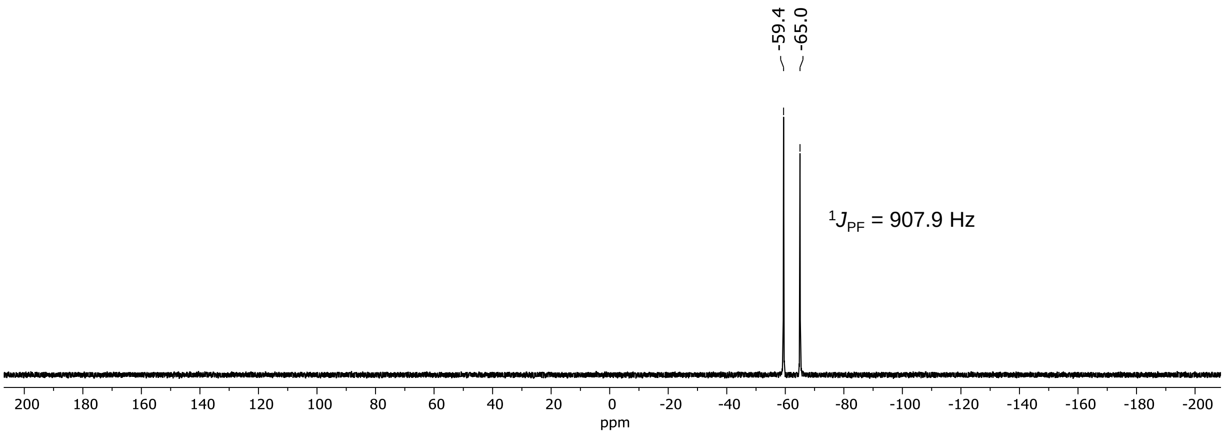

Figure S23: ^{31}P NMR spectrum (DCM-d_2, 162 MHz) of **6**.

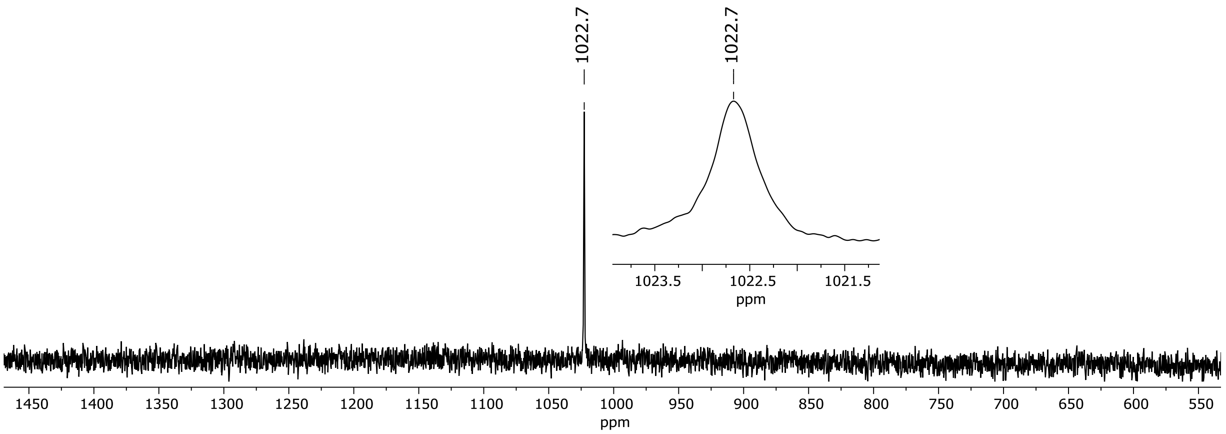

Figure S24: ^{77}Se NMR spectrum (DCM-d_2, 76 MHz) of **6**.

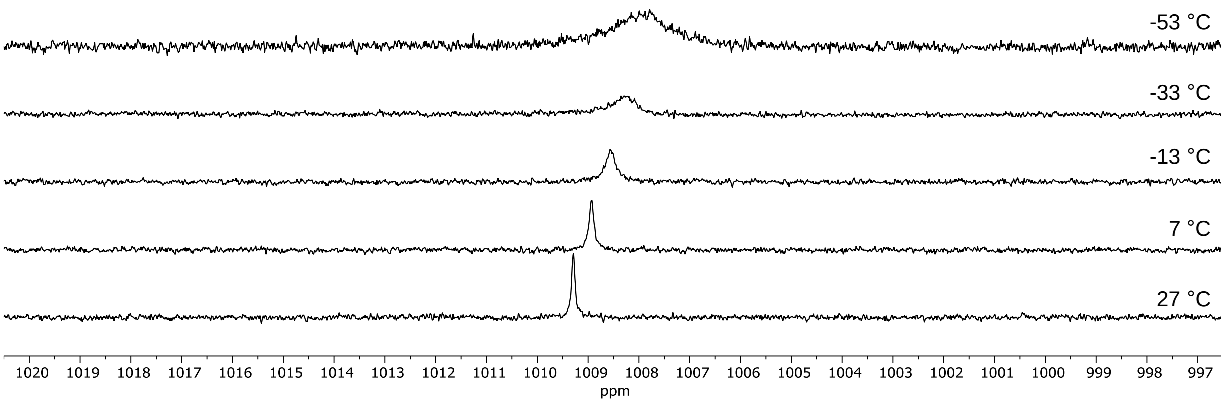

Figure S25: Variable temperature ^{77}Se NMR spectra (THF-d_8, 96 MHz) of **6** starting from 27 °C (bottom) to -53 °C (top).

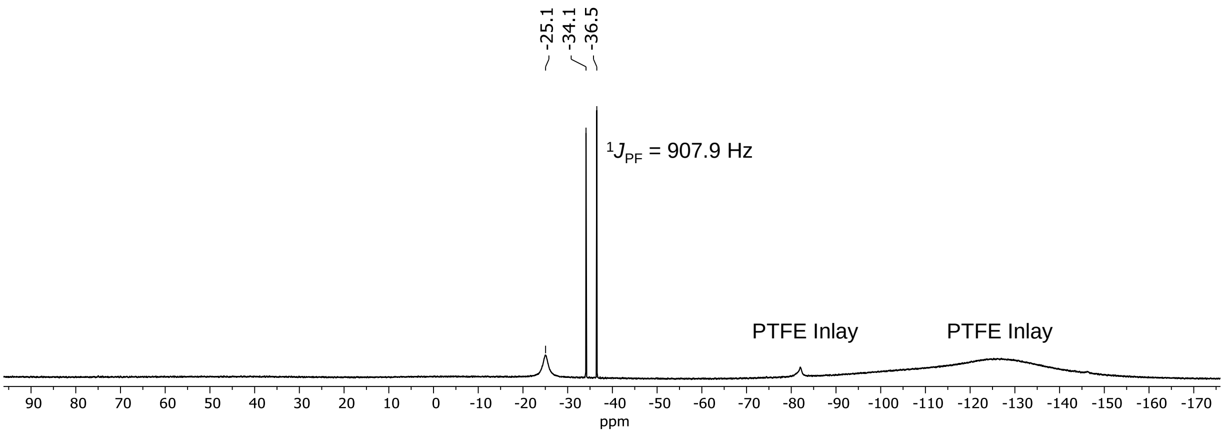

Figure S26: ^{19}F NMR spectrum (DCM-d_2, 376 MHz) of **6**.

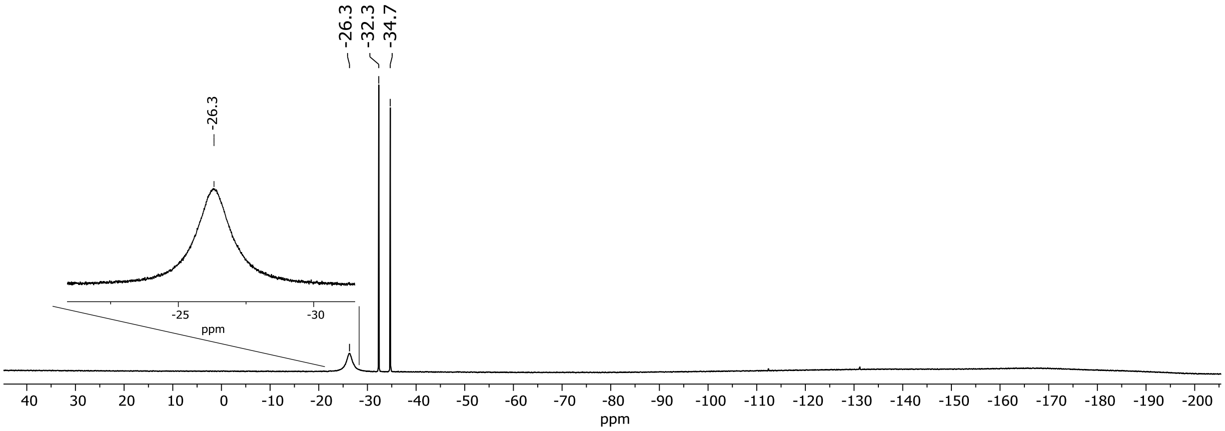

Figure S27: ^{19}F NMR spectrum (DCM-d_2, 376 MHz) of **6** at -45 °C.

1.6 Trapping experiments to identify the sulfur species formed in the synthesis of 3 and 5 using triphenylphosphine

The trapping experiments were performed for the tellurium compounds **3** and **5** since the selenium compounds **4** and **6** react with triphenylphosphine.

After heating THF solutions of either **2** and elemental tellurium (reaction **A**) or of **2** and diphenyl ditelluride (reaction **B**) for 48 hours at 70 °C, triphenylphosphine (approx. 4 eq.) was added to the resulting reaction mixtures, respectively (Scheme S1). Quantitative ^{19}F and ^{31}P NMR measurements were performed to determine the relative amounts of difluorotriphenylphosphorane and triphenylphosphine sulfide compared to the fluorophosphonium cation of **3** and **5**, respectively (Table S2). For reaction **A**, $SPPh_3$ is formed stoichiometrically along with small amounts of F_2PPh_3, indicating that elemental sulfur is formed during the reaction and that some SF species was still present in the reaction mixture (presumably residual SF_5^- salt). For reaction **B**, $SPPh_3$ and F_2PPh_3 are formed in a 2:1 ratio, consistent with the formation of SSF_2 during the reaction. The resonance of thiothionylfluoride[4] was detected in the ^{19}F NMR spectrum of the reaction mixture of **2** with diphenyl ditelluride (Figure S32).

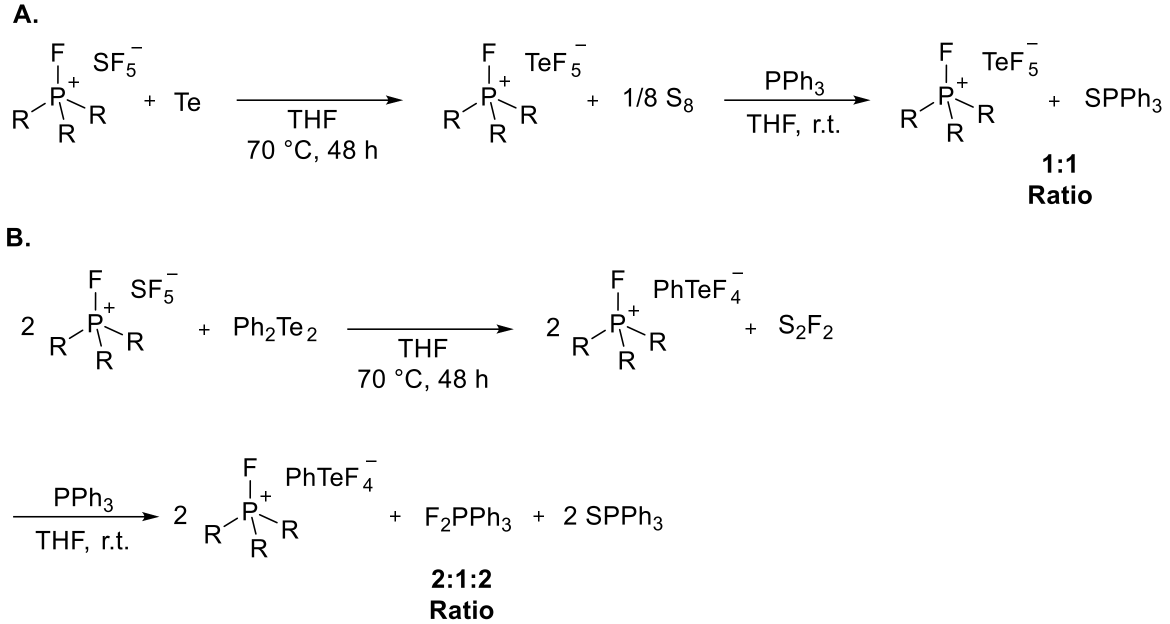

Scheme S1: Reaction of **2** with elemental tellurium (top, **A**) and of **2** with diphenyl ditelluride (bottom, **B**) and the subsequent addition of an excess of PPh_3 to identify the "SF" species formed in the reaction.

Table S2: Relative amounts of F_2PPh_3 and $SPPh_3$ compared to $(FPR_3)^+$ according to ^{19}F NMR analysis of the reaction solutions.

Addition of PPh_3 to the reaction mixture of the preparation of	Yield of F_2PPh_3 (%) with respect to **2**	Yield of $SPPh_3$ (%) with respect to **2**
$[FP(NsI\textit{t}Bu)_3][TeF_5]$ (**3**)	16	93
$[FP(NsI\textit{t}Bu)_3][PhTeF_4]$ (**5**)	50	99

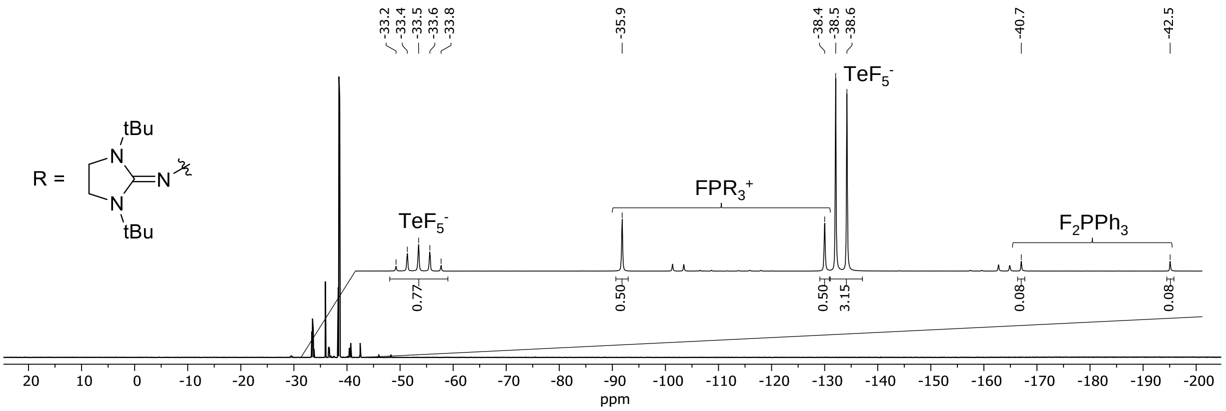

Figure S28: ^{19}F NMR spectrum (THF-d_8, 376 MHz, D_1 = 20 sec, aq. time = 0.55 sec., zg) of the reaction mixture of **2** and elemental tellurium after 48 h at 70 °C and subsequent addition of triphenylphosphine. Small, but non-stoichiometric amounts of F_2PPh_3 were detected.

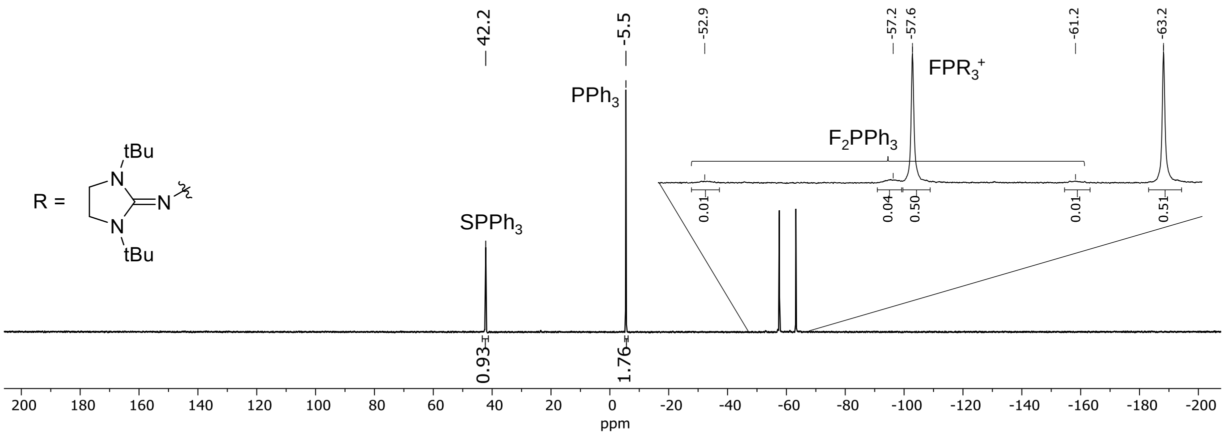

Figure S29: ^{31}P NMR spectrum (THF-d_8, 162 MHz, D_1 = 20 sec, aq. time = 1.12 sec., zg) of the reaction mixture of **2** and elemental tellurium after 48 h at 70 °C and subsequent addition of triphenylphosphine. Small, but non-stoichiometric amounts of F_2PPh_3 were detected.

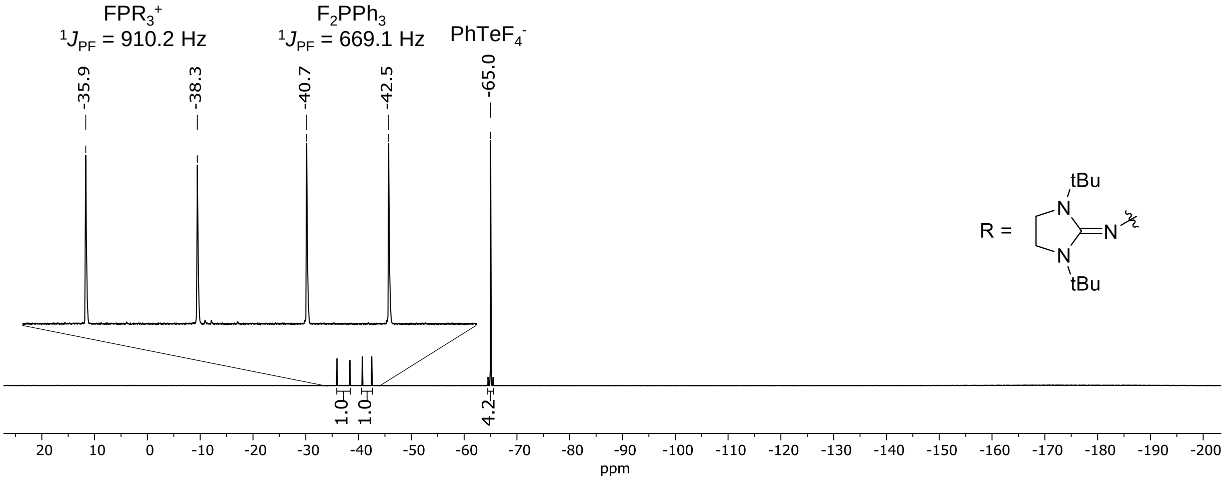

Figure S30: ^{19}F NMR spectrum (THF-d_8, 471 MHz) of the reaction mixture of **2** and Ph_2Te_2 after 48 h at 70 °C and subsequent addition of triphenylphosphine.

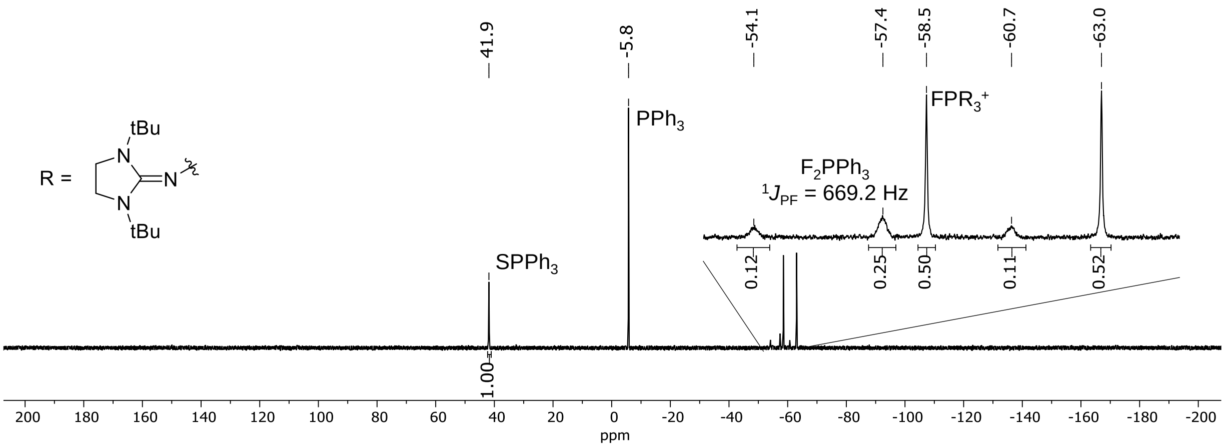

Figure S31: ^{31}P NMR spectrum (THF-d_8, 202 MHz, D_1 = 60 sec, aq. time = 0.81 sec., zg) of the reaction mixture of **2** and Ph_2Te_2 after 48 h at 70 °C and subsequent addition of triphenylphosphine.

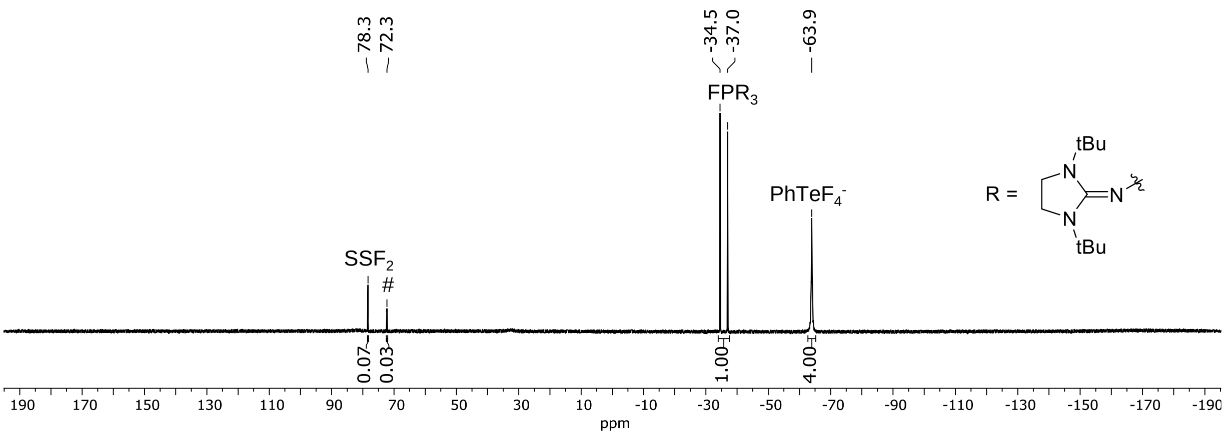

Figure S32: ^{19}F NMR spectrum (THF, 376 MHz, D_1 = 30 sec, aq. time = 1.10 sec., zg) of the reaction mixture of **2** and Ph_2Te_2 after 72 hours. #: unidentified signal.

1.7 Synthesis of 7 by oxidation of 5 ($PhTeF_4$) with *m*-CPBA

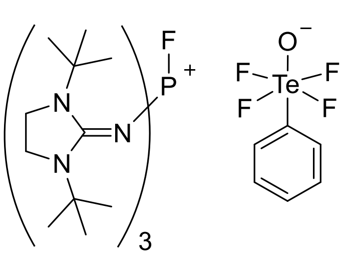

5 (184 mg, 0.20 mmol, 1.0 eq.) was dissolved in DCM (3 mL). Anhydrous *m*-CPBA dissolved in DCM (0.086 M, 2.56 mL, 1.1 eq.) was added and the reaction mixture was stirred for 30 minutes at ambient temperature. Quantitative oxidation of the anion was indicated by NMR spectroscopy. To remove the remaining *m*-CPBA and the *meta*-chlorobenzoic acid formed in the reaction, the following workup was carried out: The volume of the DCM solution was reduced to approximately 0.5 mL, diethyl ether (7 mL) was added, and the mixture was vigorously shaken for 60 minutes. The white precipitate was then isolated and washed with diethyl ether (8 × 3 mL). The precipitate was again dissolved in DCM and the whole procedure was repeated once more. Crystals of **7** were obtained by slow diffusion of diethyl ether

into a saturated DCM solution of **7** at -40 °C. After drying the precipitate *in vacuo*, **7** was obtained as a white, crystalline solid.

Yield: 65% (121 mg, 0.13 mmol).

^{1}H NMR (400 MHz, 300 K, MeCN-d_3): δ = 7.89–7.83 (m, 2 H, *m*-$H_{OTeF4Ph}$), 7.50–7.44 (m, 3 H, *o*-$H_{OTeF4Ph}$, *p*-$H_{OTeF4Ph}$), 3.34 (s, 12 H, CH_2), 1.36 (s, 54 H, CH_3).

^{13}C{^{1}H} NMR (75 MHz, 300 K, MeCN-d_3): δ = 158.6 (p, $^{1}J_{CF}$ = 28.5 Hz, *i*-$C_{OTeF4Ph}$), 152.6 (dd, $^{2}J_{CP}$ = 33.5, $^{3}J_{CF}$ = 2.5, *C*=N), 131.5–131.3 (m, *p*-$C_{OTeF4Ph}$, *m*-$C_{OTeF4Ph}$), 129.8 (p, $^{1}J_{CF}$ = 1.8 Hz, *o*-C_{TeF4Ph}), 56.1 (*C*(CH_3)$_3$), 43.3 (*C*H_2), 28.8 (d, $^{5}J_{PC}$ = 1.5 Hz, C(*C*H_3)$_3$).

^{19}F NMR (376 MHz, 300 K, MeCN-d_3): δ = -35.2 (s, OTe*F*$_4$Ph, $^{1}J_{125TeF}$ = 3230.6 Hz), -37.4 (d, $^{1}J_{PF}$ = 910.7 Hz, R_3P*F*).

^{31}P NMR (162 MHz, 300 K, MeCN-d_3): δ = -59.9 (d, $^{1}J_{PF}$ = 910.7 Hz).

^{125}Te{^{1}H} NMR (126 MHz, 300 K, MeCN-d_3): δ = 697.0 (p, $^{1}J_{TeF}$ = 3230.6 Hz).

HRMS (ESI, positive): m/z calc. for $[C_{33}H_{67}N_9PF]^+$ 638.5168, found: 638.5139.

HRMS (ESI, negative): m/z calc. for $[C_6H_5F_4OTe]^-$ 298.9344, found: 298.9340.

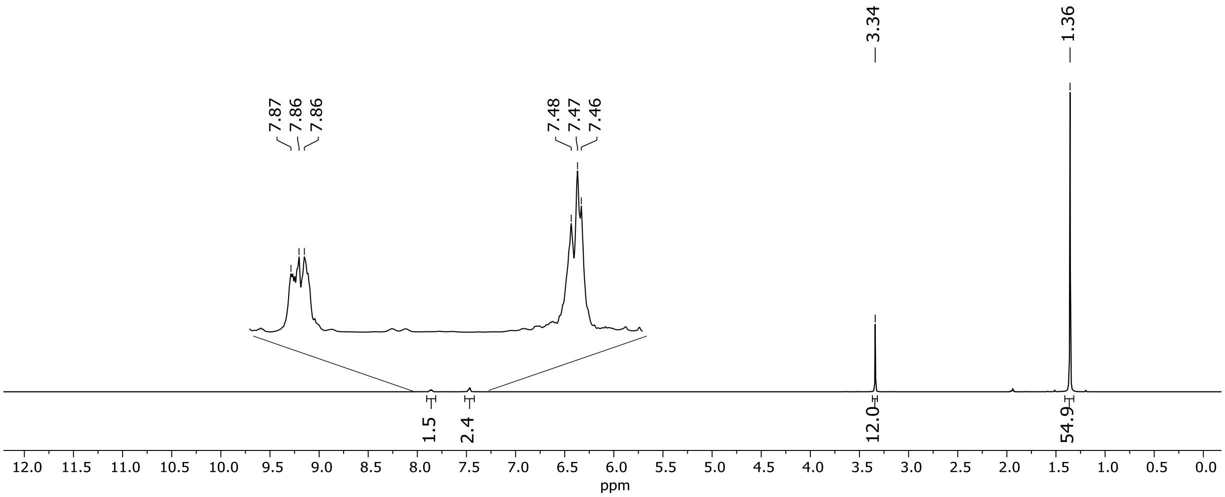

Figure S33: ^{1}H NMR spectrum (MeCN-d_3, 400 MHz) of **7**.

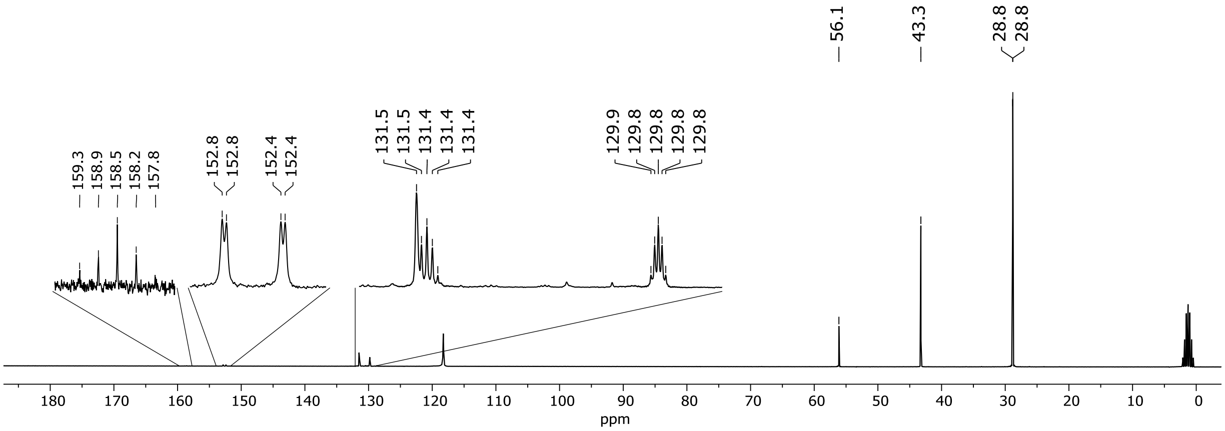

Figure S34: ^{13}C{^{1}H} NMR spectrum (MeCN-d_3, 75 MHz) of **7**.

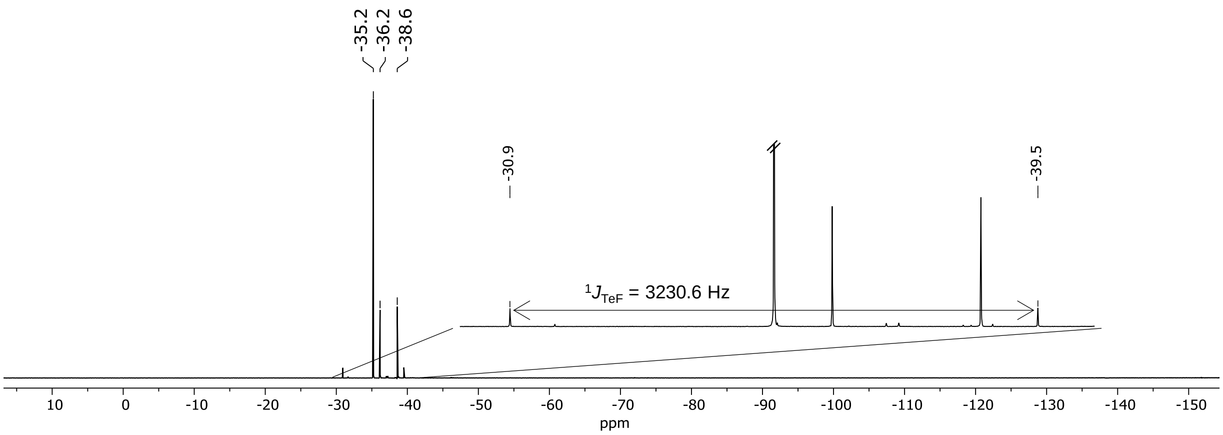

Figure S35: ^{19}F NMR spectrum (MeCN-d_3, 376 MHz) of **7**.

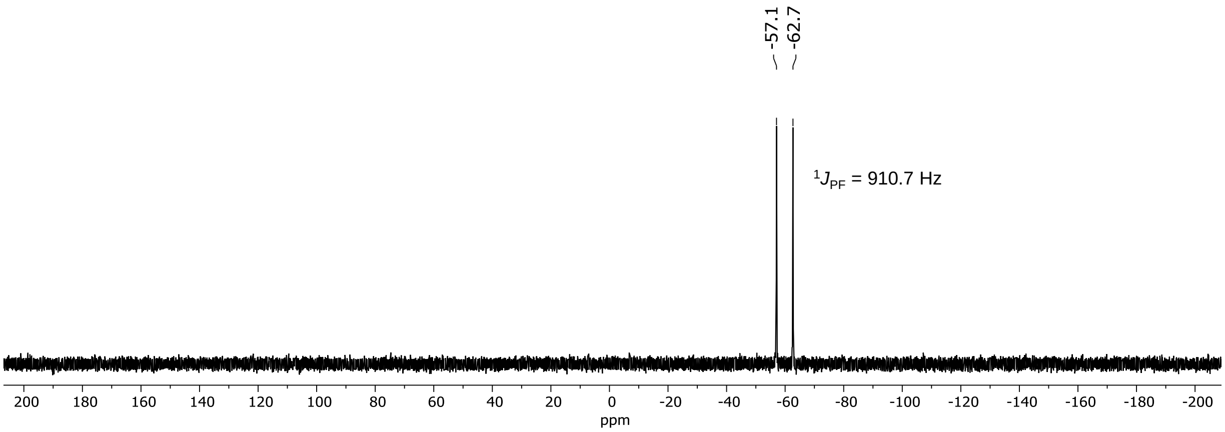

Figure S36: ^{31}P NMR spectrum (MeCN-d_3, 162 MHz) of **7**.

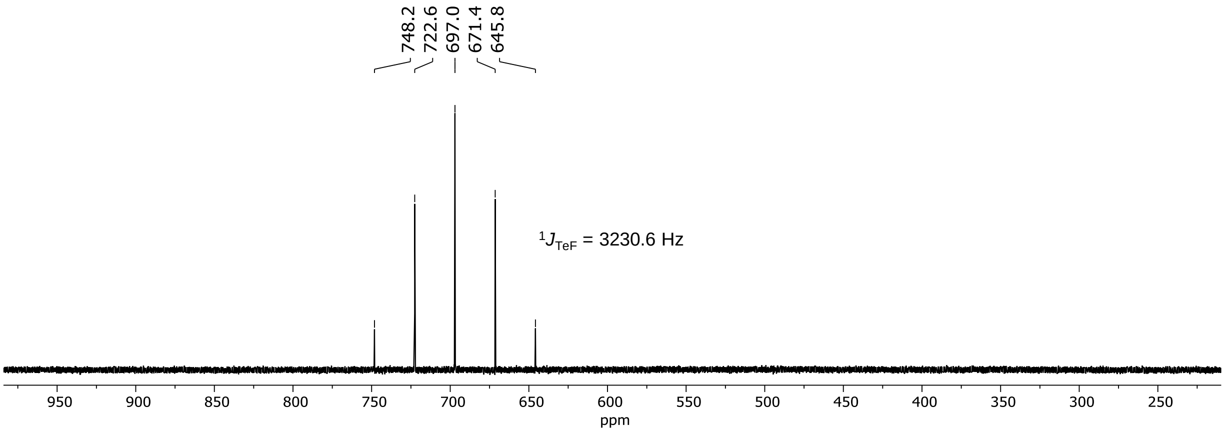

Figure S37: ^{125}Te{^{1}H} NMR spectrum (MeCN-d_3, 126 MHz) of **7**.

1.8 Attempts of oxidizing 6 with *m*-CPBA Synthesis

6 (55.7 mg, 0.064 mmol, 1.0 eq.) was dissolved in THF (0.3 mL) in a PTFE vessel and a solution of anhydrous *m*-CPBA in DCM (0.533 M, 0.13 ml, 1.1 eq) was added. The mixture immediately turned yellow and was stirred for 10 minutes at room temperature before being analyzed by NMR spectroscopy. The $^{19}F\{^{1}H\}$ NMR spectrum revealed the formation of 3-chlorobenzoyl fluoride as well as of fluorobenzene, indicating that the deoxyfluorination of 3-chlorperbenzoic acid took place with degradation of **6**. No resonances of a selenium species were found in the ^{19}F and ^{77}Se NMR spectra.

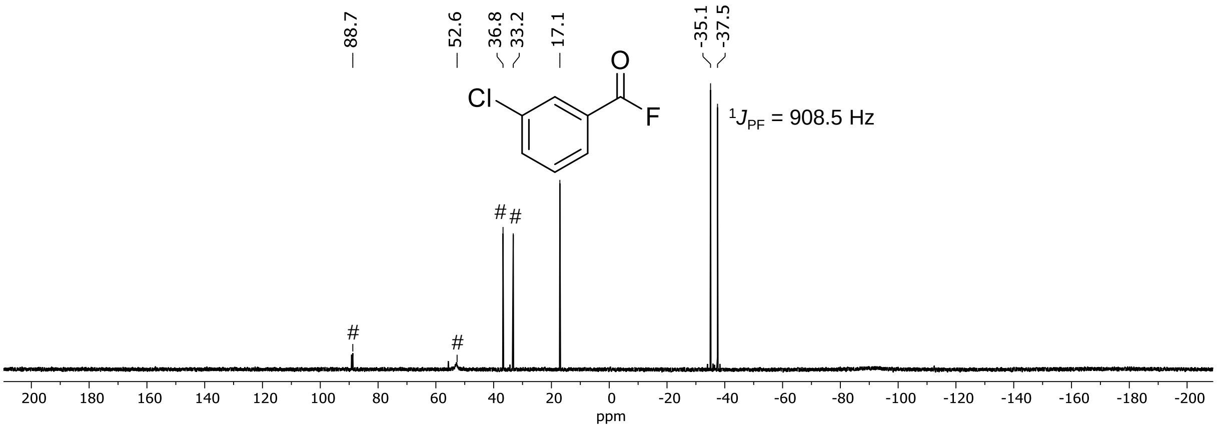

Figure S38: ^{19}F NMR spectrum (376 MHz, THF/DCM) of the reaction mixture of **6** and *m*-CPBA after 10 minutes at room temperature.

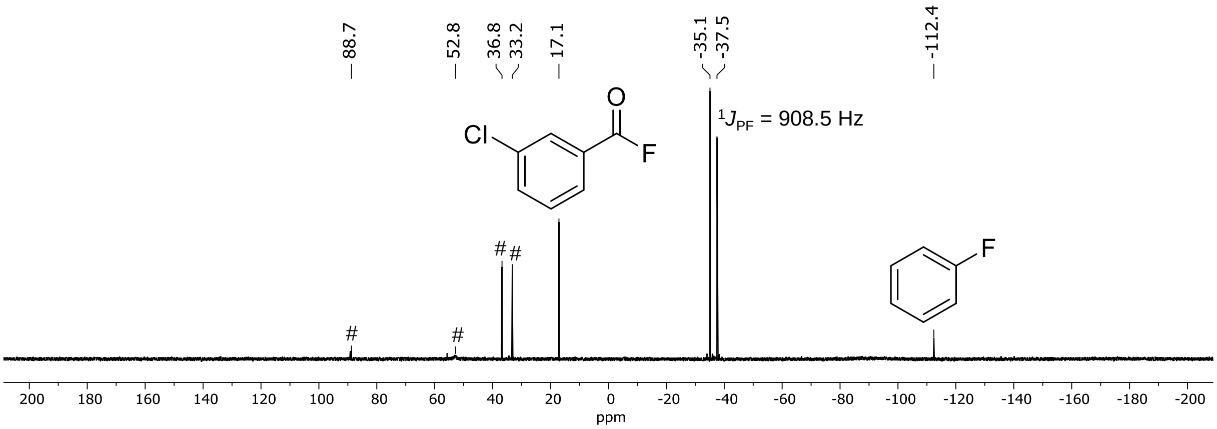

Figure S39: $^{19}F\{^{1}H\}$ NMR spectrum (376 MHz, THF/DCM) of the reaction mixture of **6** and *m*-CPBA after 10 minutes at room temperature.

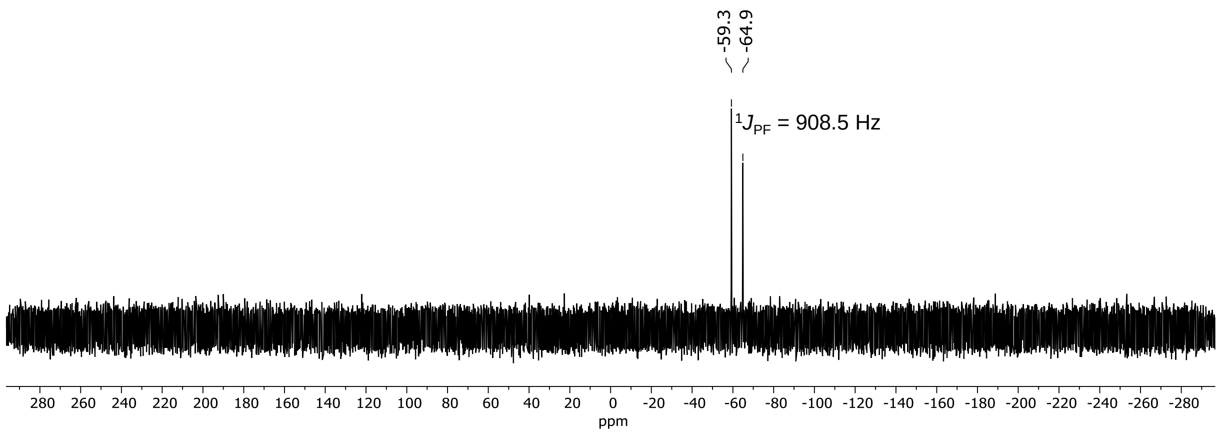

Figure S40: ^{31}P NMR spectrum (162 MHz, THF/DCM) of the reaction mixture of **6** and *m*-CPBA after 10 minutes at room temperature.

1.9 Synthesis of *trans*-alkoxytetrafluoro(phenyl)-λ^6-telluranes 8-10

General procedure: An excess of the respective alkylation reagent (0.45 mmol, 7.0 eq., dried over 3 Å molecular sieve) was added to a solution of **7** (60 mg, 0.064 mmol, 1.0 eq.) and MeCN (2 mL) in a PTFE tube. The tube was tightly sealed, and the mixture was stirred at 70 °C for 12 hours. The solution was then allowed to cool to room temperature and transferred into a Schlenk flask. An aliquot was taken to determine the conversion using quantitative ^{19}F NMR spectroscopy with *α,α,α*-trifluorotoluene as internal standard (Table S3). Information on the work up procedure for **8-10** are given in chapter 1.9.11.9.3.

Table S3: Alkylation of **7**. Yields were determined by quantitative ^{19}F NMR analysis using *α,α,α*-trifluorotoluene as internal standard.

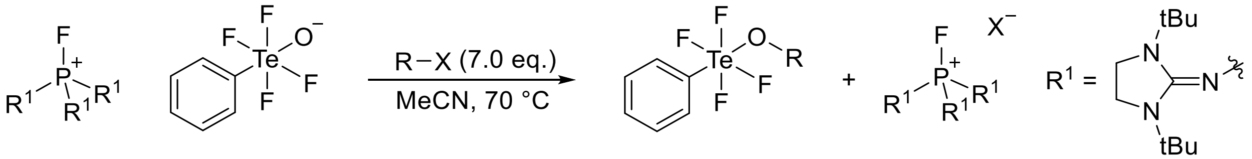

Reactant (R-X)	*Product*	*Yield (%)*
Methyl iodide	*trans*-PhTeF$_4$OMe (**8**)	75
Allyl iodide	*trans*-PhTeF$_4$OAllyl (**9**)	87
Benzyl bromide	*trans*-PhTeF$_4$OBenz (**10**)	70

1.9.1. Preparation of 10

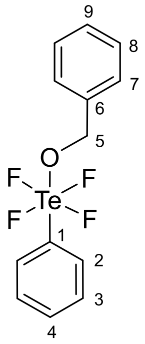

After the alkylation of **7** with benzyl bromide was carried out according to the general procedure, the volatiles were removed *in vacuo*. *n*-Hexane (10 mL) was added, and the suspension was vigorously stirred for 20 minutes. The suspension was filtered, and the solvent of the filtrate was removed *in vacuo*. The remaining oil was transferred onto a pad of silica and it was washed with *n*-hexane until the eluent contained no benzyl bromide. The product was then eluted using a mixture of DCM and *n*-hexane (1:3). All volatiles were then removed *in vacuo*. After recrystallization of **10** in a minimal amount of *n*-hexane (<1 mL) at -40 °C, it was obtained as an analytically pure white solid.

Isolated yield: 28% (7 mg, 0.018 mmol).

^{1}H NMR (400 MHz, 300 K, C_6D_6): δ = 7.67 (d, $^3J_{HH}$ = 8.1 Hz, 2H, H-2), 7.28–7.26 (m, 2H, H-7), 7.10–7.02 (m, 3H, H-8, H-9), 6.82–6.69 (m, 3H, H-3, H-4) 5.41 (s, 2H, H-5).

^{13}C{^{1}H} NMR (75 MHz, 300 K, C_6D_6): δ = 147.2–146.6 (m, C-1), 137.2 (C-6), 133.6 (C-3), 130.5–130.4 (m, C-2, C-4), 128.8, 128.8, 128.7 (C-7, C-8, C-9), 72.7 (p, $^3J_{CF}$ = 2.4 Hz, C-5).

^{19}F NMR (376 MHz, 300 K, C_6D_6): δ = -54.4 (s, $^1J_{125TeF}$ = 3548 Hz).

^{125}Te{^{1}H} NMR (126 MHz, 300 K, C_6D_6): δ = 726.1 (p, $^1J_{TeF}$ = 3548 Hz).

HRMS (ESI, negative): m/z calc. for $[C_{13}H_{12}F_3O_2Te]^-$ ([**10**-F+O]$^-$) 386.9857, found: 386.9858.

ATR-FTIR (neat): $\tilde{\nu}$ = 1443.5(w), 1018.0 (w), 995.7 (w), 955.3 ,760.3 (w), 738.9 (m), 705.7 (m), 678.1 (w), 629.4 (s), 587.6 (w), 485.3 (w), 460.9 (m) cm^{-1}.

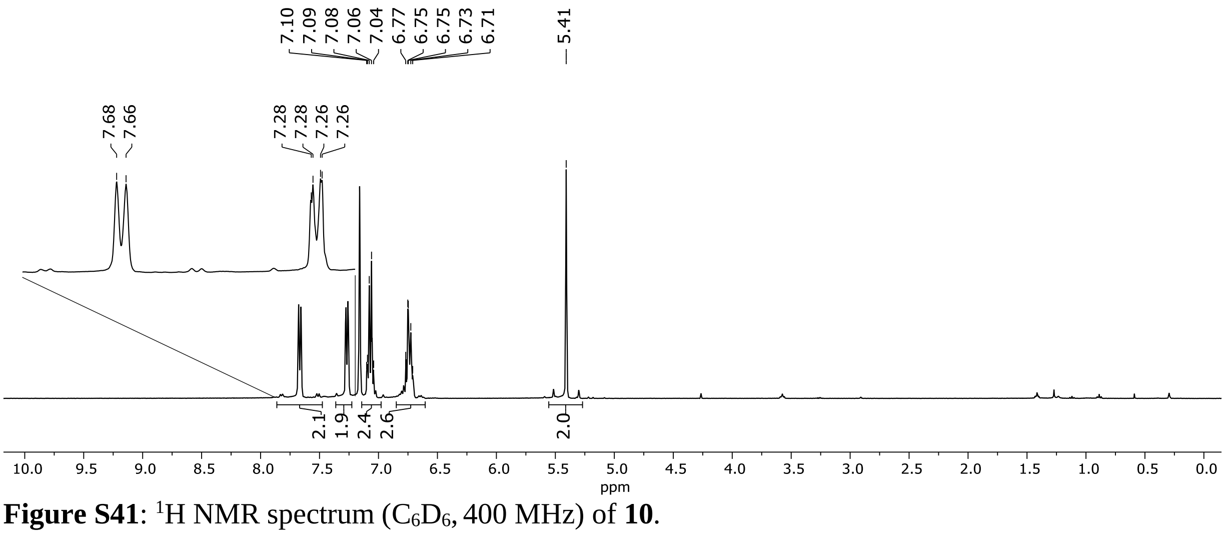

Figure S41: ^{1}H NMR spectrum (C_6D_6, 400 MHz) of **10**.

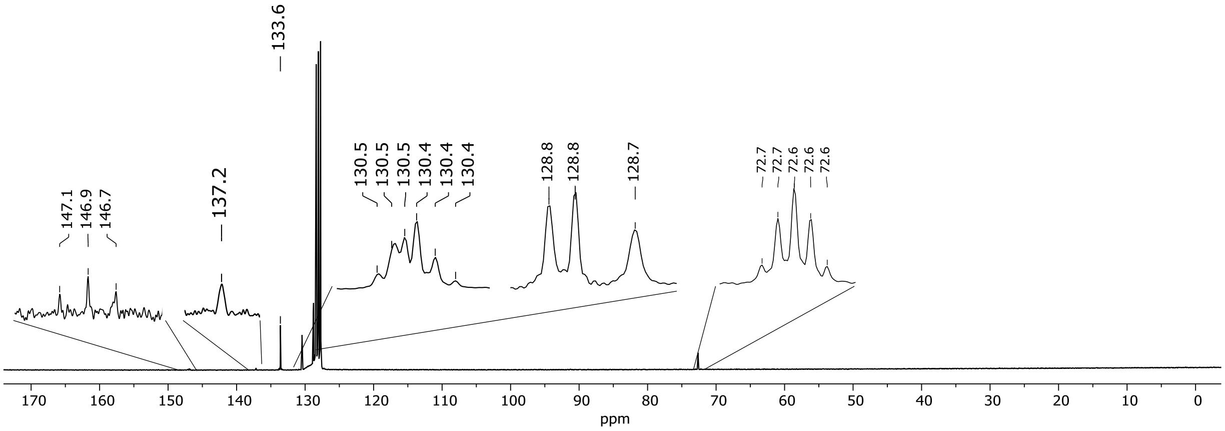

Figure S42: $^{13}C\{^{1}H\}$ NMR spectrum (C_6D_6, 75 MHz) of **10**.

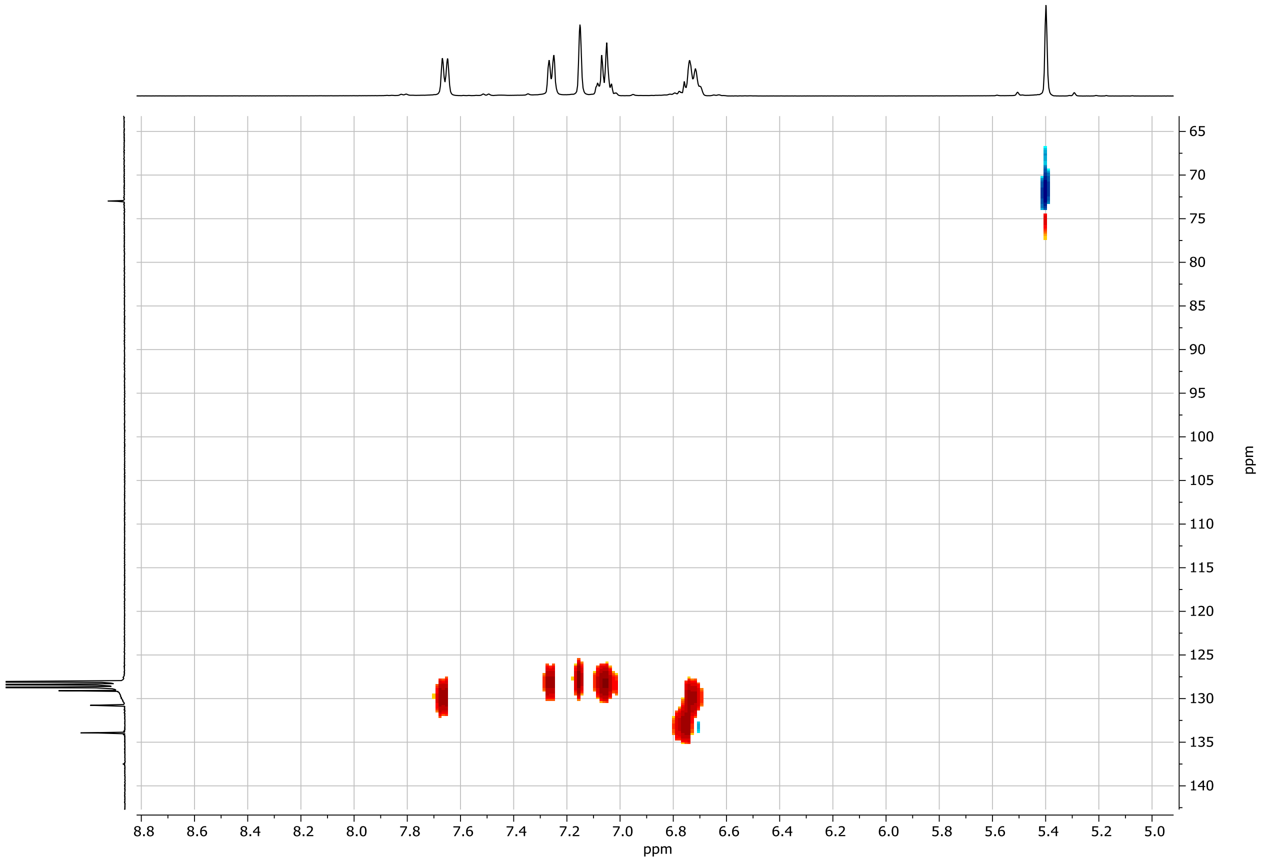

Figure S43: $^{1}H/^{13}C$ HSQC NMR spectrum of **10** in C_6D_6.

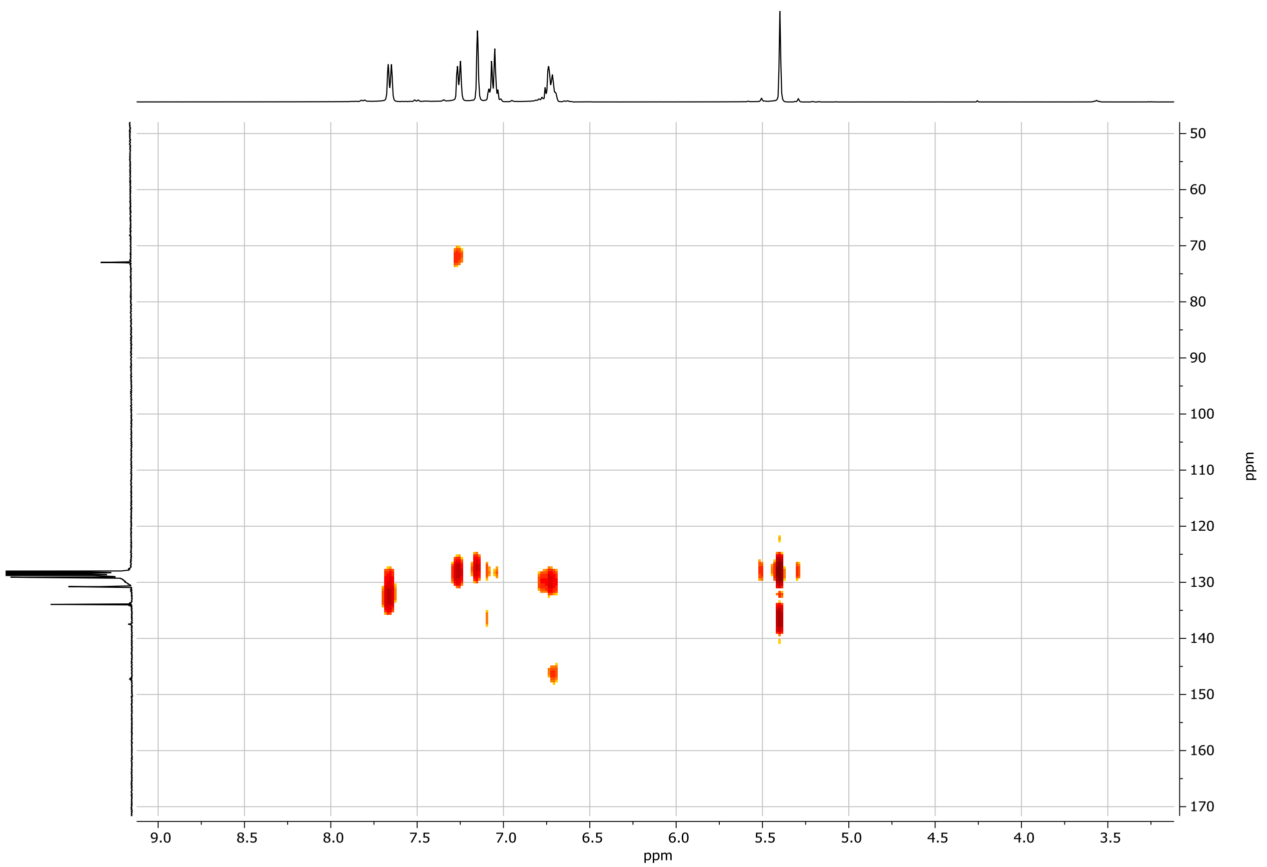

Figure S44: $^{1}H/^{13}C$ HMBC NMR spectrum of **10** in C_6D_6.

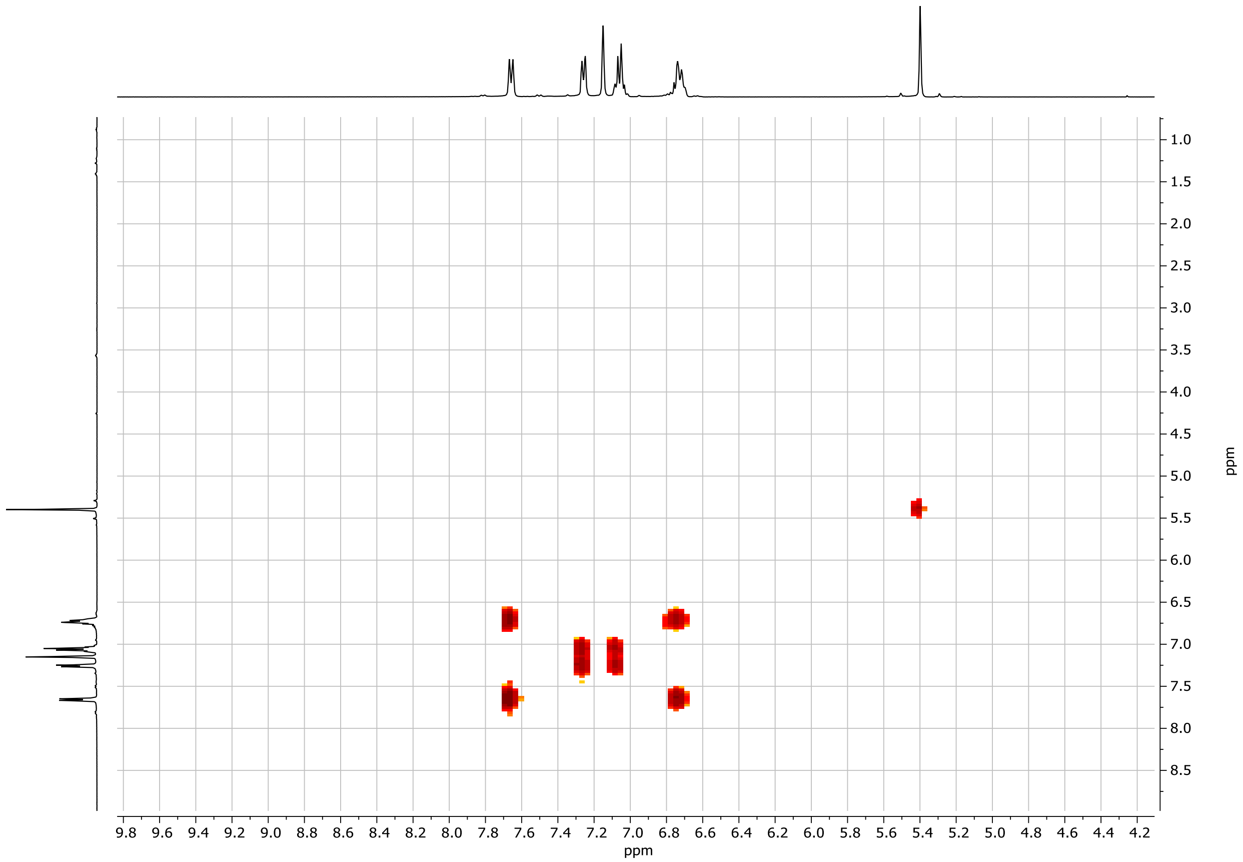

Figure S45: $^1H/^1H$ COSY NMR spectrum of **10** in C_6D_6.

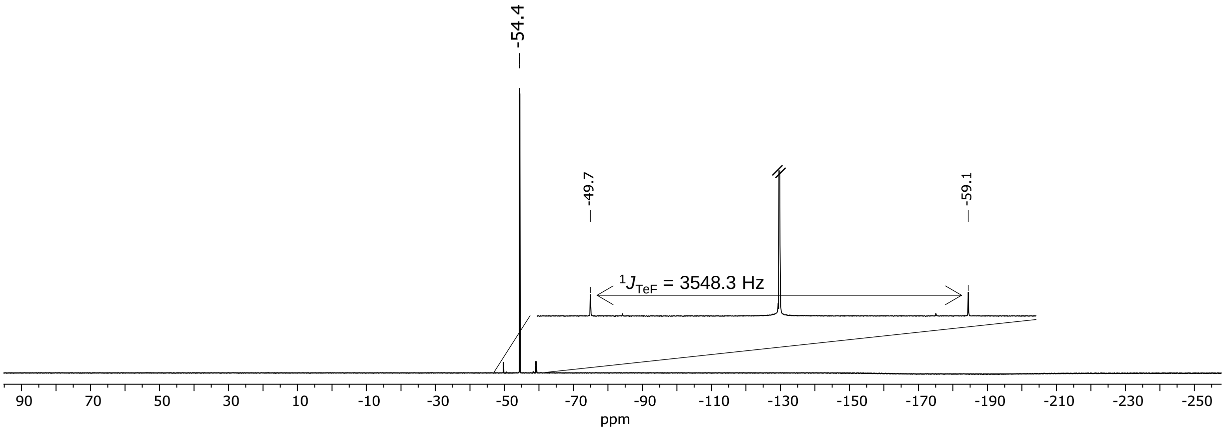

Figure S46: ^{19}F NMR spectrum (C_6D_6, 376 MHz) of **10**.

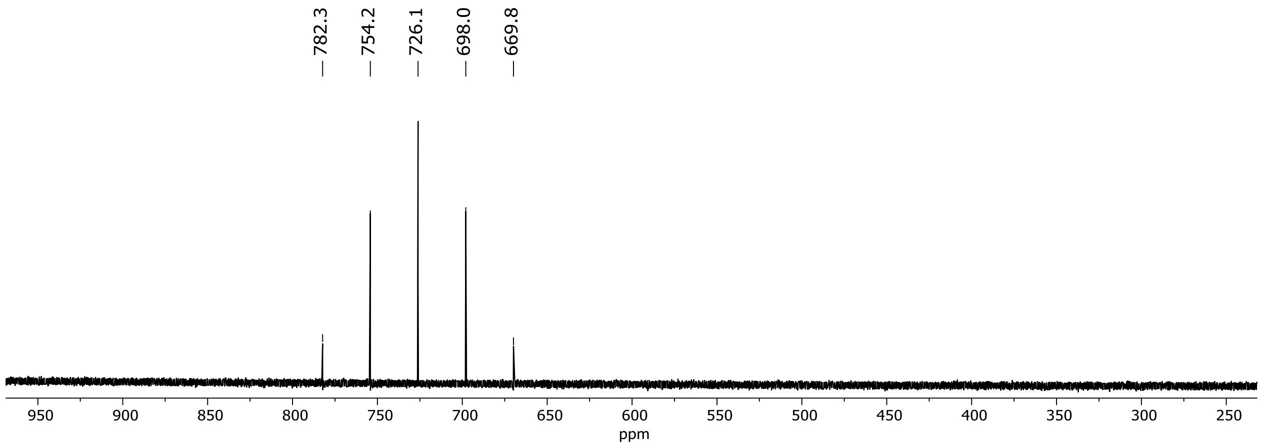

Figure S47: $^{125}Te\{^{1}H\}$ NMR spectrum (C_6D_6, 126 MHz) of **10**.

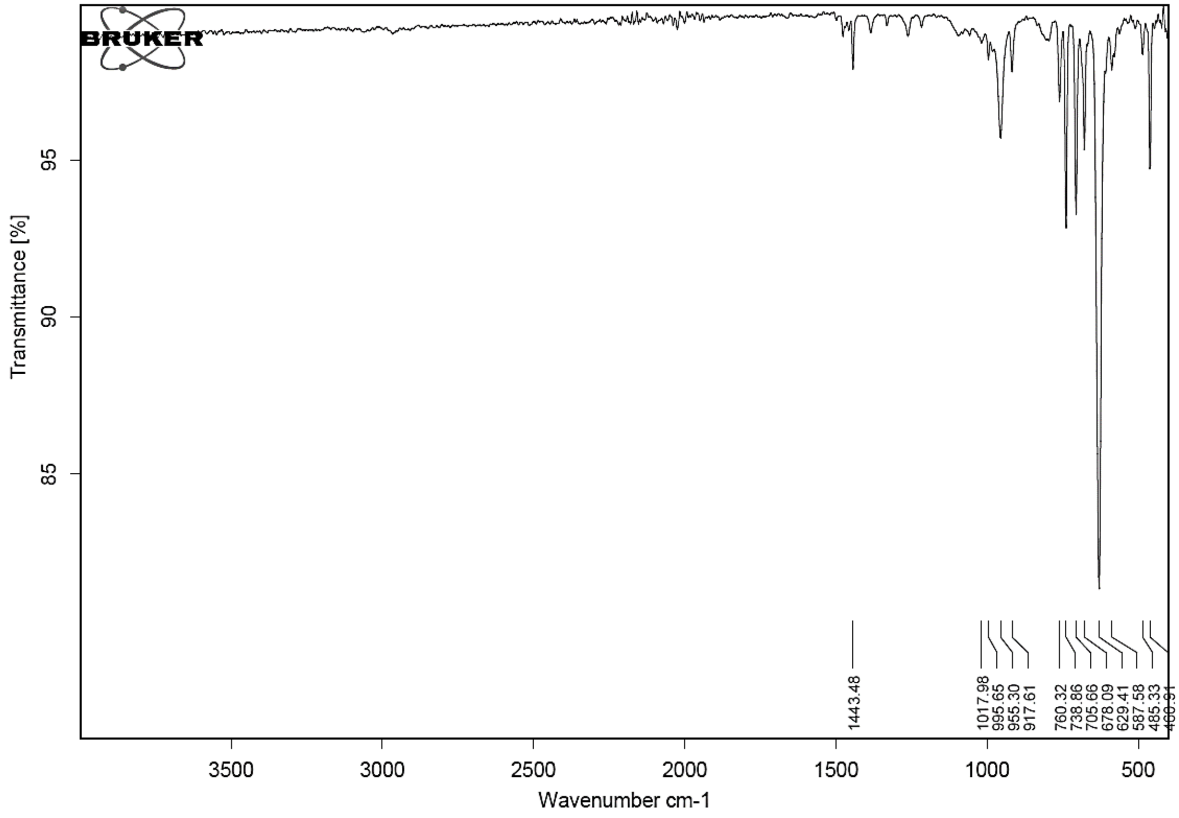

Figure S48: ATR-FTIR spectrum (neat) of **10**.

1.9.2. Preparation of 8

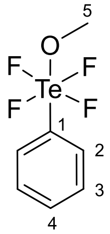

After the alkylation of **7** with methyl iodide was carried out according to the general procedure, the volatiles were removed *in vacuo* at 0 °C. After purification of the residue by column chromatography (SiO_2, ethyl acetate and *n*-hexane (1:8), R_f = 0.51), the product **8** was obtained as a colorless oil.

^{1}H NMR (300 MHz, 300 K, C_6D_6): δ = 7.67–7.60 (m, 2H, H-2), 6.78–6.66 (m, 3H, H-3, H-4), 3.83 (p, $^{4}J_{HF}$ = 1.7 Hz, 3H, H-5).

^{13}C{^{1}H} NMR (75 MHz, 300 K, C_6D_6): δ = 133.6 (C-3), 130.5–130.4 (m, C-2, C-4), 57.9–57.8 (m, C-5). Note: The resonance for C-1 was not detected.

^{19}F NMR (376 MHz, 300 K, C_6D_6): δ = -57.8 (s, $^{1}J_{125TeF}$ = 3526 Hz).

^{125}Te{^{1}H} NMR (95 MHz, 300 K, C_6D_6): δ = 729.3 (p, $^{1}J_{TeF}$ = 3526 Hz).

HRMS (ESI, negative): m/z calc. for $[C_7H_8F_3O_2Te]^-$ ([**8**-F+O]$^-$) 310.9544, found: 310.9545.

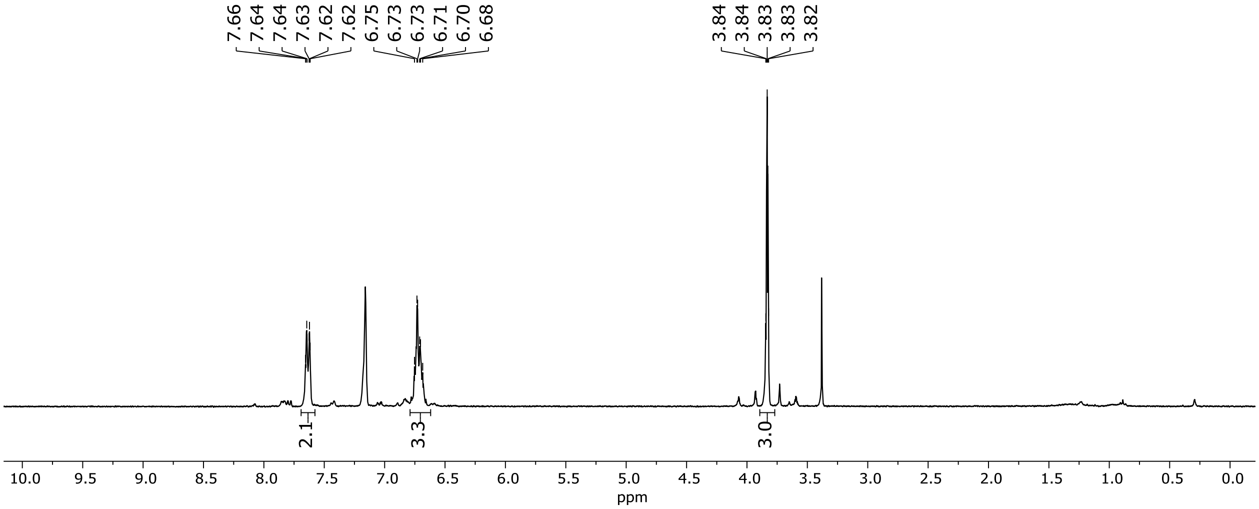

Figure S49: ^{1}H NMR spectrum (C_6D_6, 300 MHz) of **8**.

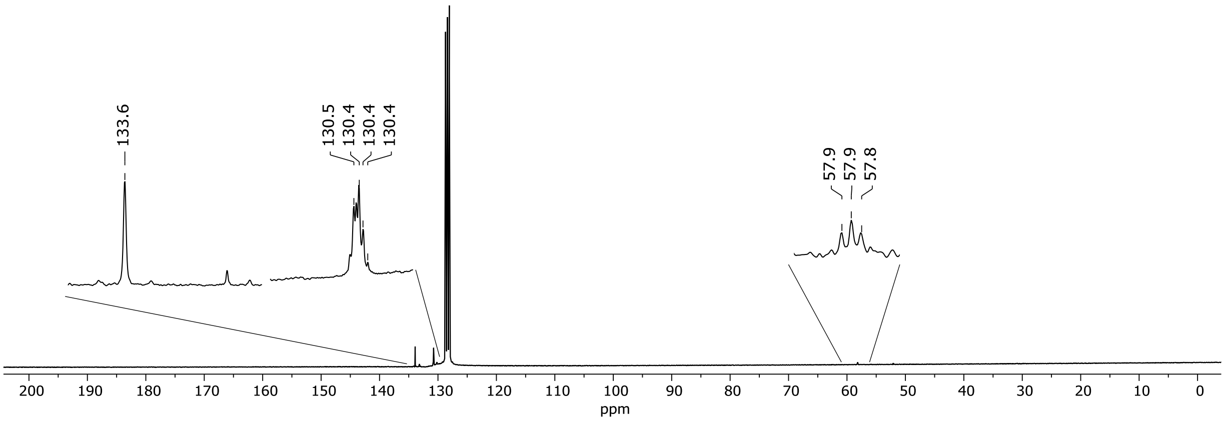

Figure S50: ^{13}C{^{1}H} NMR spectrum (C_6D_6, 75 MHz) of **8**.

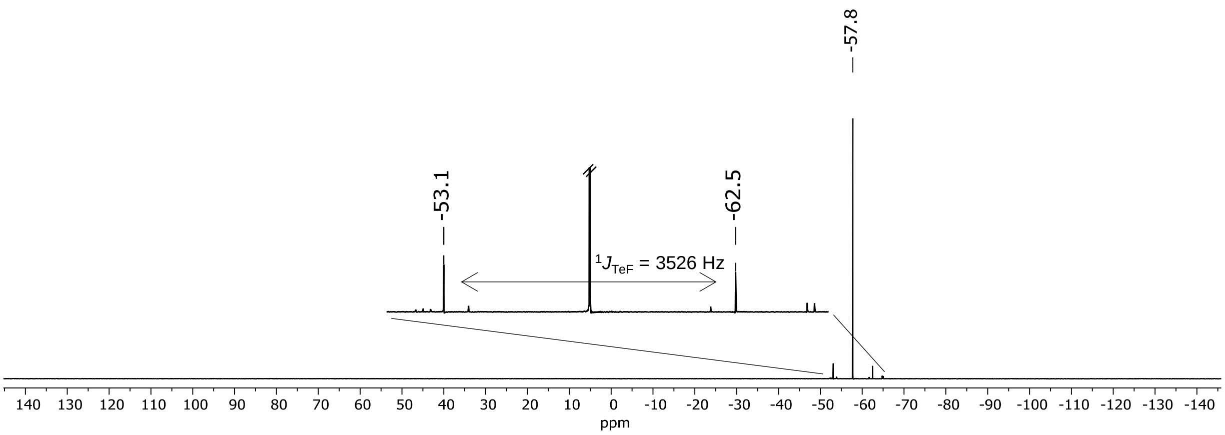

Figure S51: ^{19}F NMR spectrum (C_6D_6, 376 MHz) of **8**.

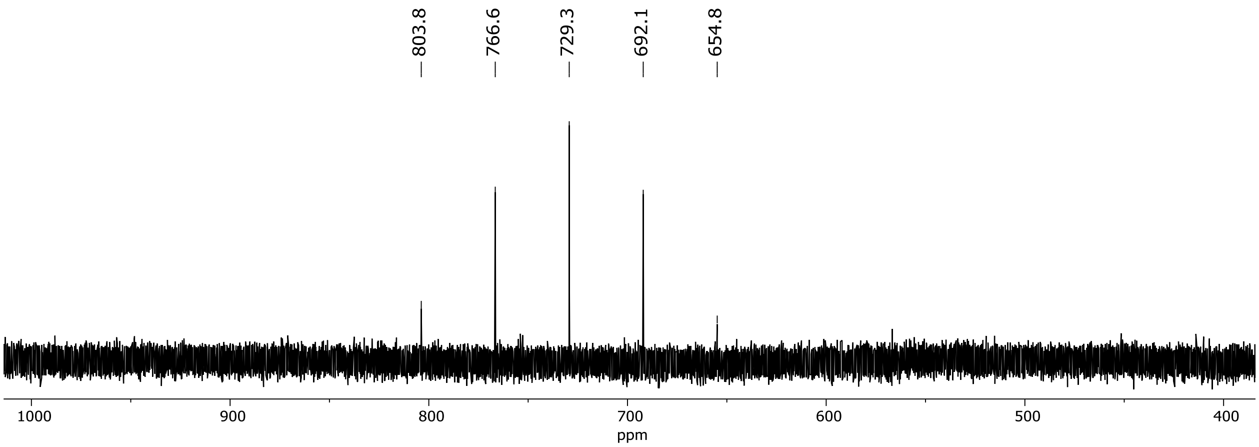

Figure S52: ^{125}Te{^{1}H} NMR spectrum (C_6D_6, 95 MHz) of **8**.

1.9.3. Preparation of 9

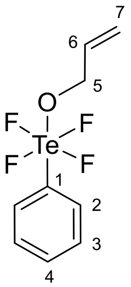

After the alkylation of **7** with allyl iodide was carried out according to the general procedure, the volatiles were removed *in vacuo.* The residue was purified by column chromatography (SiO_2, ethyl acetate and hexane (1:8), Rf = 0.56) to afford **9** as a colorless oil.

^{1}H NMR (400 MHz, 300 K, C_6D_6): δ = 7.65 (d, $^3J_{HH}$ = 7.9 Hz, 2H, H-2), 6.81–6.68 (m, 3H, H-3, H-4), 5.84–5.73 (m, 1H, H-6), 5.18 (d, $^3J_{HH}$ = 17.0 Hz, 1H, H-7a), 4.96, (d, $^3J_{HH}$ = 10.4 Hz, 1H, H-7b), 4.86–4.81 (m, 2H, H-5).

^{13}C{^{1}H} NMR (75 MHz, 300 K, C_6D_6): δ = 147.2–146.6 (m, C-1), 133.8 (C-6), 133.6 (C-3), 130.5–130.4 (m, C-2, C-4), 118.8 (C-7), 71.5 (p, $^3J_{CF}$ = 2.4 Hz, C-5).

^{19}F NMR (376 MHz, 300 K, C_6D_6): δ = -54.6 (s, $^1J_{125TeF}$ = 3535 Hz).

^{125}Te{^{1}H} NMR (126 MHz, 300 K, C_6D_6): δ = 727.4 (p, $^1J_{TeF}$ = 3535 Hz).

HRMS (ESI, negative): m/z calc. for $[C_9H_{10}F_3O_2Te]^-$ ([**9**-F+O]$^-$) 336.9701, found: 336.9702.

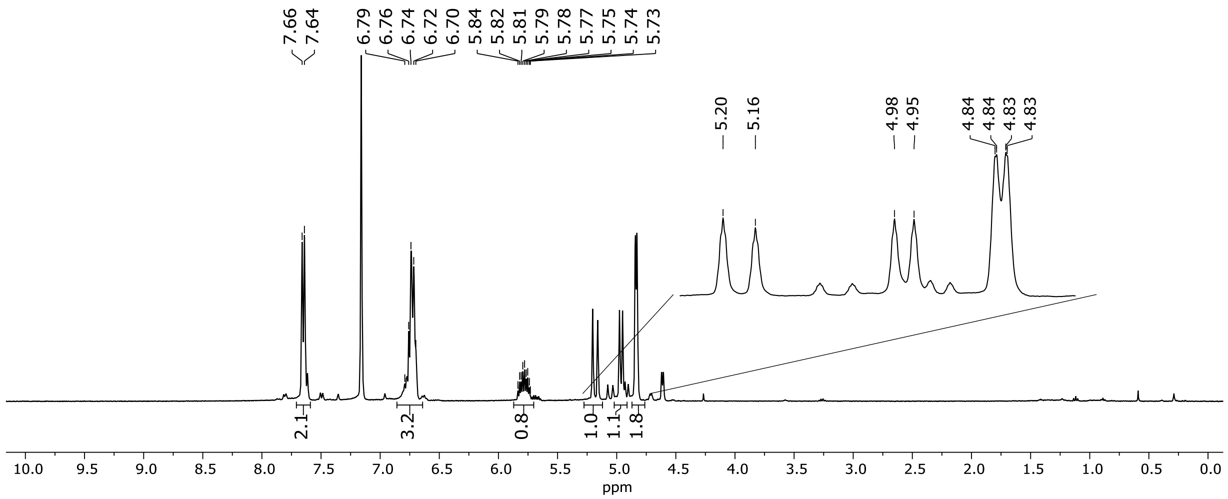

Figure S53: ^{1}H NMR spectrum (C_6D_6, 400 MHz) of **9**.

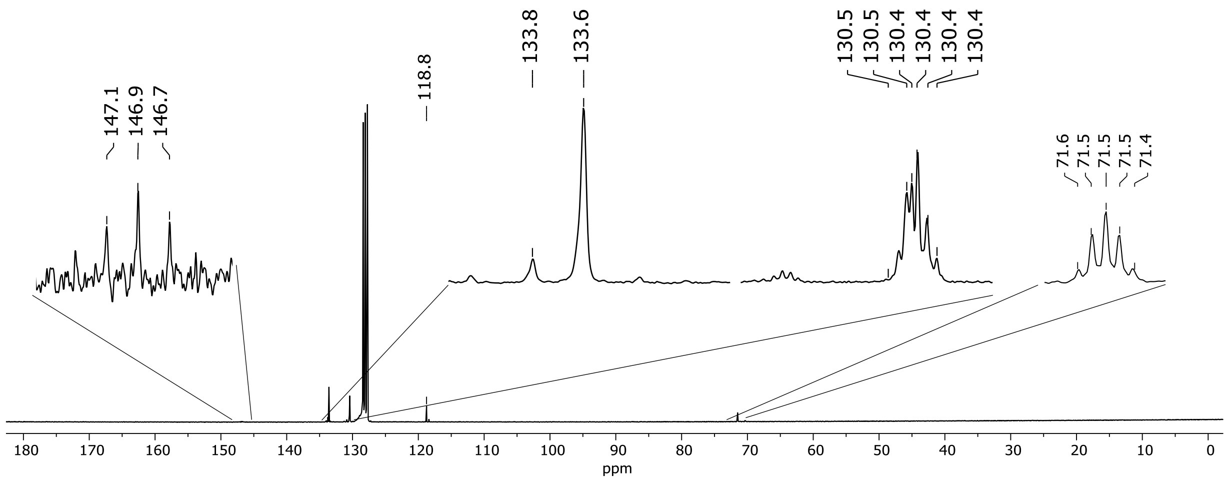

Figure S54: ^{13}C{^{1}H} NMR spectrum (C_6D_6, 75 MHz) of **9**.

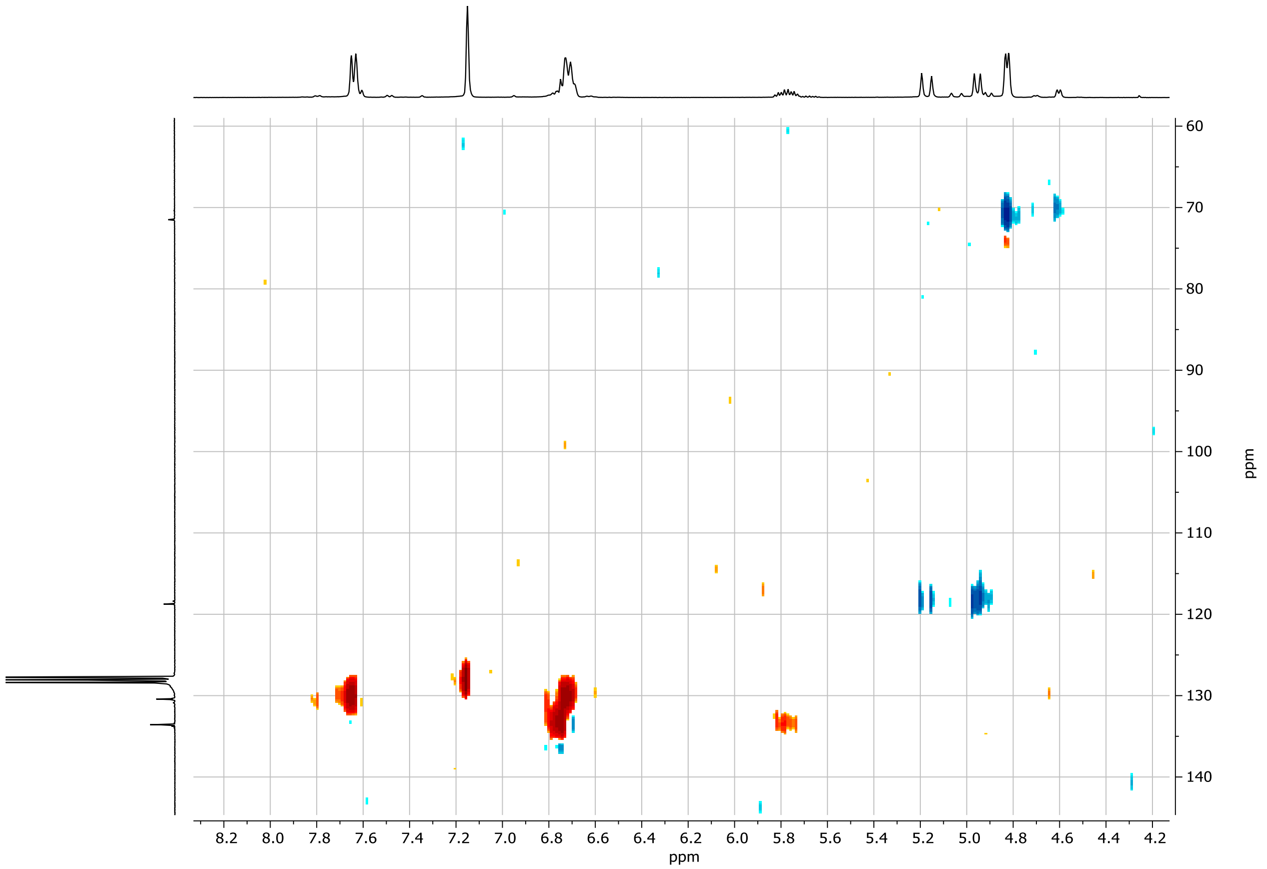

Figure S55: $^{1}H/^{13}C$ HSQC NMR spectrum of **9** in C_6D_6.

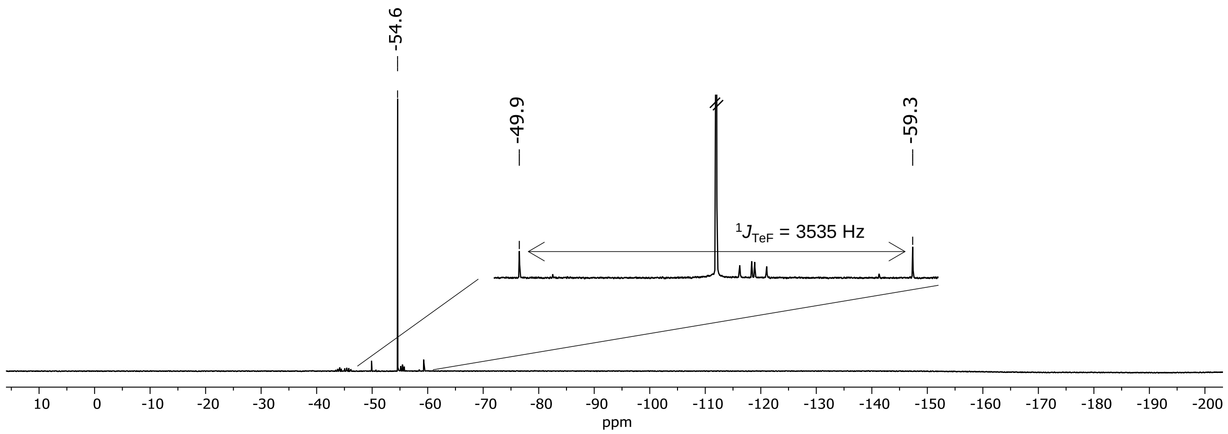

Figure S56: ^{19}F NMR spectrum (C_6D_6, 376 MHz) of **9**.

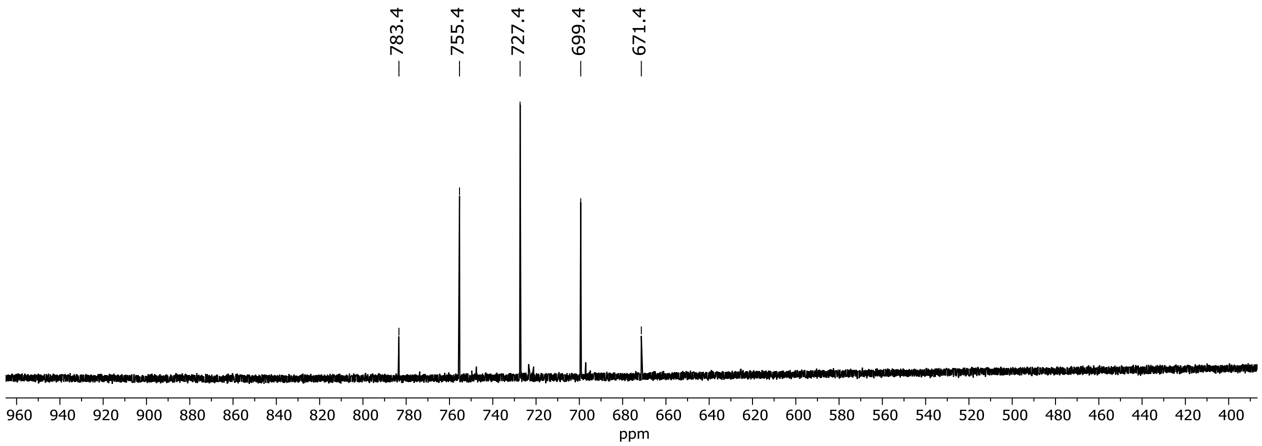

Figure S57: ^{125}Te{^{1}H} NMR spectrum (C_6D_6, 126 MHz) of **9**.

1.10 Reaction of [FP(NsItBu)$_3$][PhTeF$_4$] (5) with alkylation reagents

The $PhTeF_5^-$ salt **5** did not react with allyl iodide but decomposed by using MeOTf or (Et_3O)BF_4 under formation of MeF or EtF, respectively (Figure S58 and Figure S60) and degradation of the fluorophosphonium cation to the PF_6^- anion (Figure S59). The reaction with (Et_3O)BF_4 proceeded slowly at room temperature. After four weeks, single crystals suitable for an X-ray diffraction study were obtained from a trifluorotoluene solution containing **5** and (Et_3O)BF_4 in a ratio of 1:2. The XRD study revealed the molecular structure of the decomposition product **11**, which could not be isolated but decomposed upon prolonged storage of the solution. Analysis of the solution by HRMS-ESI(+) confirmed the formation of **11**: [$C_{31}H_{54}F_3N_9PTe$] (**M**+F)$^+$ m/z = 770.32510 (calc.: 770.32486). The formation of **11** can be rationalized by an initial fluoride abstraction by (Et_3O)BF_4 followed by coordination of the electrophilic $PhTeF_3$ by an N atom of the phosphonium cation leading to the CH insertion of tellurium into a *tert*-butyl group and cleavage of two additional groups by Hofmann elimination.

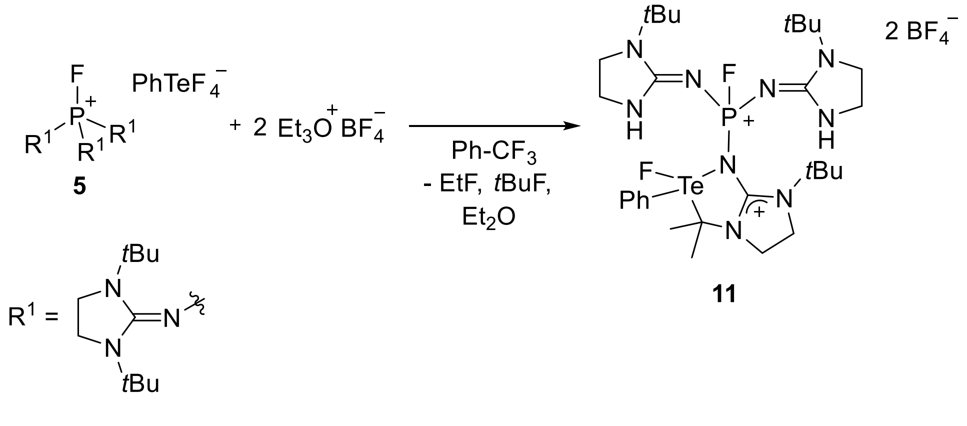

Scheme S2: Reaction of **5** with two equivalents of (Et_3O)BF_4 to give **11**.

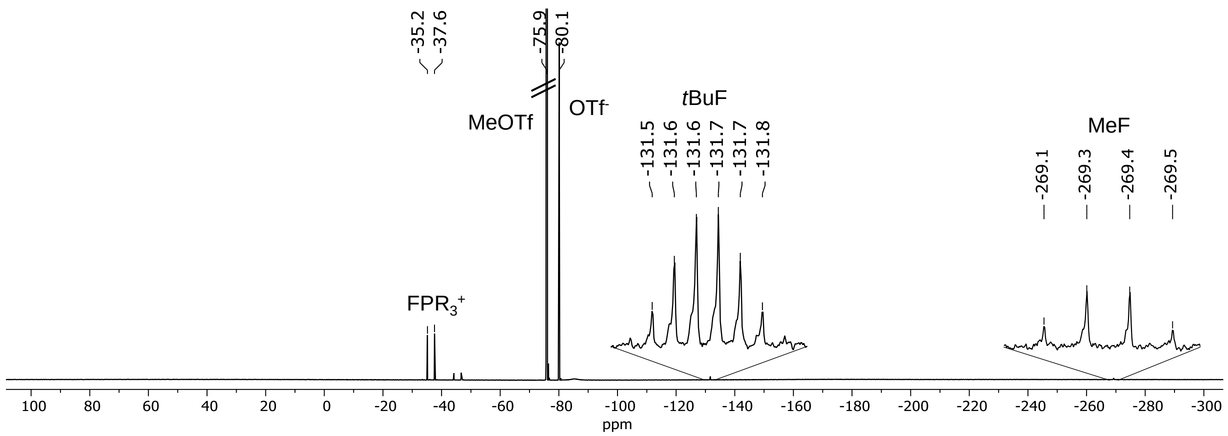

Figure S58: ^{19}F NMR spectrum (CD_2Cl_2, 376 MHz) of the reaction mixture of **5** and MeOTf (excess) after storing the solution for 24 hours at ambient temperature.

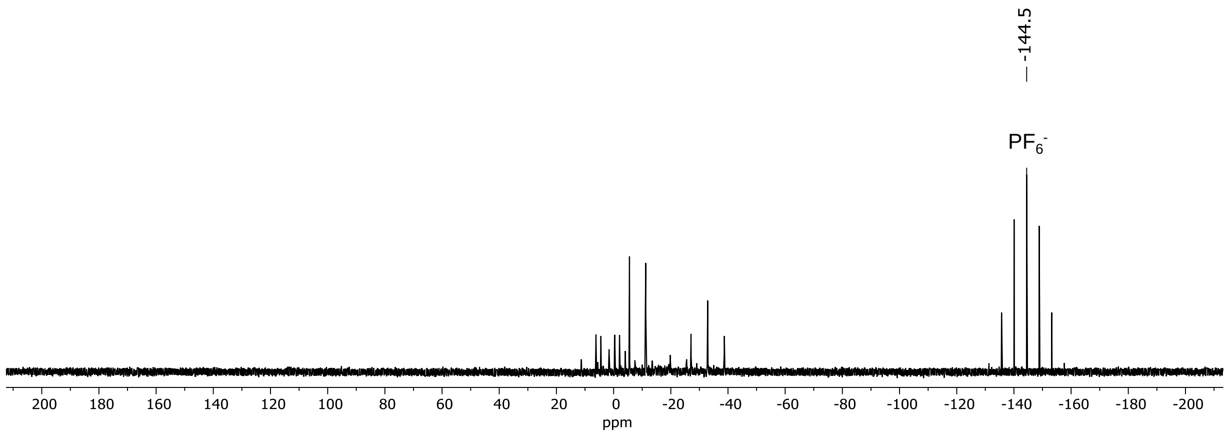

Figure S59: ^{31}P NMR spectrum (CD_2Cl_2, 162 MHz) of the reaction mixture of **5** and MeOTf (excess) after storing the solution for 48 hours at ambient temperature.

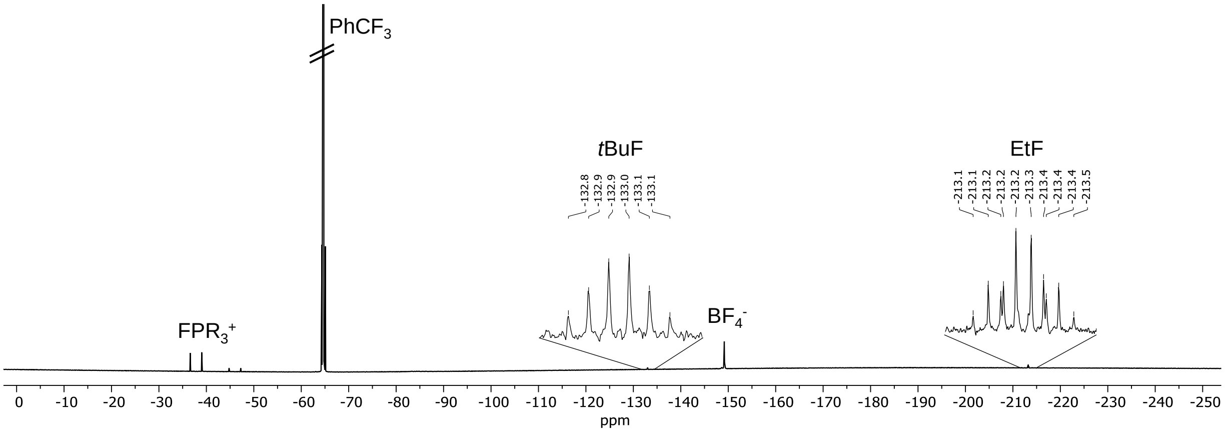

Figure S60: ^{19}F NMR spectrum (α,α,α-trifluorotoluene, 376 MHz) of the reaction mixture of **5** and $Et_3O{\cdot}BF_4$ (2 eq.) after storing the solution for 14 hours at ambient temperature.

1.11 Hydrolysis experiments of $[FP(NsItBu)_3][PhTeF_4]$ (5) and *trans*-$PhTeF_4(OMe)$ (10)

Compound 5: Degassed H_2O (10 eq.) was added to an NMR tube containing **5** (30 mg, 0.03 mmol) in MeCN-*d*3. After 24 hours, broadening of the ^{19}F NMR resonance of the $PhTeF_4^-$ anion was observed. Over a period of 55 days the intensity of the ^{19}F resonance of the $PhTeF_4^-$ anion decreased and a brown precipitate was formed.

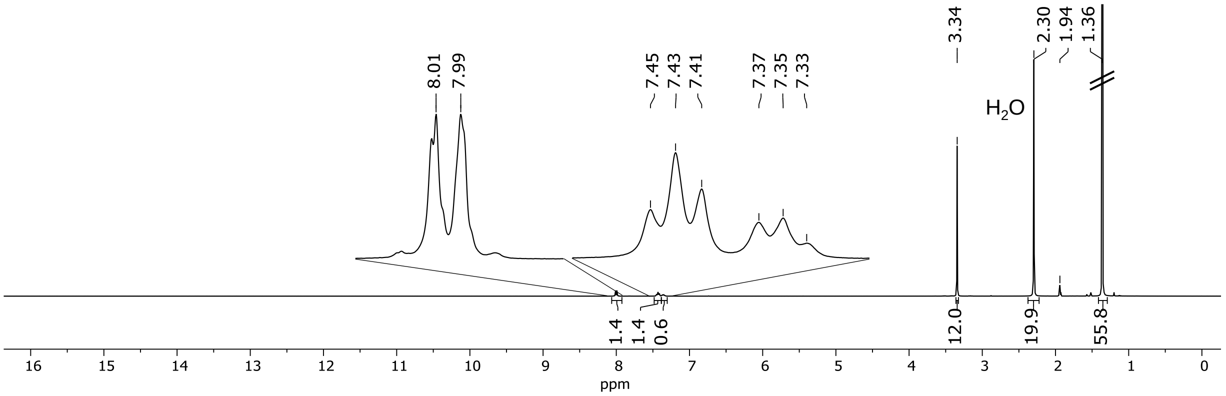

Figure S61: ^{1}H NMR spectrum (MeCN-*d*3, 400 MHz) of **5** 24 hours after the addition of degassed H_2O.

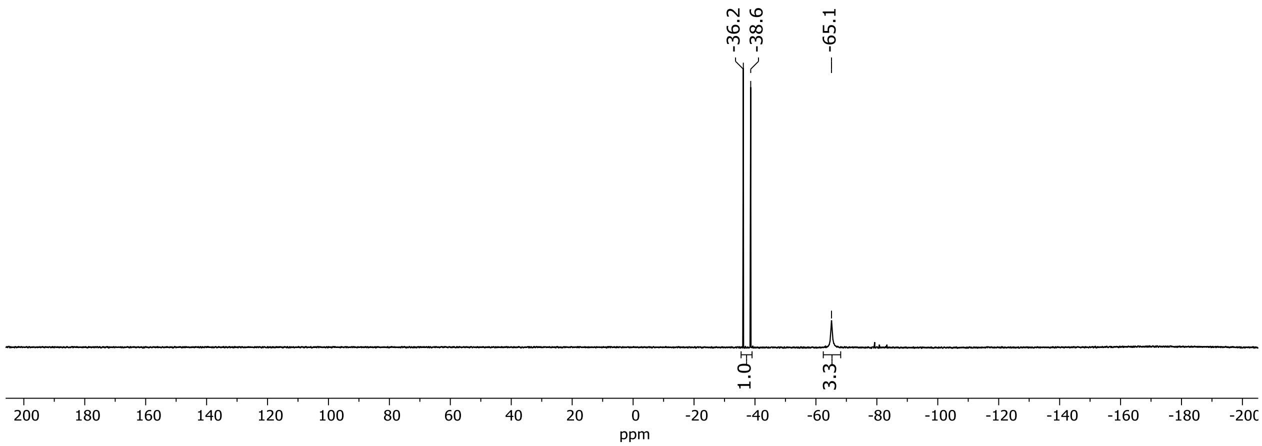

Figure S62: ^{19}F NMR spectrum (MeCN-d_3, 376 MHz) of **5** 24 hours after the addition of degassed H_2O.

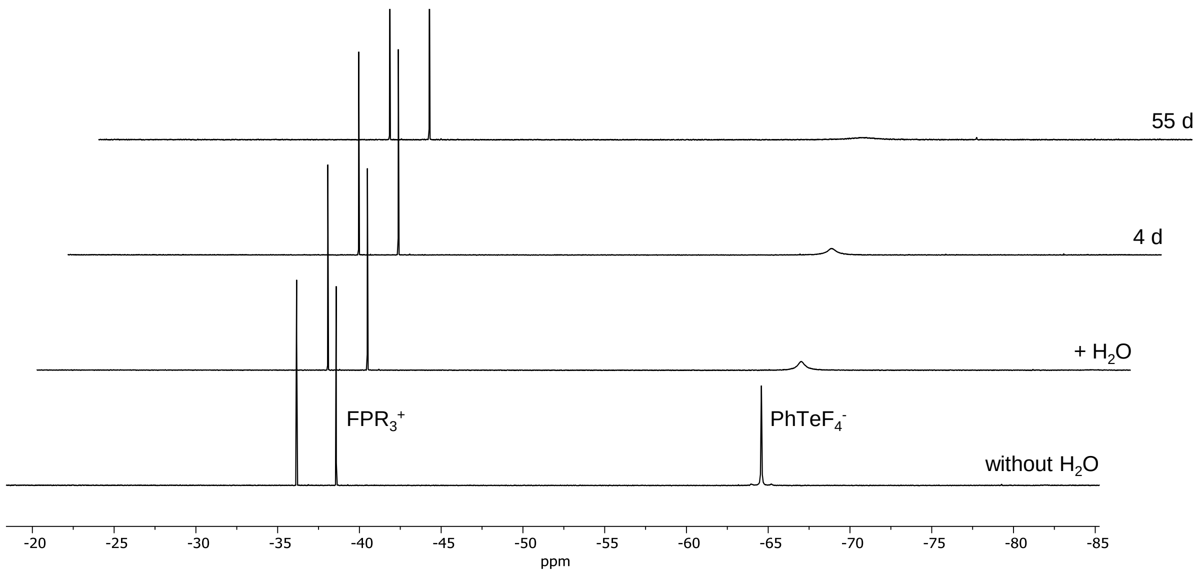

Figure S63: ^{19}F NMR spectra (MeCN-d_3, 376 MHz) of **5** (bottom) and after the addition of degassed H_2O after 24 hours, 4 days and 55 days.

Compound 10: Compound **10** (8 mg, 0.02 mmol) was dissolved in a 10:1 mixture of MeCN/H_2O and the solution was analyzed by ^{19}F NMR spectroscopy. After 3 hours, a new set of signals appeared in the ^{19}F NMR spectrum, indicating that the mono-hydrolysis product was formed in less than 1% (see Figure S64).

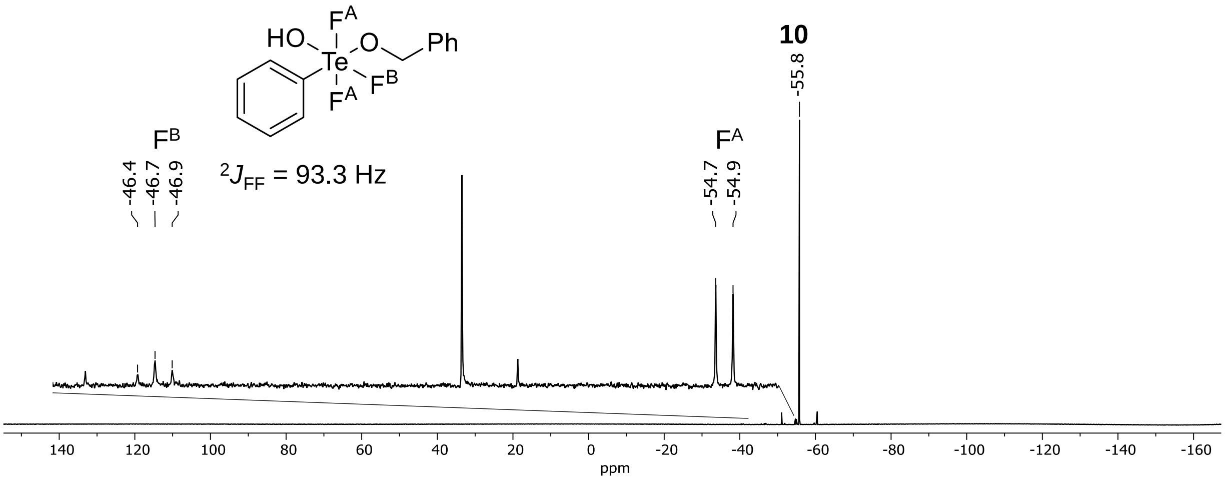

Figure S64: ^{19}F NMR spectrum (MeCN-d_3, 376 MHz) after storing the solution containing **10** in MeCN/H_2O (10:1) for 3 hours at ambient temperature. The putative hydrolysis product is depicted.

1.12 Oxidative fluorination of elemental selenium and tellurium using [FP(NI*i*Pr)$_3$][SF$_5$] (NI*i*Pr = 1,3-diisopropyl-4,5-dimethylimidazolin-2-ylidenamino)

The phosphonium salt [FP(NI*i*Pr)$_3$][SF$_5$], which was synthesized following the reported procedure,[1] and an excess of elemental selenium or tellurium were suspended in THF-d_8. The suspensions were filled into NMR tubes. The sealed tubes were heated for 72 hours at 120 °C (in the case of Se) and at 80 °C (in the case of Te). Besides the fluorinated anions of selenium and tellurium, significant amounts of hexafluorophosphate were detected in both samples (*vide infra*).

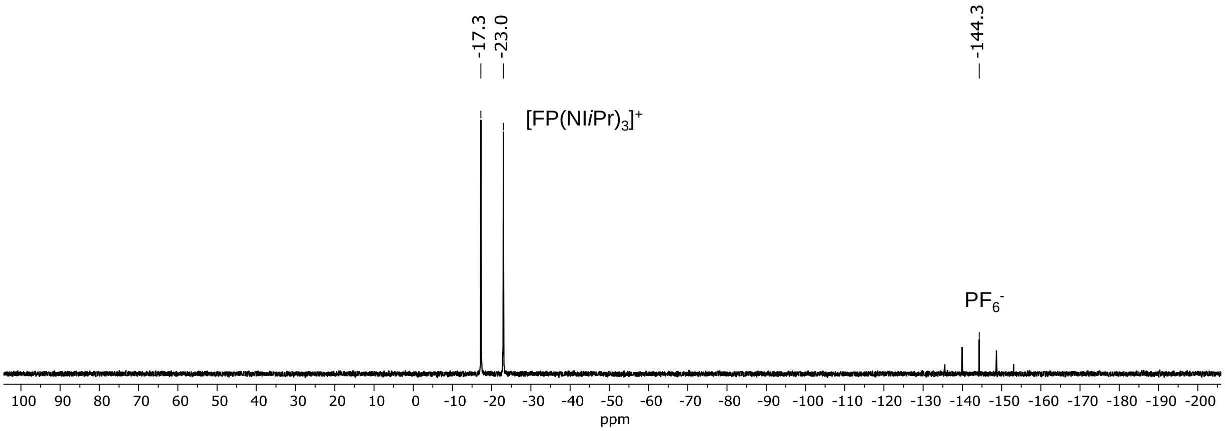

Figure S65: ^{31}P NMR spectrum (THF-d_8, 162 MHz) of the reaction mixture of [FP(NI*i*Pr)$_3$][SF$_5$] with elemental selenium after 72 hours at 120 °C.

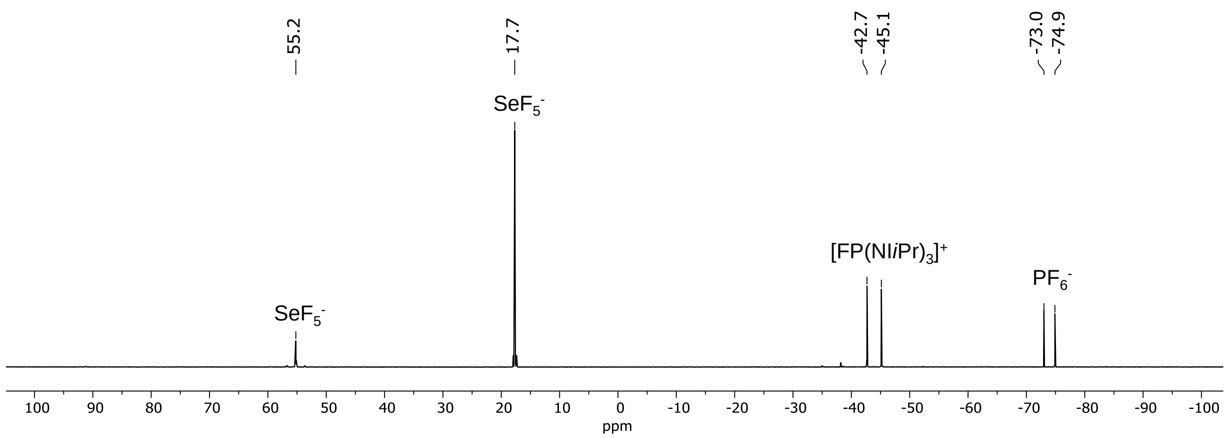

Figure S66: ^{19}F NMR spectrum (THF-d_8, 376 MHz) of the reaction mixture of [FP(NI*i*Pr)$_3$][SF$_5$] with elemental selenium after 72 hours at 120 °C.

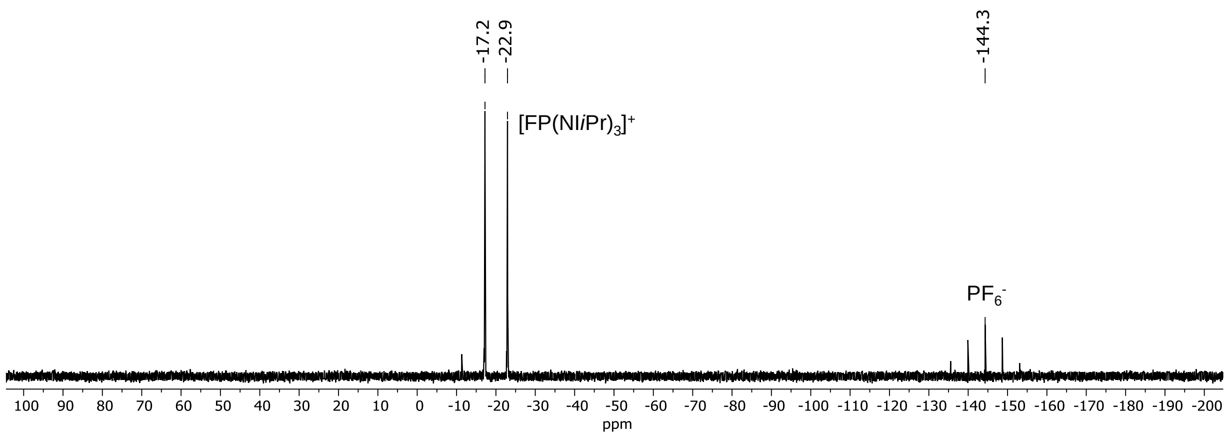

Figure S67: ^{31}P NMR spectrum (THF-d_8, 162 MHz) of the reaction mixture of [FP(NI*i*Pr)$_3$][SF$_5$] with elemental tellurium after 72 hours at 80 °C.

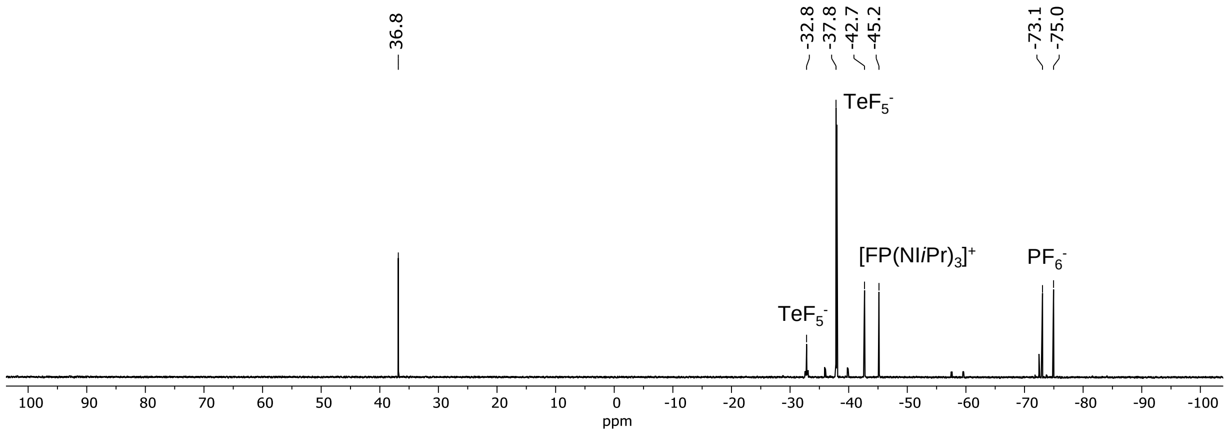

Figure S68: ^{19}F NMR spectrum (THF-d_8, 376 MHz) of the reaction mixture of [FP(NI*i*Pr)$_3$][SF$_5$] with elemental tellurium after 72 hours at 80 °C.

2 X-ray Diffraction Studies

General: Single-crystal X-ray diffraction data were collected on a Bruker AXS and on a Bruker Photon 100 detector using Mo-K$_\alpha$ radiation (λ = 0.71073 Å). Crystals were selected under oil, mounted on nylon loops and then immediately placed in a cold stream of N_2 on a diffractometer. The APEX2 software (for Bruker AXS) and the APEX3 software (for Bruker Photon 100) were used to operate the diffractometers. The data was integrated with SAINT15[5] and corrected for absorption effects based on Gaussian numerical integration and scaled with SADABS.16.[6] Using Olex2,[7] the structures were solved with SIR2004[8] using direct methods or ShelXT[9] with Intrinsic Phasing and refined with the ShelXL[10] refinement package using Least Squares minimization.

Crystallographic data have been deposited with the Cambridge Crystallographic Data Centre as supplementary publication no. CCDC-2180124 (**2**), CCDC-2180128 (**5**), CCDC-2180126 (**6**), CCDC-2180127 (**7**), CCDC-2180125 (**11**). These data can be obtained free of charge via www.ccdc.cam.uk/data_request/cif (or from the CCDC, 12 Union Road, Cambridge CB2 1EZ, UK; fax: (+44) 1223-336-033; or deposit@ccdc.cam.ac.uk).

2.1 Crystal structure data of compound 2

Single crystals were obtained by slowly cooling an 80 °C hot, saturated THF solution of **2**. A single-crystal X-ray structure analysis revealed that **2** crystallizes in the monoclinic space group $P2_1/n$. The asymmetric unit contains one molecule of the cation and the anion of salt **2**. The SF_5 anion is partially disordered over two positions (occupancy: 59:41).

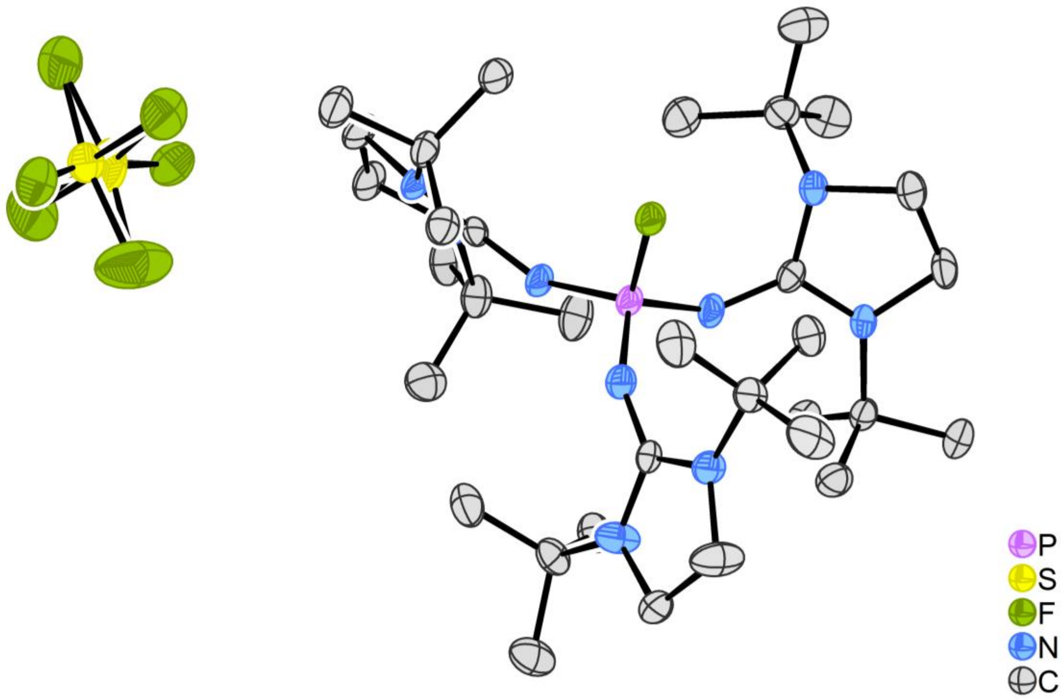

Figure S69*:* The asymmetric unit of the crystal structure of **2** with thermal ellipsoid plot at the 50% levels of probability. Hydrogen atoms are omitted for clarity.

Table S4: Crystal data and structure refinement for **2**.

CCDC number	2180124	ρ_{calc} g/cm^3	1.305
Empirical formula	$C_{33}H_{66}F_6N_9PS$	μ/mm^{-1}	0.190
Formula weight	765.97	F(000)	1648.0
Temperature/K	100	Crystal size/mm^3	0.429 × 0.352 × 0.28
Crystal system	monoclinic	Radiation	MoKα (λ = 0.71073)
Space group	$P2_1/n$	2Θ range for data collection	3.754 to 53.052
a/Å	16.5555(8)	Index ranges	-20 ≤ h ≤ 20, -19 ≤ k ≤ 18, -20 ≤ l ≤ 20
b/Å	15.2547(9)	Reflections collected	30408
c/Å	16.7326(9)	Independent reflections	7962 [R_{int} = 0.0647, R_{sigma} = 0.0547]
α/°	90	Data/restraints/parameters	7962/12/488
β/°	112.656(3)	Goodness-of-fit on F^2	1.036
γ/°	90	Final R indexes [I>=2σ (I)]	R_1 = 0.0605, wR_2 = 0.1537
Volume/Å^3	3899.7(4)	Final R indexes [all data]	R_1 = 0.0846, wR_2 = 0.1671
Z	4	Largest diff. peak/hole / e Å^{-3}	0.76/-0.43

2.2 Crystal structure data of compound 5

Single crystals were obtained by vapor diffusion of diethyl ether into a saturated THF solution of **5**. Compound **5** crystallizes in the orthorhombic space group *Pbca.* The asymmetric unit contains one molecule of the cation and the anion of salt **5**. The fluorophosphonium cation is disordered over two positions (occupancy: 66:34).

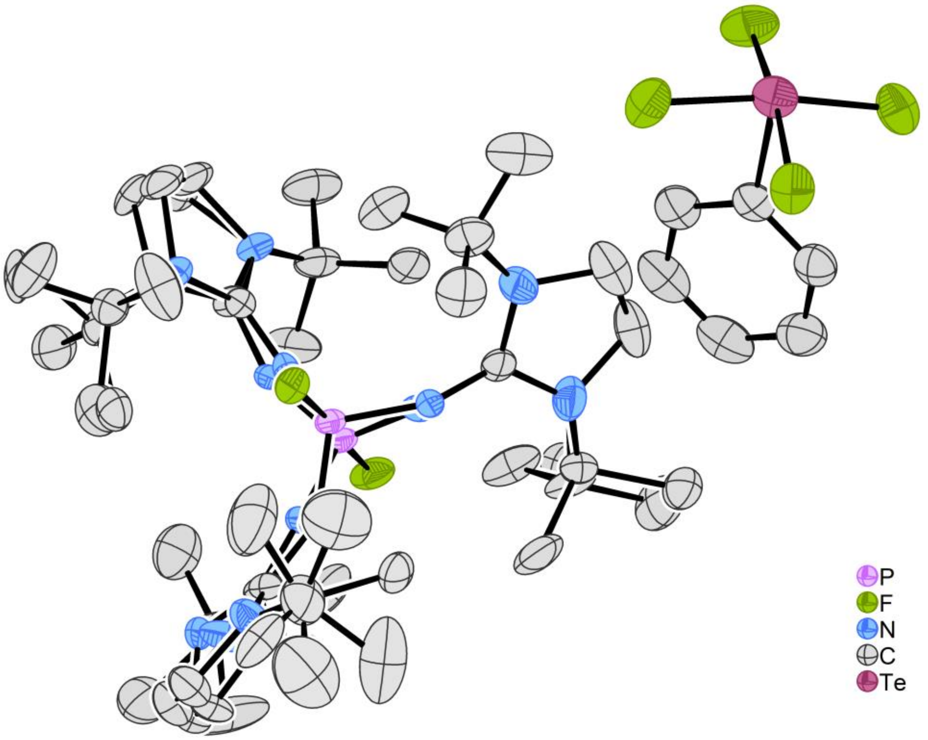

Figure S70: The asymmetric unit of the crystal structure of **5** with thermal ellipsoid plot at the 50% levels of probability. Hydrogen atoms are omitted for clarity.

Table S5: Crystal data and structure refinement for **5**.

CCDC number	2180128	ρ_{calc} g/cm^3	1.354
Empirical formula	$C_{39}H_{71}F_5N_9PTe$	μ/mm^{-1}	0.754
Formula weight	919.61	*F*(000)	3840.0
Temperature/K	100	Crystal size/mm^3	0.6 × 0.444 × 0.148
Crystal system	orthorhombic	Radiation	MoKα (λ = 0.71073)
Space group	*Pbca*	2Θ range for data collection	3.5 to 56.784
a/Å	19.4313(5)	Index ranges	$-26 \leq h \leq 25$, $-21 \leq k \leq 21$, $-38 \leq l \leq 38$
b/Å	15.9729(4)	Reflections collected	135455
c/Å	29.0592(8)	Independent reflections	11270 [R_{int} = 0.0576, R_{sigma} = 0.0283]
α/°	90	Data/restraints/parameters	11270/102/759
β/°	90	Goodness-of-fit on F^2	1.199
γ/°	90	Final R indexes [I>=2σ (I)]	R_1 = 0.0777, wR_2 = 0.1498
Volume/Å^3	9019.2(4)	Final R indexes [all data]	R_1 = 0.0835, wR_2 = 0.1522
Z	8	Largest diff. peak/hole / e Å^{-3}	2.56/-1.82

2.3 Crystal structure data of compound 6

Single crystals were obtained by liquid/liquid diffusion of diethyl ether into a saturated THF solution of **6**. A single-crystal X-ray structure analysis revealed that **6** crystallizes in the orthorhombic space group *Pbca*. The asymmetric unit contains one molecule of the cation and the anion of the salt **6**. Both the P–F moiety and the backbone of two substituents in the fluorophosphonium cation are disordered over two positions (occupancy: 74:26 (P–F)/ 58:42 (Substituent A) / 71:29 (Substituent B)).

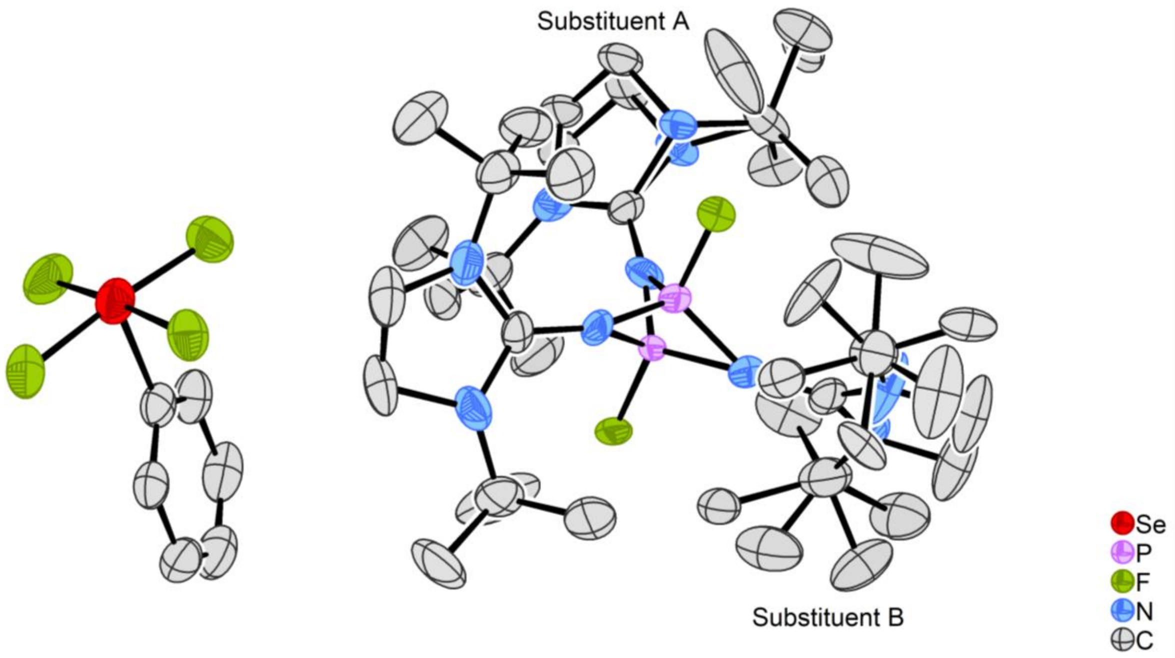

Figure S71: The asymmetric unit of the crystal structure of **6** with thermal ellipsoid plot at the 50% levels of probability. Hydrogen atoms are omitted for clarity.

Table S6: Crystal data and structure refinement for **6**.

CCDC number	2180126	ρ_{calc} g/cm^3	1.299
Empirical formula	$C_{39}H_{71}F_5N_9PSe$	μ/mm^{-1}	0.938
Formula weight	870.97	*F*(000)	3696.0
Temperature/K	100.0	Crystal size/mm^3	0.43 × 0.319 × 0.268
Crystal system	orthorhombic	Radiation	MoKα (λ = 0.71073)
Space group	*Pbca*	2*Θ* range for data collection	4.35 to 51.634
a/Å	19.3957(18)	Index ranges	-23 ≤ h ≤ 23, -19 ≤ k ≤ 19, -35 ≤ l ≤ 35
b/Å	15.9132(15)	Reflections collected	85465
c/Å	28.865(3)	Independent reflections	8501 [R_{int} = 0.0580, R_{sigma} = 0.0328]
α/°	90	Data/restraints/parameters	8501/0/612
β/°	90	Goodness-of-fit on F^2	1.230
γ/°	90	Final R indexes [I>=2*σ* (I)]	R_1 = 0.1015, wR_2 = 0.1919
Volume/Å^3	8909.1(14)	Final R indexes [all data]	R_1 = 0.1079, wR_2 = 0.1946
Z	8	Largest diff. peak/hole / e Å^{-3}	1.00/-0.95

2.4 Crystal structure data of compound 7

Single crystals were obtained by vapor diffusion of diethyl ether into saturated DCM solution of **7** at -40 °C. **7** crystallizes in the monoclinic space group $P2_1/c$. The asymmetric unit contains one molecule of the cation and the anion of the salt **7** and one DCM molecule. Both the fluorophosphonium cation and the DCM molecule are disordered over two positions (occupancy: 50:50 (fluorophosphonium)/ 77:23 (DCM)).

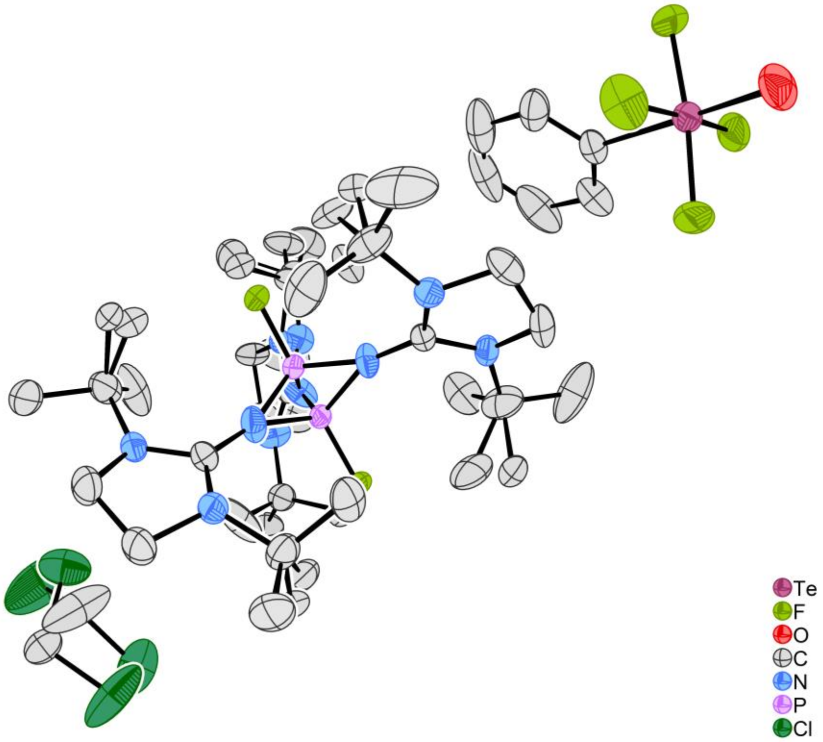

Figure S72: The asymmetric unit of the crystal structure of **7**·DCM with thermal ellipsoid plot at the 50% levels of probability. Hydrogen atoms are omitted for clarity.

Table S7: Crystal data and structure refinement for **7**·DCM.

CCDC number	2180127	ρ_{calc} g/cm^3	1.360
Empirical formula	$C_{40}H_{73}Cl_2F_5N_9OPTe$	μ/mm^{-1}	0.794
Formula weight	1020.54	F(000)	2120.0
Temperature/K	173.0	Crystal size/mm^3	0.21 × 0.19 × 0.09
Crystal system	monoclinic	Radiation	MoKα (λ = 0.71073)
Space group	$P2_1/c$	2Θ range for data collection	4.272 to 52.152
a/Å	14.4175(7)	Index ranges	$-17 \le h \le 17, -26 \le k \le 25, -20 \le l \le 20$
b/Å	21.1507(10)	Reflections collected	138776
c/Å	16.3527(9)	Independent reflections	9862 [R_{int} = 0.0362, R_{sigma} = 0.0154]
α/°	90	Data/restraints/parameters	9862/12/702
β/°	91.402(2)	Goodness-of-fit on F^2	1.077
γ/°	90	Final R indexes [I>=2σ (I)]	R_1 = 0.0396, wR_2 = 0.0936
Volume/Å^3	4985.1(4)	Final R indexes [all data]	R_1 = 0.0447, wR_2 = 0.0970
Z	4	Largest diff. peak/hole / e Å^{-3}	1.65/-1.22

2.5 Crystal structure data of compound 11

Single crystals were obtained by storing a reaction mixture of **5** and $Et_3O{\cdot}BF_4$ in trifluorotoluene for four weeks. A single-crystal X-ray structure analysis revealed that **11** crystallizes in the triclinic space group $P\bar{1}$. The asymmetric unit contains one molecule of **11**$(BF_4)_2$ and 1.5 triflourotoluene molecules. **11** as well as one trifluorotoluene molecule are disordered over two positions respectively (occupancy: 60:40 (**11**) / 50:50 (triflourotoluene)).

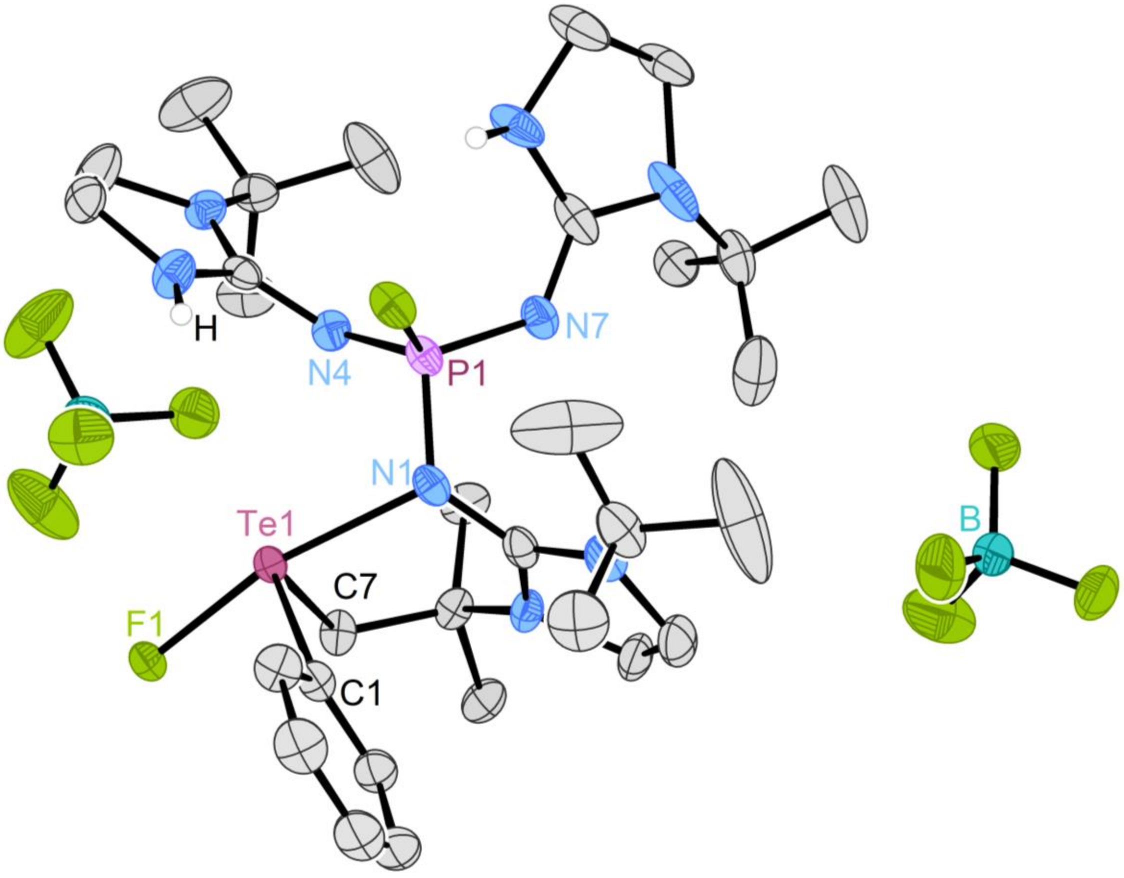

Figure S73: Molecular structure of **11**. Hydrogen atoms except those at the N atoms are omitted for clarity; thermal ellipsoids are set at 50% probability. Selected bond lengths [Å] and angles [º]: Te1–F1 1.999(1), Te1–N1 2.260(2), Te1–C7 2.111(2), Te–C1 2.107(2), P1–N1 1.637(2), P1–N4 1.560(2), P1–N7 1.578(2), N1-Te1-F1 166.99(6), C7-Te1-C1 99.45(9), P1-N1-Te1 111.20(10).

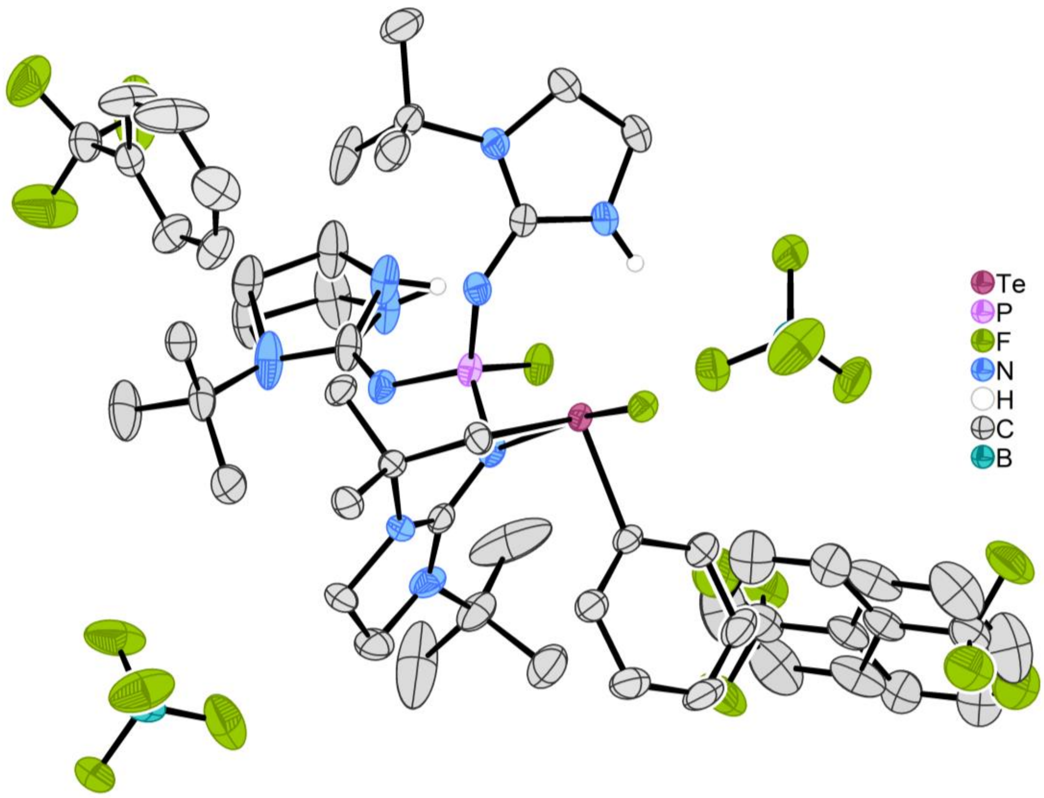

Figure S74: The asymmetric unit of the crystal structure of **11** with thermal ellipsoid plot at the 50% levels of probability. Hydrogen atoms, except in the two protic imidazolidinium moieties, are omitted for clarity.

Table S8. Crystal data and structure refinement for **11**.

CCDC number	2180125	ρ_{calc} g/cm^3	1.525
Empirical formula	$C_{83}H_{123}B_4F_{29}N_{18}P_2Te_2$	μ/mm^{-1}	0.724
Formula weight	2284.37	*F*(000)	1166.0
Temperature/K	100	Crystal size/mm^3	0.329 × 0.167 × 0.159
Crystal system	triclinic	Radiation	MoKα (λ = 0.71073)
Space group	$P\bar{1}$	2Θ range for data collection	3.176 to 56.758
a/Å	11.6624(4)	Index ranges	-15 ≤ h ≤ 15, -19 ≤ k ≤ 19, -21 ≤ l ≤ 21
b/Å	14.4395(4)	Reflections collected	38569
c/Å	16.3872(5)	Independent reflections	12427 [Rint = 0.0403, Rsigma = 0.0410]
α/°	65.291(2)	Data/restraints/parameters	12427/86/740
β/°	83.786(2)	Goodness-of-fit on F^2	1.034
γ/°	87.796(2)	Final R indexes [I>=2σ (I)]	R1 = 0.0345, wR2 = 0.0843
Volume/Å^3	2492.13(14)	Final R indexes [all data]	R1 = 0.0401, wR2 = 0.0884
Z	1	Largest diff. peak/hole / e Å^{-3}	1.05/-0.77

3 Computational Data

The geometry optimizations and frequency calculations were performed with *Gaussian 09*,[11] using the wB97XD range separated hybrid functional[12]. Triple zeta basis sets were used in the calculations: aug-cc-pVTZ for C, O, F, H, [13] while selenium and tellurium were described with aug-cc-pVTZ-PP that provides additional diffuse functions and additional pseudopotentials.[14] The absence of imaginary frequencies confirmed that each optimized structure is at a local minimum (for SeF_3Ph an optimized structure with one imaginary frequency of -22.4 cm^{-1} was used). Such low imaginary frequencies are of minor concern as they occur due to numerical instabilities of the algorithm.

To shed light on the dynamic behavior of **$PhSeF_4^-$** in the NMR, a second conformation with one F ligand being the apex of the square pyramid rather than the phenyl ring was investigated. Structure optimization were performed with the wB97XD3 and the def2-TZVP basis set[15] in implicit THF modelled by a polarizable continuum to account for bulk solvent effects. To search for the transition state between the two conformers, the nudged electric band (NEB) method as implemented in ORCA[16] was used obtain an initial guess structure with wB97XD3/def2-TZVP/THF. The final transition state was converged by eigenvector following and identified by one imaginary frequency that coincides with the reaction coordinates. A second imaginary frequency with a very low wave number < 12 cm^{-1} was also found (see above).

Final single point energies of these structures were calculated coupled cluster theory in the domain based local pair-natural orbital singles- and doubled coupled cluster with perturbative triples corrections (DLPNO-CCSD(T))[17,18] and the def2-TZVP basis set in implicitly modelled THF.

To check whether dissociation of F^- from **$PhSeF_4^-$** and reassociating is responsible for the observed dynamics in the NMR, the dissociation of F^- was modelled as a (hypothetical) transfer reaction to HF to yield FHF^- mimicking the presence of a potential acceptor.

As the dissociation of F- was monotonously uphill in electronic energy, a method established by Baik et al. was used that estimates the free energy of transition state for such reactions by taking into account the onset of translational and rotational entropy upon dissociation.[19]

Table S9: Calculated energy differences for the two conformers of **$PhSeF_4^-$** and the transition state as well as for F- abstraction from **$PhSeF_4^-$** by HF. Values in kJ mol^{-1}. Thermodynamic corrections to the DLPNO-CCSD(T) energies were obtained at wB97XD3/def2-TZVP/THF.

Structure	Method/Basis Set	ΔE	ΔH_{298}	ΔG_{298}
**$PhSeF_4^-$** conformation 1	wB97XD3/def2-TZVP/THF	0.0	0.0	0.0
**TS**	wB97XD3/def2-TZVP/THF	120.9	116.4	118.8
**$PhSeF_4^-$** conformation 2	wB97XD3/def2-TZVP/THF	70.9	68.7	71.8
**$PhSeF_4^-$** conformation 1	DLPNO-CCSD(T)/def2-TZVP/THF	0.0	0.0	0.0
**TS**	DLPNO-CCSD(T)/def2-TZVP/THF	120.9	116.4	118.9
**$PhSeF_4^-$** conformation 2	DLPNO-CCSD(T)/def2-TZVP/THF	67.9	65.6	68.7
**[$PhSeF_4^-$ +HF]**[a)]	wB97XD3/def2-TZVP/THF	0.0	0.0	0.0
**TS**	wB97XD3/def2-TZVP/THF	-	-	1
**[$PhSeF_3$+HFH^-]**[b)]	wB97XD3/def2-TZVP/THF	-	-	-7
**$PhSeF_3$+HFH^-**	wB97XD3/def2-TZVP/THF	64.1	46.9	20.7
**[$PhSeF_4^-$ +HF]**[a)]	DLPNO-CCSD(T)/def2-TZVP/THF	0.0	0.0	0.0
**$PhSeF_3$+HFH^-**	DLPNO-CCSD(T)/def2-TZVP/THF	59.0	41.8	15.6

[a)] Considered as encounter complex.

[b)] Only a minimum in free energy, estimated from Figure S75.

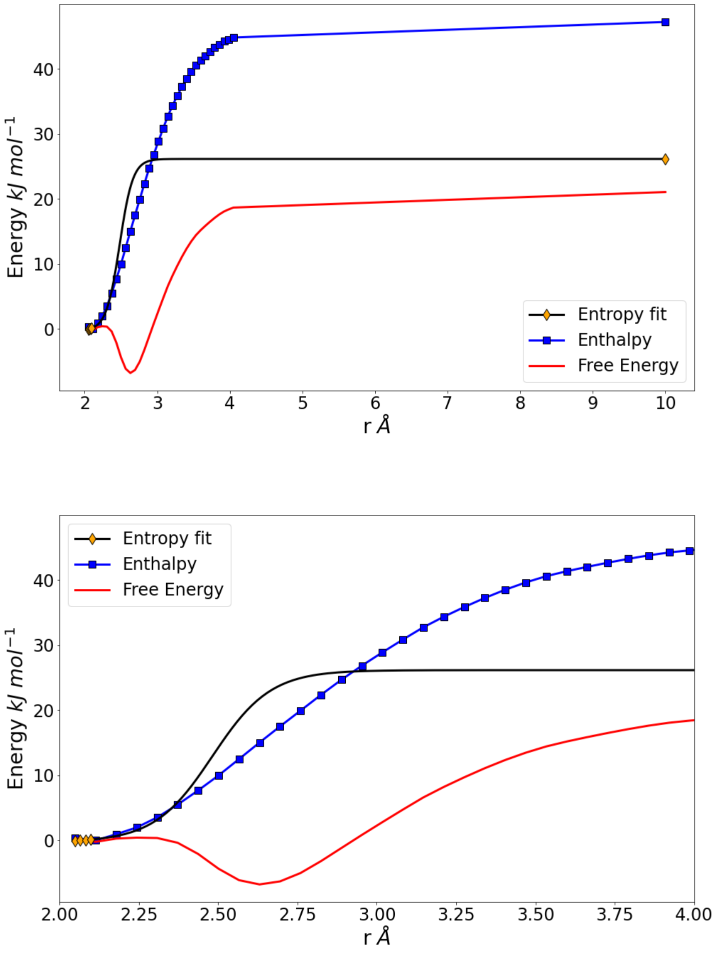

Figure S75: Top: Estimated free energy plotted against the Se-F bond length in $PhSeF_4^-$ for the reaction [$PhSeF_4^-$+HF] to $PhSeF_3$ + FHF^-. The free energy is obtained by taking into account the onset of entropy upon dissociation of the ligand. Bottom: Zoom of the estimated free energy surface around the intermediate.

The Fluoride Ion Affinities (FIA) in the gas phase of selected Lewis acids in the gas phase were calculated according to the procedure of Christe *et al.* using the FIA of COF_2 (experimental value in the gas phase: 209 kJ/mol).[20]

$$CF_3O^- + A \rightarrow COF_2 + AF^-$$

Table S10: Calculated Fluoride Ion Affinity (FIA) using COF_2 as the reference.

Lewis Acid	Gas Phase FIA [kJ·mol^{-1}]
TeF_4	350
TeF_3Ph	302
SeF_4	298
SeF_3Ph	256
BF_3	334
PF_5	373

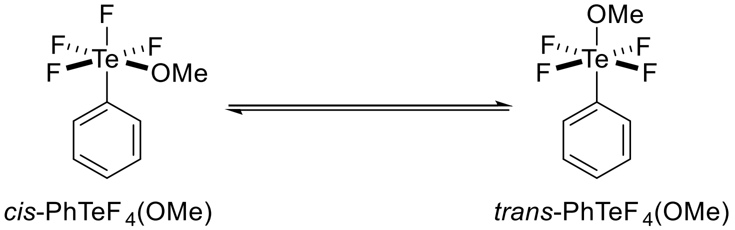

Scheme S3: Calculated *cis*- and *trans*-isomer of $PhTeF_4(OMe)$ (**8**).

As the energy difference between the two $PhTeF_4(OMe)$ (**8**) isomers is rather small, reference calculations with DLPNO-CCSD(T)[18,19] were performed in the gas phase and in implicit acetonitrile. Thermodynamic corrections were taken from the DFT calculations (wB97XD/aug-cc-pVTZ(-PP)). Results are listed in Table S11.

Table S11: Calculated energy differences between the *cis*- and *trans*-isomer of $PhTeF_4(OMe)$ (**8**) for various quantum chemical methods, in the gas phase and in acetonitrile, modelled as implicit solvent. Values in kJ/mol.

Method/Basis Set	*cis*- $PhTeF_4(OMe)$ → *trans*- $PhTeF_4(OMe)$		
	ΔE	ΔH_{298}	ΔG_{298}
wB97XD/aug-cc-pVTZ(-PP)/gas phase	-1.7	-1.9	-1.9
wB97XD/aug-cc-pVTZ(-PP)/MeCN	-1.4	-1.6	-1.6
DLPNO-CCSD(T)/def2-TZVP/gas phase	1.0	0.8	0.8
DLPNO-CCSD(T)/def2-TZVP/MeCN	1.4	1.1	1.1

Coordinates for the optimized structures

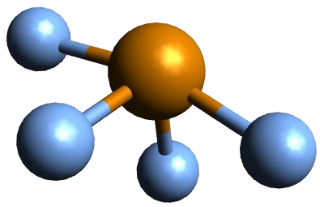

TeF_4

Te	-0.25218	0.16121	0.00000
F	-0.02080	0.01345	1.90544
F	1.51943	0.72524	0.00000
F	-0.02080	0.01345	-1.90544
F	-0.02080	-1.68358	-0.00000

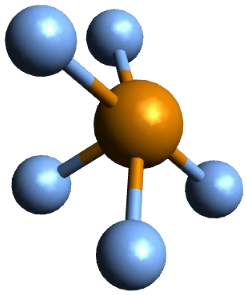

TeF_5^-

Te	0.00001	-0.00008	-0.26632
F	1.95883	0.19968	-0.02006
F	-1.95883	-0.19973	-0.02008
F	0.19972	-1.95873	-0.02004
F	-0.19970	1.95873	-0.02054
F	-0.00007	0.00052	1.61946

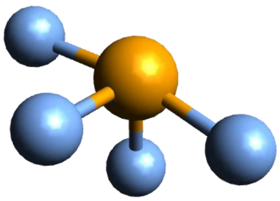

SeF_4

Se	-0.00001	0.00004	-0.31924
F	0.00004	-1.30141	0.76491
F	1.77488	-0.00008	-0.16193
F	0.00001	1.30144	0.76496
F	-1.77490	-0.00011	-0.16195

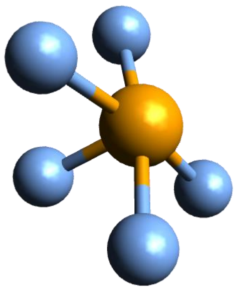

SeF_5^-

Se	0.00020	0.00012	-0.26863
F	0.68184	1.71409	-0.11040
F	-0.68166	-1.71250	-0.10928
F	-1.71427	0.68004	-0.10989
F	0.00066	0.00124	1.45438
F	1.71269	-0.68331	-0.10998

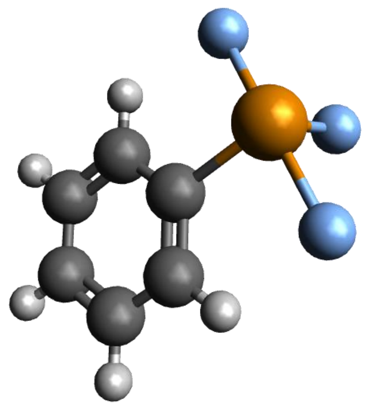

$PhTeF_3$

Te	1.17702	-0.00000	-0.26514
F	1.13417	-1.95414	-0.06189
F	1.64708	0.00000	1.55486
F	1.13417	1.95414	-0.06189
C	-0.91725	-0.00000	-0.08193
C	-1.60110	-1.21034	-0.03614
C	-1.60110	1.21034	-0.03614
C	-2.98249	-1.20219	0.06869
C	-2.98249	1.20219	0.06869
C	-3.67129	0.00000	0.12023
H	-1.06018	2.14383	-0.06881
H	-3.51968	2.13923	0.11174
H	-4.74972	0.00000	0.20114
H	-3.51968	-2.13923	0.11174
H	-1.06018	-2.14383	-0.06881

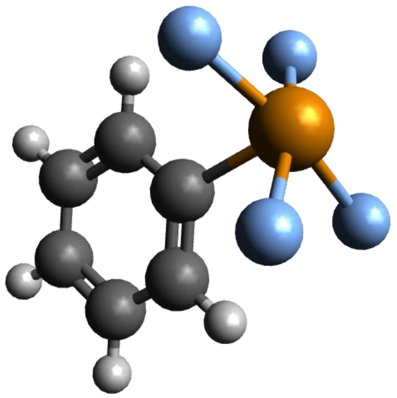

$PhTeF_4^-$

Te	1.18969	0.00000	-0.00000
F	0.99221	1.43619	1.40841
F	0.99221	1.43621	-1.40839
F	0.99221	-1.43619	-1.40840
F	0.99221	-1.43621	1.40838
C	-0.93275	0.00000	0.00000
C	-1.62599	1.20077	0.00000
C	-1.62599	-1.20078	-0.00000
C	-3.01362	1.19963	0.00000
C	-3.01361	-1.19964	-0.00000
C	-3.71020	-0.00000	0.00000
H	-1.07708	-2.13105	-0.00000
H	-3.55161	-2.13939	-0.00000
H	-4.79328	-0.00000	0.00000
H	-3.55161	2.13938	0.00001
H	-1.07709	2.13105	0.00000

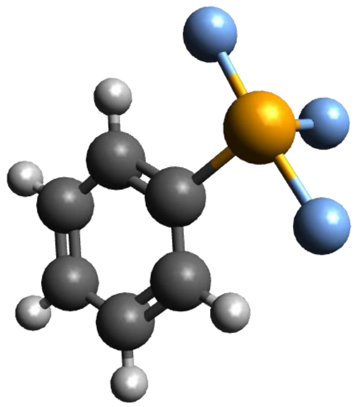

$PhSeF_3$

C	-0.10686	1.29911	1.21074
C	-0.05592	2.68319	1.20103
C	-0.12581	0.61837	0.00000

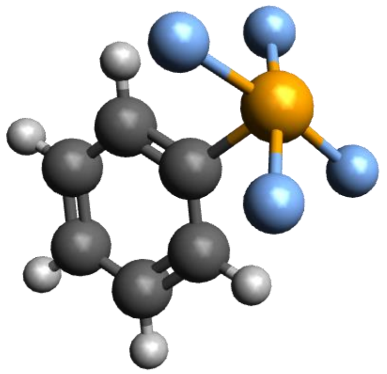

$PhSeF_4^-$ gas phase

Se	1.28745	0.00003	0.00000
F	1.17413	1.34272	1.32816
F	1.17413	1.34274	-1.32815

C	-0.03057	3.37447	0.00000	F	1.17422	-1.34275	1.32806
C	-0.05592	2.68319	-1.20103	F	1.17422	-1.34274	-1.32807
C	-0.10686	1.29911	-1.21074	C	-0.66068	-0.00000	-0.00000
F	-0.10686	-1.33951	1.81992	C	-1.34363	1.20291	0.00000
F	1.44136	-1.71104	-0.00000	C	-1.34360	-1.20293	-0.00001
F	-0.10686	-1.33951	-1.81992	C	-2.73089	1.19948	0.00000
Se	-0.23084	-1.31306	-0.00000	C	-2.73086	-1.19953	-0.00001
H	-0.12239	0.75744	2.14227	C	-3.42754	-0.00003	-0.00000
H	-0.03580	3.21988	2.13898	H	-0.78670	2.12679	0.00001
H	-0.12239	0.75744	-2.14227	H	-3.26827	2.13942	0.00001
H	0.00779	4.45514	0.00000	H	-4.51048	-0.00004	-0.00000
H	-0.03580	3.21988	-2.13898	H	-3.26822	-2.13948	-0.00001
				H	-0.78665	-2.12680	-0.00001

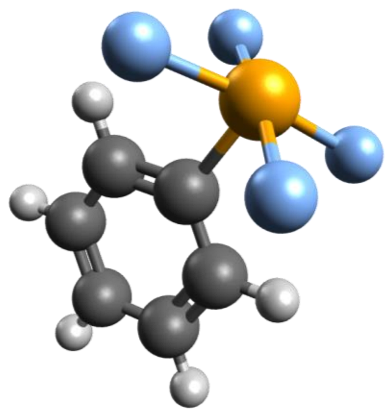

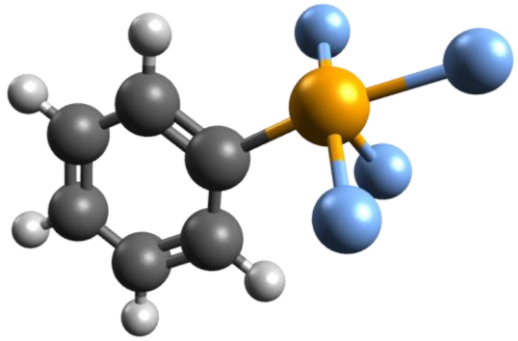

**PhSeF$_4^-$ in THF**				**PhSeF$_4^-$ 2nd conformer in THF**			
Se	1.27933	0.00001	-0.00000	Se	1.27933	0.00001	-0.00000
F	1.16924	1.34289	1.33088	F	1.16924	1.34289	1.33088
F	1.16933	1.33305	-1.34076	F	1.16933	1.33305	-1.34076
F	1.16940	-1.33301	1.34074	F	1.16940	-1.33301	1.34074
F	1.16922	-1.34287	-1.33087	F	1.16922	-1.34287	-1.33087
C	-0.65695	-0.00000	0.00000	C	-0.65695	-0.00000	0.00000
C	-1.33908	1.20504	0.00251	C	-1.33908	1.20504	0.00251
C	-1.33906	-1.20507	-0.00249	C	-1.33906	-1.20507	-0.00249
C	-2.72660	1.20089	0.00264	C	-2.72660	1.20089	0.00264
C	-2.72658	-1.20095	-0.00265	C	-2.72658	-1.20095	-0.00265
C	-3.42171	-0.00003	-0.00001	C	-3.42171	-0.00003	-0.00001
H	-0.79383	2.13778	0.00464	H	-0.79383	2.13778	0.00464
H	-3.26344	2.14211	0.00482	H	-3.26344	2.14211	0.00482
H	-4.50541	-0.00004	-0.00002	H	-4.50541	-0.00004	-0.00002
H	-3.26340	-2.14218	-0.00484	H	-3.26340	-2.14218	-0.00484

H	-0.79379	-2.13780	-0.00461	H	-0.79379	-2.13780	-0.00461

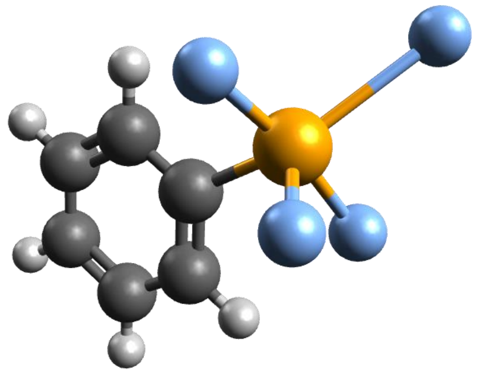				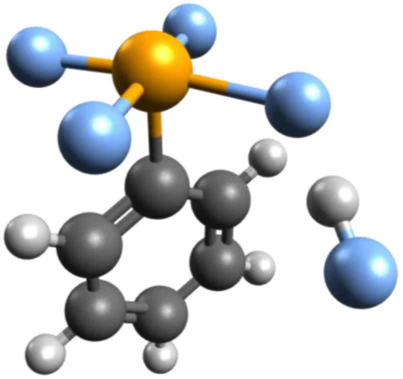			
**TS($PhSeF_4^-$ conf 1-> conf2) in THF**				**[$PhSeF_4^-$ +HF]**			
Se	2.28509	-0.12777	0.11122	Se	1.19872	-0.22210	0.18018
F	2.26687	1.39512	1.13144	F	1.09621	0.90066	1.67421
F	2.64700	0.95617	-1.19949	F	1.26824	1.25762	-0.84799
F	4.19908	-0.95523	1.79289	F	0.91778	-1.90646	1.38864
F	2.20990	-1.43340	-1.22380	F	1.13566	-1.25827	-1.38922
C	0.36027	-0.02766	0.00737	C	-0.73351	-0.18518	0.11222
C	-0.29247	1.19333	0.10741	C	-1.39024	1.00893	0.35776
C	-0.34827	-1.21234	-0.13848	C	-1.43248	-1.33686	-0.21045
C	-1.67599	1.22492	0.03834	C	-2.77443	1.04930	0.27097
C	-1.73360	-1.16972	-0.18817	C	-2.81565	-1.28654	-0.29032
C	-2.39571	0.04624	-0.10486	C	-3.48751	-0.09549	-0.05271
H	0.27046	2.10751	0.23406	H	-0.83211	1.89811	0.61456
H	-2.19271	2.17477	0.10185	H	-3.29365	1.98165	0.45715
H	-3.47782	0.07668	-0.15080	H	-4.56838	-0.05973	-0.12068
H	-2.29272	-2.09082	-0.29718	H	-3.36848	-2.18360	-0.54184
H	0.17061	-2.15780	-0.22183	H	-0.90528	-2.26148	-0.39532
				H	0.07844	-1.89817	2.55506
				F	-0.48960	-1.91446	3.34544

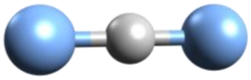			
**FHF^-**			
F	0.14356	-3.095709	2.01115
H	-0.49165	-2.404003	2.65286
F	-1.12973	-1.714616	3.29347

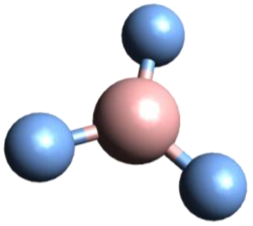

BF_3

B	-0.00000	0.00001	0.00000
F	0.69924	1.11151	0.00000
F	0.61297	-1.16130	0.00000
F	-1.31221	0.04979	0.00000

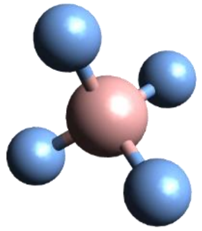

BF_4^-

B	0.00001	-0.00001	-0.00003
F	-1.26358	0.26746	-0.56262
F	0.03975	0.49086	1.31988
F	0.22512	-1.39064	0.00918
F	0.99871	0.63232	-0.76643

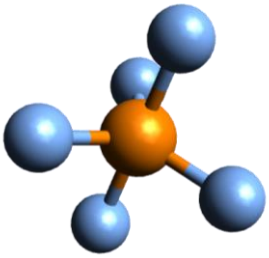

PF_5

P	-0.00000	0.00005	0.00004
F	0.00004	1.42504	0.61071
F	1.58865	-0.00011	0.00004
F	0.00002	-0.18351	-1.53938
F	-1.58865	-0.00004	0.00001
F	-0.00005	-1.24145	0.92856

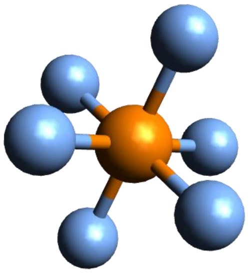

PF_6^-

P	-0.00001	0.00001	0.00001
F	-0.85950	-0.21139	1.36564
F	0.02709	1.60537	0.26551
F	1.38166	-0.16296	0.84431
F	0.85949	0.21137	-1.36563
F	-1.38166	0.16295	-0.84433
F	-0.02707	-1.60536	-0.26552

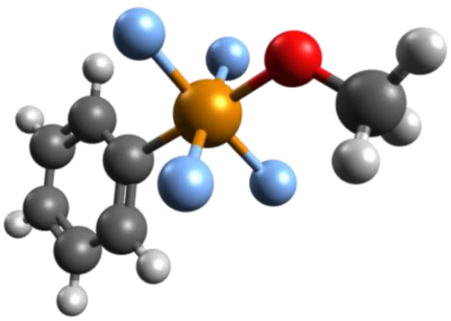

trans-$PhTeF_4(OMe)$

Te	-0.69535	-0.12981	0.02153

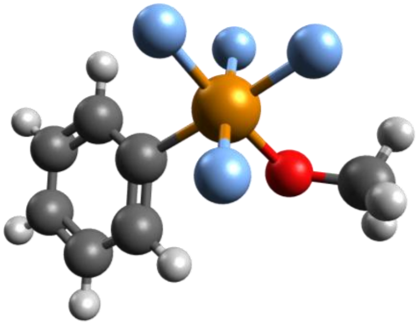

cis-$PhTeF_4(OMe)$

Te	-0.74138	-0.23092	0.07167

F	-0.62625	-1.49395	1.31897	F	-0.78758	-1.16759	-1.56577
F	-0.81844	1.20998	1.36493	F	-0.90192	0.57118	1.77681
F	-0.64783	-1.45006	-1.32762	F	-0.56375	-1.89811	0.92924
F	-0.86494	1.23728	-1.28977	C	1.32902	0.03164	-0.00673
O	-2.57186	-0.33515	0.07516	C	2.11891	-1.01887	-0.44248
C	-3.44442	0.77318	-0.14843	C	3.49238	-0.83860	-0.48428
H	-4.43262	0.41113	0.12575	C	1.86355	1.24619	0.38891
H	-3.43813	1.07168	-1.19496	C	3.23908	1.40865	0.34179
H	-3.18383	1.62155	0.48269	C	4.04828	0.37019	-0.09360
C	1.38951	0.03856	-0.00302	H	1.22569	2.04976	0.72631
C	1.95794	1.30009	0.02439	H	3.67633	2.34888	0.64660
C	3.34027	1.39918	0.00666	H	4.12641	-1.64602	-0.82207
C	2.14765	-1.11862	-0.04697	H	5.12067	0.50367	-0.12788
C	3.52848	-0.99971	-0.06327	H	1.67823	-1.95789	-0.74395
C	4.12049	0.25380	-0.03672	O	-1.05355	1.44227	-0.78622
H	1.67912	-2.09142	-0.06842	C	-2.32433	2.08770	-0.79378
H	4.13953	-1.89051	-0.09692	H	-2.15314	3.06801	-1.23362
H	3.80500	2.37471	0.02735	H	-2.71544	2.20793	0.21614
H	5.19825	0.33866	-0.04990	H	-3.04089	1.53610	-1.40001
H	1.34333	2.18731	0.05918	F	-2.58446	-0.54570	0.17556

References

[1] M. D. Böhme, T. Eder, M. B. Röthel, P. D. Dutschke, L. F. B. Wilm, F. E. Hahn, F. Dielmann, *Angew. Chem. Int. Ed.* **2022**, e202202190.

[2] F. Buß, C. Mück-Lichtenfeld, P. Mehlmann, F. Dielmann, *Angew. Chem. Int. Ed.* **2016**, *57*, 4951–4955.

[3] N. Jalalian, B. Olofsson, *Org. Synth*. **2013**, *90*, 1–9.

[4] W. Gombler, J. Schaebs, H. Willner, *Inorg. Chem.* **1990**, *29*, 2697–2698.

[5] SAINT version 7.46a, Bruker AXS Inc. Madison **2004**.

[6] G. Sheldrick GM SADABS. University of Göttingen, Göttingen **1996**.

[7] O. V. Dolomanov, L. J. Bourhis, R. J. Gildea, J. A. K. Howard, H. Puschmann, J. *Appl. Cryst.* **2009**, *42*, 339–341.

[8] M. C. Burla, R. Caliandro, M. Camalli, B. Carrozzini, G. L. Cascarano, L. De Caro, C. Giacovazzo, G. Polidori, R. Spagna, *J. Appl. Cryst.* **2005**, *38*, 381–388.

[9] G. M. Sheldrick, *Acta Cryst.*, **2015**, *A71*, 3–8.

[10] G. M. Sheldrick, *Acta Cryst.*, **2015**, *C71*, 3–8.

[11] M. J. Frisch, G. W. Trucks, H. B. Schlegel, G. E. Scuseria, M. A. Robb, J. R. Cheeseman,G. Scalmani, V. Barone, G. A. Petersson, H. Nakatsuji, X. Li, M. Caricato, A. V. Marenich, J. Bloino, B. G. Janesko, R. Gomperts, B. Mennucci, H. P. Hratchian, J. V. Ortiz, A. F. Izmaylov, J. L. Sonnenberg, D. Williams-Young, F. Ding, F. Lipparini, F.

Egidi, J. Goings, B. Peng, A. Petrone, T. Henderson, D. Ranasinghe, V. G. Zakrzewski, J. Gao, N. Rega, G. Zheng, W. Liang, M. Hada, M. Ehara, K. Toyota, R. Fukuda, J. Hasegawa, M. Ishida, T. Nakajima, Y. Honda, O. Kitao, H. Nakai, T. Vreven, K. Throssell, J. A. Montgomery, Jr., J. E. Peralta, F. Ogliaro, M. J. Bearpark, J. J. Heyd, E. N. Brothers, K. N. Kudin, V. N. Staroverov, T. A. Keith, R. Kobayashi, J. Normand, K. Raghavachari, A. P. Rendell, J. C. Burant, S. S. Iyengar, J. Tomasi, M. Cossi, J. M. Millam, M. Klene, C. Adamo, R. Cammi, J. W. Ochterski, R. L. Martin, K. Morokuma, O. Farkas, J. B. Foresman, and D. J. Fox, *Gaussian 09, Revision A.03, Gaussian, Inc.: Wallingford CT*, **2016**.

[12] J.-D. Chai, M. Head-Gordon, *Phys. Chem. Chem. Phys.* **2008**, *44*, 6615–6620.

[13] T. H. Dunning Jr. *J. Chem. Phys.*, **1989,** *90*, 1007-1023.

[14] K. A. Peterson, D. Figgen, E. Goll, H. Stoll, M. Dolg. *J. Chem. Phys.* **2003,** *119*, 11113-11123.

[15] F. Weigend, R. Ahlrichs. *Phys. Chem. Chem. Phys.***2005**, *7*, 3297-3305.

[16] F. Neese. *WIREs Comput Mol Sci.***2022**, *12*, e1606.

[17] C. Riplinger, B. Sandhoefer, A. Hansen, F. Neese. *J. Chem. Phys.* **2013**, *139*, 134101.

[18] C. Riplinger , F. Neese. *J. Chem. Phys.* **2013**, *138*, 034106.

[19] H. Ryu, H. Kim, J. Park, S. Kim, M. H. Baik, M. *Organometallics* **2018**, *37*, 3228–3239.

[20] K. O. Christe, D. A. Dixon, D. McLemore, W.W. Wilson, J. A. Sheehy, J. A. Boatz, *J. Fluorine Chem.* **2000**, *101*, 151–153.
